# What Factors Influence Head Acceleration During a Purposeful Header in Soccer Players? A Systematic Review

**DOI:** 10.1007/s40279-025-02209-2

**Published:** 2025-04-24

**Authors:** Rebecca Blyth, Gerard Farrell, Anja Zoellner, Osman Hassan Ahmed, Melanie Bussey, Olivia Galea, Gisela Sole

**Affiliations:** 1 Centre for Health, Activity, and Rehabilitation Research, School of Physiotherapy, University of Otago, 325 Great King Street, Dunedin, 9016 New Zealand; 2 School of Sport and Recreation, Sports Performance Research Institute New Zealand, Auckland University of Technology, Auckland, New Zealand; 3 University Hospitals Dorset NHS Foundation Trust, Poole, UK; 4 School of Sport, Health and Exercise Science, University of Portsmouth, Portsmouth, UK; 5 The FA Centre for Para Football Research, Burton-Upon-Trent, UK; 6 School of Physical Education, Sport, and Exercise Science, University of Otago, Dunedin, New Zealand

## Abstract

**Background:**

Head acceleration is often used as a proxy measurement for concussion risk. It is unclear what factors contribute to head acceleration during a purposeful header.

**Objective:**

The objective of this systematic is to identify what factors influence head acceleration during a header and highlight areas that have not been explored.

**Methods:**

Studies were included if they assessed the effect of an independent variable on head acceleration during a purposeful header. There were no exclusion criteria relating to age, sex, playing level, study design, or publication date. Databases included Scopus, Web of Science, MEDLINE, EMBASE, CINAHL, SPORTDiscus and ClinicalKey, and grey literature was also searched. Searches were re-run in January 2024. Each included study underwent a risk of bias or quality assessment, using several tools owing to varied study designs (ROBINS-I, RoB2, modified Downs and Black and AXIS). Results are presented in tabular form, categorised by independent variable(s) and accompanied by a narrative results section.

**Results:**

A total of 60 studies were included. Study designs included one-off measurements with variable(s), cohort, cross-sectional and randomised controlled trials (RCTs). Influencing factors included ball velocity and characteristics, header type, impact location, neck training interventions, fatigue protocols, neck strength, demographics, playing scenario and personal protective equipment. A wide range of heading trials and methods of measuring head acceleration were used. Results were conflicting for several variables. There was some consistent evidence that increased ball speed/mass/inflation increased head acceleration. Female players sustained greater head accelerations than males, and some evidence suggested that increased neck strength reduced head acceleration, but not in all cases. This review confirmed traditional neck strengthening is not effective in reducing head acceleration; however, adding neuromuscular exercises appeared to be effective. There was a lack of evidence investigating other systems that may affect head acceleration such as vestibular function, oculomotor performance, rate of force development and cervical spine proprioception.

**Discussion:**

This review highlights the range of studies investigating head acceleration during a purposeful header. Included studies were of varying quality, and one limitation was the lack of transformation of the acceleration measures to a consistent frame of reference. Implications from this study suggest potential measures that may reduce head acceleration and therefore improve the safety of performing a purposeful header. These include reducing ball inflation pressure, limiting headers from long range/high speed balls (especially in children/adolescents and females) and ensuring neuromuscular exercises are incorporated into neck training programmes. Dynamic muscle strength outcomes, including measures such as rate of force development, are challenging to measure but should be considered an essential component of neck training when the aim is to reduce head acceleration in a ballistic task such as a header.

The review is registered under the PROSPERO registry (CRD42022359294).

**Supplementary Information:**

The online version contains supplementary material available at 10.1007/s40279-025-02209-2.

## Key Points

**Table:** 

A range of intrinsic and extrinsic variables affect head acceleration during a purposeful header.
Ball properties and ball speed along with the type of ball delivery all affect head acceleration.
While neck strength may influence head acceleration, traditional neck strengthening exercises do not.
Incorporating neuromuscular neck exercises appears to reduce head acceleration during a header.

## Introduction

### Rationale

Soccer is unique as the only sport where players intentionally use their head to pass, shoot or clear the ball. Performance of purposeful headers has recently come under scrutiny with respect to player welfare, and this concern is twofold. First, there is a potential relationship between headers and the high rates of concussion seen in soccer [1]. Most commonly, acute concussions in soccer are related to opponent contact (49.6%) such as head-to-body (27.9%) or head-to-head contact (21.7%) [2], often when players compete for an aerial ball. However, concussion can also occur from ball-to-head contact, with a study of collegiate athletes showing 32.7% of concussions were caused by this mechanism (both intentional and unintentional ball-to-head contact) [2]. Second, concern has been raised about the potential long-term effects on brain health from repetitive ball-to-head impacts, which is not yet fully understood [3, 4].

Heading the ball involves head impact and acceleration and deceleration of the brain inside the skull, and it may also cause rotation of the brain [5]. While studies in other sports have linked increasing head acceleration to an increased risk of concussion [6, 7], the factors influencing head acceleration leading to an increased risk of concussion have not been fully evaluated. Considering the number of headers a player performs over their playing career, and the concern around potential long-term consequences (from overall heading burden) [8–11], understanding factors that are causing greater burden (i.e. greater magnitude impacts) will help to inform risk reduction programmes and potential rule changes to improve player safety.

Neck strength has recently been proposed as a possible factor influencing head acceleration [12]. Neck strength is suggested to influence concussion risk, although evidence is conflicting. Literature supporting this is viewed as ‘emerging’ [13] or ‘low certainty’ [14]. Systematic reviews investigating the primary prevention of concussion conclude that neck strength and head acceleration show potential value for concussion prevention. They also highlight the need to further investigate the relationship between neck strength and head acceleration with concussion risk [15, 16]. It may also be that neuromuscular performance of neck muscles or other features of sensorimotor performance are relevant to head acceleration, especially in response to the ballistic task of heading a soccer ball. Recent studies point to the importance of neuromuscular exercises and considering the rate of force development when aiming to alter head kinematics and reduce head acceleration [17, 18]. Synthesis of current data relating to neck strength, morphology and neuromuscular or sensorimotor performance on head acceleration needs to be further investigated in the context of purposeful heading.

Other factors that might be important influencers of head acceleration in response to heading are sex, head impact mechanism and extrinsic factors such as ball speed and ball characteristics. As discussed, both ball-to-head contact and contact with an opponent while contesting for a header may cause concussive events [19]. In high school players, heading was associated with 28% of concussions in boys and 26.5% of concussions in girls [19]. Of these heading-related concussions, most were related to player-to-player contact for boys, and only 23.6% were due to ball contact [19]. In contrast, for high school girls, over 50% of heading-related concussions were due to ball contact [19]. Female soccer players sustain a greater rate of concussions compared with their male counterparts [20], have greater head acceleration during a header and are more likely to sustain a concussion from a ball-to-head impact [21]. Female sex is a known risk factor that has shown a correlation with increased risk of concussion [14, 20], and it is possible that the increased head acceleration may play a role in this. Systematically reviewing the results of studies investigating these individual risk factors will provide more conclusive insights about influencing factors for head acceleration during heading.

There is uncertainty around what factors influence head acceleration during a header (and therefore influence header safety). Considering the concern due to both the relationship between head acceleration and concussion risk and the potential long-term effects due to repeated exposure to head accelerations, this is important to understand. To develop risk-reduction strategies and training interventions for heading and reduce overall heading burden over a playing career, we must first understand what factors influence head acceleration during a purposeful header. Understanding this will allow a critical step forward in developing targeted training interventions with the goal of improving header safety.

### Objectives

This systematic review aims to synthesise results of existing literature investigating the influence of intrinsic and extrinsic variables on head acceleration during a purposeful header in soccer players. The specific research question is: what factors influence head acceleration during a purposeful header in soccer players?

For this review, a purposeful header is defined as the intentional act of the player using their head to redirect the ball.

## Methods

A systematic review was undertaken following best evidence-based practice guidelines, including the Preferred Reporting for Systematic Review and Meta-analysis (PRISMA) statement [22] and Joanna Briggs institution (JBI) review manual [23]. The objectives, inclusion criteria and methods of analysis for this review were specified in advance and documented in an a priori protocol [23] registered under the PROSPERO registry (CRD42022359294).

### Eligibility Criteria

Longitudinal, experimental, observational and cross-sectional studies published in any language, with no restriction on publication year, were eligible for inclusion. To be included, studies must have: (i) included a population of soccer players (any age, sex or skill level); (ii) measured head acceleration during a purposeful header using either an instrumented mouth guard, an accelerometer fixed to the participant’s head or video analysis (motion capture or two-dimensional (2D) video analysis); (iii) measured head acceleration during specific header trials and, if measured during ‘live’ trainings or games, a specific analysis or breakdown showing acceleration during purposeful headers was reported or headers/ball-to-head impacts made up the large majority of impacts; (iv) had an aim of the study to investigate relationships between intrinsic or extrinsic factors and head acceleration and (v) had the full-text paper available, including theses and conference papers. Studies were excluded if: (i) they were of qualitative design; (ii) they did not differentiate the analysis of purposeful header impacts from other incidental head impacts, such as contact with the ground or opposing players or (iii) they were review papers, posters, conference abstracts or books.

### Information Sources

Databases including Scopus, Web of Science, MEDLINE, EMBASE, CINAHL, SPORTDiscus and ClinicalKey were initially searched between 20 September 2023 and 30 September 2023. Grey literature was searched using Google, MedNar, TRIP and clinical trial sites search engines (Table 1). Reference lists and citations of eligible studies were also searched and screened. The lead author re-ran and screened the main databases just prior to the final analysis and write-up to check for new papers eligible for inclusion (with the most recent search undertaken on 28 January 2024).

**Table 1:** Information sources searched

Database	Initial search date	Search alert set and reviewed	Dates of search re-run (new records screened)
Scopus	20 September 2022	Yes	28 January 2024 (147) 20 January 2023 (29)
Web of Science	20 September 2022	Yes	16 January 2024 (266) 20 January 2023 (173)
MEDLINE	20 September 2022	Yes	16 January 2024 (74) 20 January 2023 (27)
EMBASE	20 September 2022	Yes	16 January 2024 (127) 20 January 2023 (56)
CINAHL	20 September 2022	Yes	16 January 2024 (39) 20 January 2023 (6)
SPORTDiscus	20 September 2022	Yes	16 January 2024 (51) 20 January 2023 (12)
ClinicalKey	20 September 2022	No	Not re-run 2024 owing to change in database 20 January 2023 (2)
Google	30 September 2022	No	19 January 2023 (280) 24 January 2023 (8)
MedNar	23 September 2022	No	18 January 2024 (28) 24 January 2023 (17)
TRIP	30 September 2022	No	18 January 2024 (109) 24 January 2023 (49)
Clinical trial sites*	23 September 2022	No	Not re-run

^*^Australian New Zealand Clinical Trials Registry (ANZCTR), EU Clinical Trials Register, International Clinical Trials Registry Platform (ICTRP) hosted by the World Health Organisation (WHO) **and** Clinical Trials

### Search Strategy

The search strategy was developed in consultation with a medical librarian and reviewed following an initial database search, following which new keywords were added and searches re-run on each database. No limits or filters were placed on main database searches. Similar strategies were used for grey literature searching, but filters could be applied. The final search strategies for each database and filters applied for grey literature search engines can be found in Appendix I. Table 2 presents the search strategy keywords.

**Table 2:** Search strategy

Concept 1	Concept 2
“head acceleration” OR “head impact*” OR “head impact magnitude” OR “head kinematic*” OR “head movement*” OR “head stabil*” OR “head motion” OR “head velocity” OR header* OR heading OR “purposeful header*” OR “purposeful heading”	soccer OR football

### Selection Process

#### Data Management

All results from database searching were exported to EndNote20 (Clarivate Analytics, Pennsylvania, USA), and once searching was complete, records were de-duplicated within EndNote. Following de-duplication, results were exported to Rayyan (Rayyan Systems Inc., Cambridge, Massachusetts, USA). Two reviewers (R.B. and G.F.) then screened the titles and abstracts with the blinding feature enabled to ensure an independent review and screening process.

#### Selection Process

The two reviewers (R.B. and G.F.) met prior to screening to discuss inclusion criteria and clarify understanding of the selection process. Following screening, any conflicts were resolved following discussions between the two reviewers. Following title/abstract screening, full texts of papers were retrieved where available. Where full texts could not be accessed, authors or universities were contacted. Once located, full texts were screened by two independent reviewers (R.B. and G.F.) to be deemed eligible for inclusion in the review.

### Data Collection Process

Two independent researchers (R.B. and G.F.) extracted data for a random sample of the eligible studies using the data extraction tool (Appendix II). The tool was developed by the lead researcher and reviewed by the review team. A random number generator was used to select six studies which represented 11% of initially included studies. Following the independent review, the two researchers met to check consensus of the data extracted from the sample of studies. Once agreement concerning data extraction was confirmed, the lead researcher (R.B.) extracted data from all remaining studies, and the second reviewer (G.F.) checked all data extraction for accuracy once completed. Where there was disagreement, this was resolved via discussion between the two reviewers. Data were extracted from each full text deemed eligible for inclusion in the review.

#### Data Items

Data were sought and charted for items presented in Table 3.

**Table 3:** Data items

1	Citation details: author(s), date, title, journal, volume, issues and pages
2	Study design and setting
3	Study aim/purpose
4	Participants: the age, sex and playing level of the soccer players and the number of participants in the study
5	Participant recruitment procedures utilised
6	Exposure: the method of performing a purposeful header
7	The factor that the study is investigating in relation to head acceleration
8	Outcomes: how head acceleration was measured and the resulting acceleration
9	Measurement of head acceleration during a purposeful header and the relationship of this measurement with the influencing factor assessed by the study. Linear acceleration (LA), rotational/angular acceleration (RA/AA) and rotational velocity (RV) were all outcomes of interest
10	Any data analysis methods including statistical techniques

### Risk of Bias/Quality Assessment

Risk of bias (RoB) or quality assessment were performed by two independent reviewers (R.B. and A.Z.) using an appropriate RoB or quality assessment tool for each included study. Any disagreements were resolved via discussion. Owing to different designs of eligible studies, a single RoB tool could not be used for all studies without significant modification of the tool. In total, four tools were required to assess RoB or study quality; Appendix III defines each study design to determine which tool was used to assess methodological quality. A copy of each tool can be found in Appendix IV.

- The *ROBINS-I tool* [24] was used for non-randomised trials of an intervention. The ROBINS-I tool views each study as an attempt to mimic a hypothetical RCT and covers seven domains of study bias [24]. The ROBINS-I tool is specific to assessing one outcome, which, for this review, was head acceleration.
- The *RoB2 tool* [25] was used to assess RCTs. The RoB2 tool assesses five domains of bias and is a revised version of the Cochrane RoB tool for RCTs [25].
- A modified version of the *Downs and Black tool* [26] was used for cohort and one-off measurement studies involving different variables. This tool was not initially listed in the protocol, but after the search was completed, it was added as the most appropriate tool for the listed study designs. The Downs and Black tool was modified by the lead researcher to remove or adapt questions that were not suitable for the included studies. The modified version (Appendix IV) was then piloted by a team member (G.S.) with experience in quality assessment tool modification processes, to ensure that it covered all appropriate quality assessment domains. Modification of the Downs and Black tool is commonly undertaken when assessing methodological quality of included studies in systematic reviews within the sports medicine field [27–29]. The modified version included 20 items marked as ‘yes’, ‘no’, ‘not applicable’ or ‘unable to determine’. To account for items marked as ‘not applicable’, each study was given a percentage score. Previous research using the full Downs and Black checklist (scored out of 28) defined scores as excellent (26–28), good (20–25), fair (15–19) and poor (< 14) quality [30]. Converting these scores to percentages for this systematic review, study quality was categorised as excellent (> 92%), good (71–91%), fair (53–70%) and poor (< 53%).
- The *AXIS tool* [31] was used for cross-sectional study designs, designed using a Delphi expert panel to assess the quality and RoB in cross-sectional studies [31]. As two of the items were marked as ‘not applicable’ for all included studies, the total score was out of 18. To be consistent with the other tools used for this systematic review, study quality was categorised as excellent (16–18), good (13–15), fair (10–12) or poor (< 10).

### Effect Measures

In cases where studies had not included statistical analysis but had provided raw non-transformed data for head acceleration (linear acceleration (LA) (g, m/s or m/s^2^), rotational acceleration (RA)/angular acceleration (AA) (rad/s^2^ or krad/s^2^ or deg/s^2^) and/or rotational velocity (RV)/angular velocity (AV) (rad/s or deg/s)), we calculated mean difference (MD) and 95% confidence intervals (CI) to evaluate the influence of factors being investigated on head acceleration [32]. In all other cases, the studies’ reported results were extracted and reported.

### Synthesis Methods

Following the study selection process and data extraction, studies were grouped into categories to synthesise the data. Results were presented in tabular format displaying the study characteristics, methods and results/conclusion. Owing to the large number of studies and the breadth of the results extracted, more detailed information was tabulated for display in Appendix V. Along with the tabular results, a narrative description compared and contrasted the results within each category.

### Reporting Bias Assessment and Certainty Assessment

Reporting bias was assessed in each of the RoB or quality assessment tools. Within the RoB/quality assessment tools, the outcomes and results presented by the authors were reviewed along with their reported methods to assess for any reporting bias.

The GRADE approach [33, 34] was used to rate the quality of evidence and to determine the strength of recommendations. The GRADE approach provides a structured and transparent process to rate the certainty of evidence in systematic reviews [34, 35]. Owing to the varied study designs included and the number of different RoB/quality assessment tools used, full GRADE assessment was not possible, but the framework was used to assess the overall quality of evidence.

## Results

### Study Selection

**Fig. 1:**
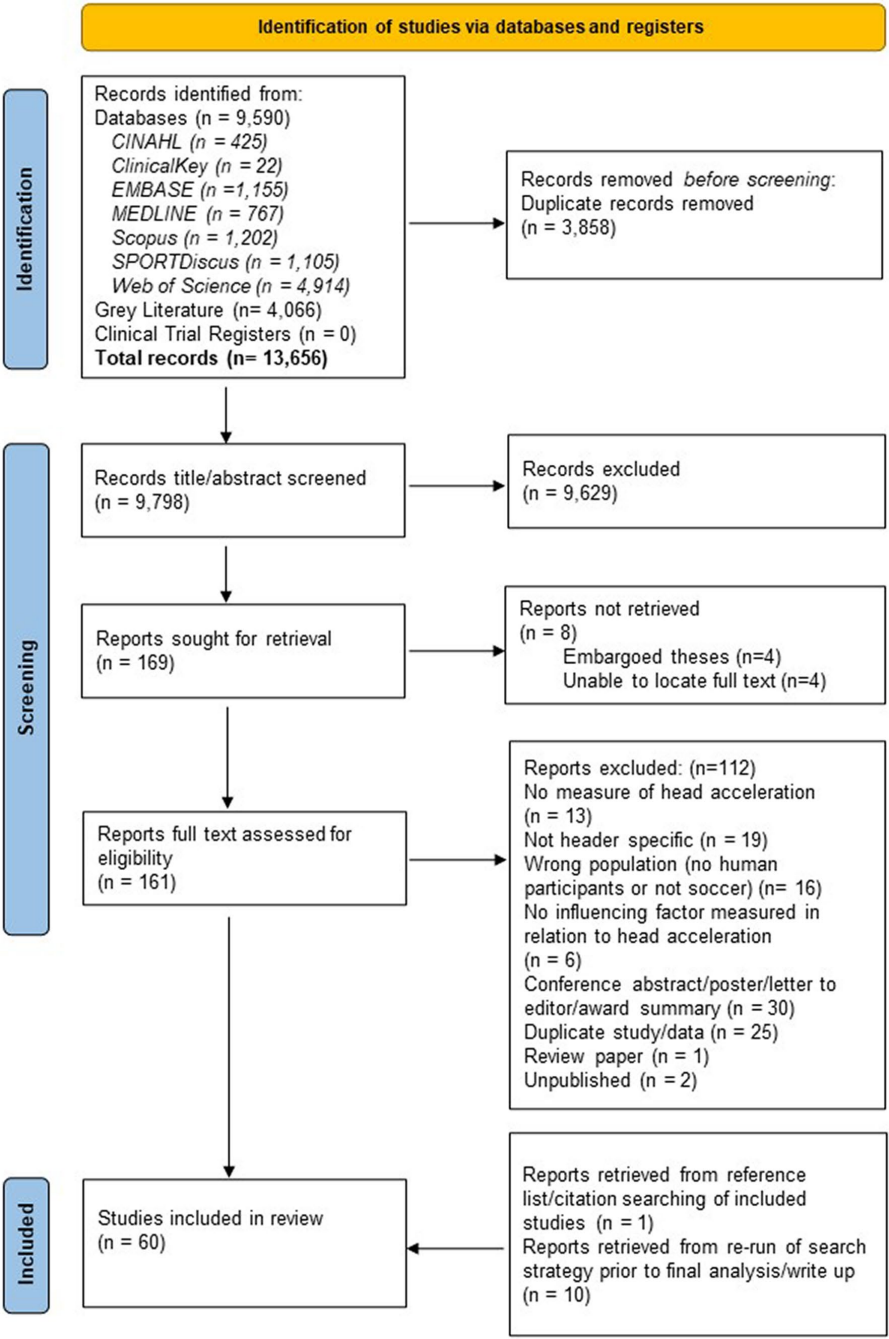
PRISMA flow chart [22]

In total, 60 papers were included in the review (Fig. 1). There was excellent agreement between the two reviewers (R.B. and G.F.), with 1.8% of citations being ‘conflict’ cases for title/abstract screening and 29 papers at the full-text screening stage. All conflicts were resolved with discussion. Of the 169 records deemed eligible for full text screening, 4 full-text papers [36–39] were unable to be located and 4 [40–43] were embargoed theses. Reasons for exclusion at the full-text stage can be seen in Fig. 1. Reference list and citation review of included articles located one further paper for inclusion, and re-running the search on each main database prior to final analysis located ten further papers. Meta-analyses were not possible owing to the heterogeneity of the study designs, independent and dependent variables (such as methods of ball delivery), head acceleration measurement methods and locations of the head/mouthguard mounted accelerometers without consistent transformation to a frame of reference. One author was contacted regarding a suspected transcription error in the results table. The correct value was provided by the author on reviewing the results table for reporting in this review. No non-English papers meeting the inclusion criteria were found. A total of six studies [44–49] were included that did not specifically differentiate between purposeful headers and other ball-to-head impacts. These studies were included on the basis of defining ball-to-head impacts separately to other head impacts (head-to-ground or player-to-player) and/or as the vast majority (89–95%) of the impacts recorded were headers. The very small number of incidental ball-to-head impacts (~ 10% or less) included in those studies would be unlikely to change the results. In cases where a range of different head impacts were included and differentiated, only the data for header impacts were extracted.

### Risk of Bias and Quality Assessments

**Fig. 2:**
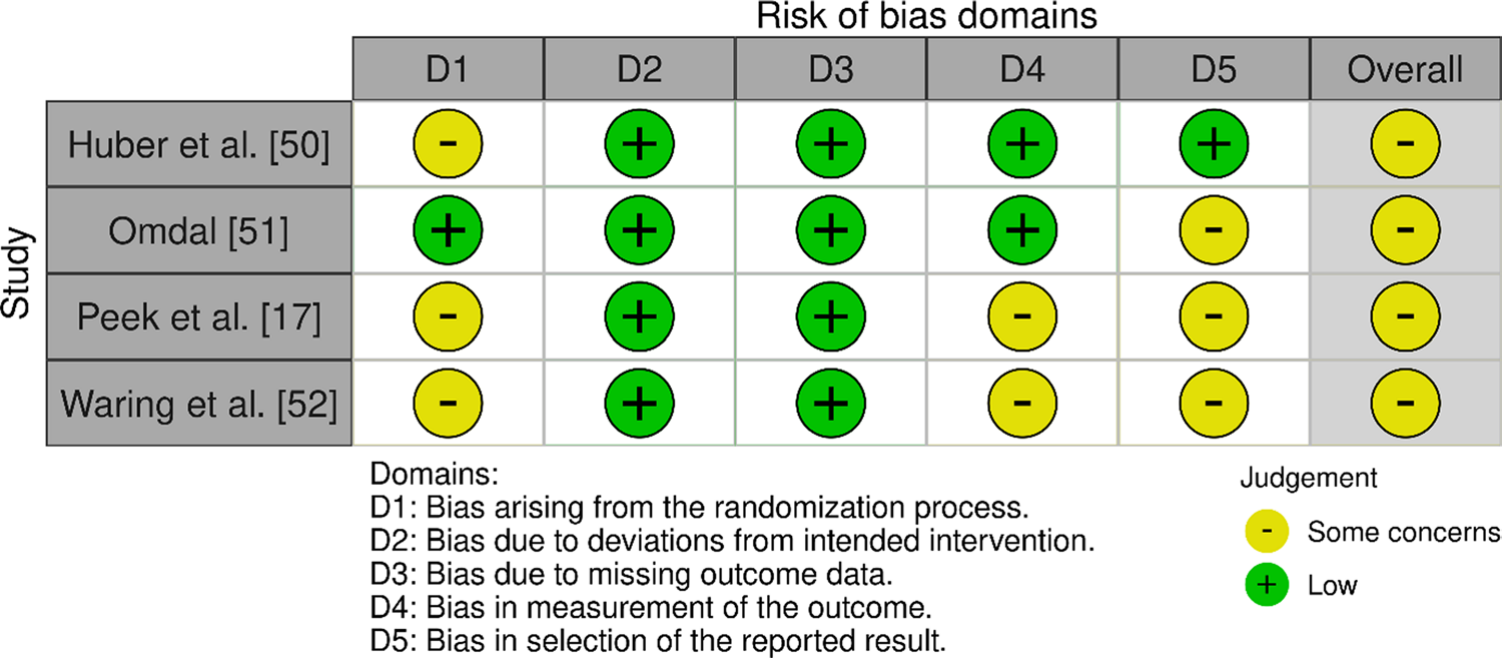
Risk of bias (ROB)2 assessments of RCTs [103]

**Fig. 3:**
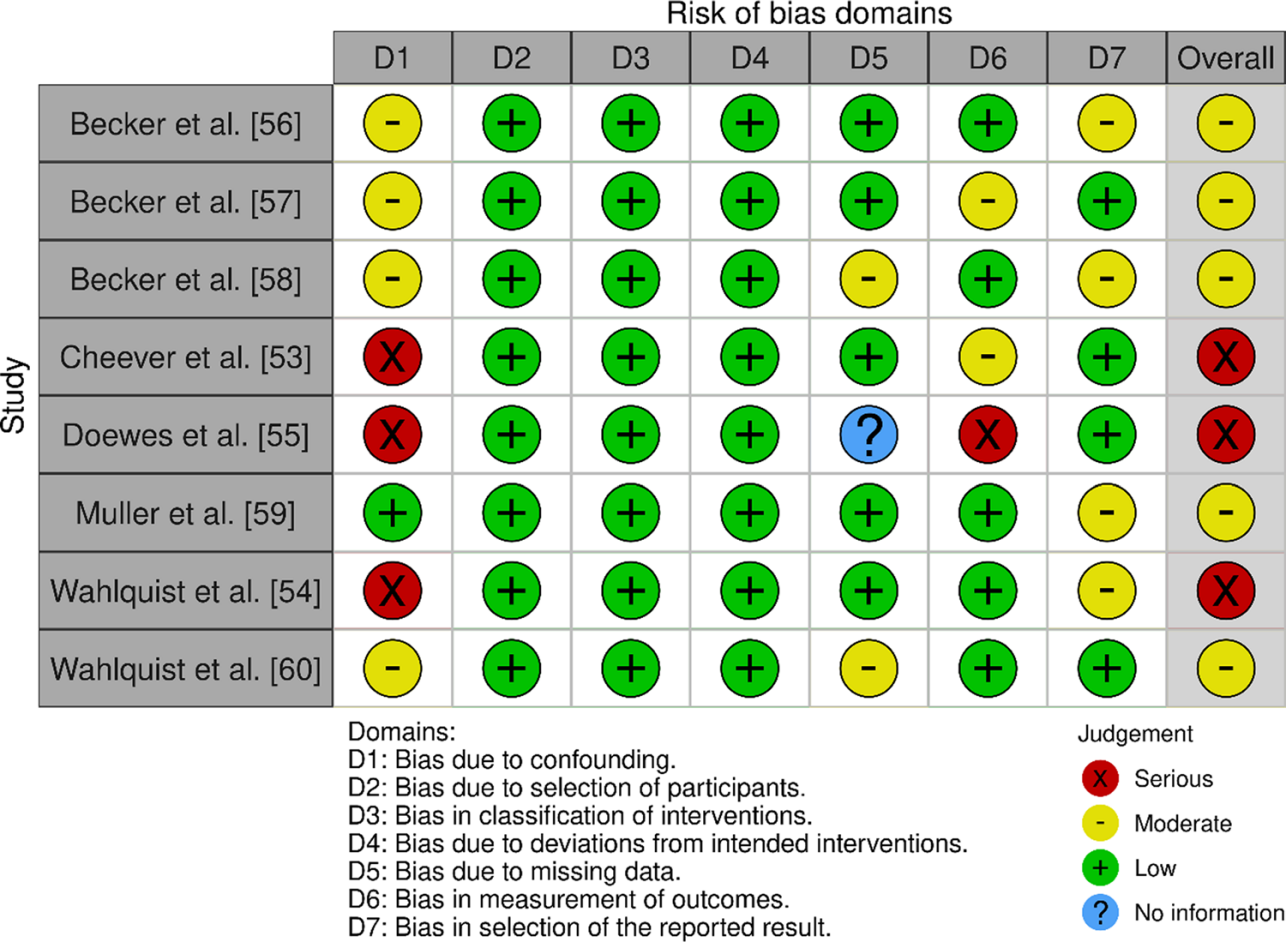
Risk of bias in non-randomised studies of interventions (ROBINS-I) assessments of non-randomised intervention studies [103]

**Fig. 4:**
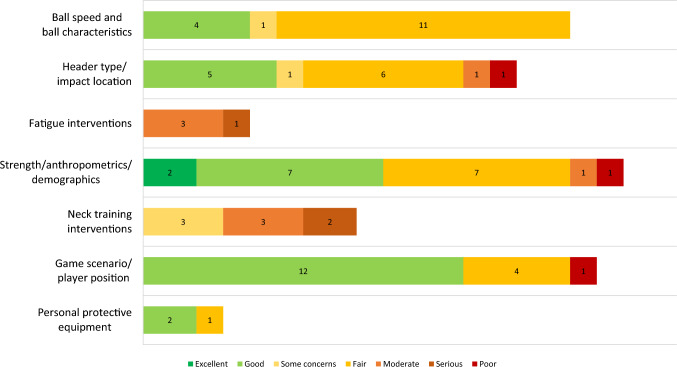
Risk of bias/quality assessment results shown grouped for the different variables explored. Numbers within the bars represent the number of studies

Results are presented in Figs. 2 and 3 and in Tables 4 and 5. Figure 4 shows the results of RoB and quality assessment grouped for each of the different independent variables. Considerable variability for RoB and quality assessment scoring across the papers was identified. All initial conflicts were resolved with discussion between the two independent reviewers (R.B. and A.Z.). The four RCT papers [17, 50–52] were all scored as ‘some concerns’, with bias around the randomisation and selection of the reported results being common issues. Of the seven studies assessed using the ROBINS-I tool, three studies [53–55] were considered at serious RoB, commonly owing to confounding factors, while the remaining five studies were all at moderate RoB [56–60].

**Table 4:** AXIS tool scores of cross-sectional studies

Question #	Bretzin et al. [63]	Caccese et al. [61]	Caccese et al. [62]	Dezman et al. [64]	Gutierrez et al. [65]	Ludwig [66]	Worsey et al. [67]
1	Y	Y	Y	Y	Y	Y	Y
2	Y	Y	Y	Y	Y	Y	Y
3	N	Y	N	N	N	N	N
4	Y	Y	Y	Y	Y	Y	N
5	Y	Y	Y	NR	Y	Y	N
6	NR	Y	Y	NR	N	NR	N
7	N/A	N/A	N/A	N/A	N/A	N/A	N/A
8	Y	Y	Y	Y	Y	Y	Y
9	NR	Y	Y	Y	Y	Y	Y
10	Y	Y	Y	Y	Y	Y	N
11	Y	N	Y	Y	Y	Y	N
12	N	Y	Y	Y	N	Y	Y
13^a^	N	N	N	N	N	N	N
14	N/A	N/A	N/A	N/A	N/A	N/A	N/A
15	NR	Y	Y	Y	Y	Y	Y
16	Y	Y	Y	Y	Y	N	Y
17	Y	Y	Y	Y	Y	Y	Y
18	Y	Y	Y	Y	Y	Y	N
19^a^	N	N	N	N	NR	NR	N
20	Y	Y	Y	Y	Y	Y	Y
Total (/18)	13	17	17	15	14	14	11
Quality	Good	Excellent	Excellent	Good	Good	Good	Fair

Y, yes (1); N, No (0); NR, not reported (0)

^a^Item is reverse scored—no is a positive**:** 1

**Table 5:** Modified Downs and Black scores of cohort and one-off measurement with variables studies

Study	% score	Quality	Study	% score	Quality
Austin et al. [85]	65%	Fair	Narimatsu et al. [77]	74%	Good
Barnes-Wood et al. [86]	67%	Fair	Naunheim et al. [95]	61%	Fair
Becker et al. [68]	72%	Good	Nevins et al. [46]	68%	Fair
Brooks et al. [69]	76%	Good	Peek et al. [78]	90%	Good
Caccese et al. [70]	88%	Good	Pereira et al. [79]	85%	Good
Chrisman et al. [44]	79%	Good	Pritchard et al. [47]	61%	Fair
den Hollander and Gouttebarge [71]	78%	Good	Robinson [101]	47%	Poor
Dorminy et al. [87]	55%	Fair	Sandmo et al. [96]	63%	Fair
Filben et al. [72]	83%	Good	Saunders et al. [48]	68%	Fair
Filben et al. [73]	72%	Good	Self et al. [97]	53%	Fair
Filben et al. [45]	78%	Good	Segars et al. [49]	67%	Fair
Filben et al. [88]	67%	Fair	Shewchenko et al. [80]	76%	Good
Hanlon and Bir [89]	56%	Fair	Shewchenko et al. [98]	59%	Fair
Harriss et al. [90]	67%	Fair	Sokol-Randell et al. [81]	78%	Good
Kalichova and Lukasek [91]	55%	Fair	Stelzer-Hiller et al. [102]	39%	Poor
Kenny et al. [74]	72%	Good	Stucker [82]	88%	Good
Lamond et al. [75]	83%	Good	Tierney et al. [83]	74%	Good
Larson [92]	68%	Fair	Tomblin et al. [84]	88%	Good
Liberi [93]	53%	Fair	Wang [99]	60%	Fair
Lukasek and Kalichova [94]	68%	Fair	Withnall et al. [100]	67%	Fair
Miller et al. [76]	83%	Good

The seven cross-sectional studies assessed using the AXIS tool showed varying levels of quality. In total, two [61, 62] were scored as ‘excellent’ quality, four [63–66] were ‘good’ quality, and one [67] was ‘fair’ quality. The two most common questions answered as ‘no’ or ‘not reported’ related to the justification of sample size (only completed in one cross-sectional study [61]) and the selection of participants being representative of the target population. Most (*n* = 41, 68%) studies were assessed using the modified Downs and Black tool, with results ranging from 39 to 90%. In total, 19 studies (46%) were ‘good’ quality [44, 45, 68–84], 20 studies (49%) were ‘fair’ quality [46–49, 85–100] and two studies (5%) were ‘poor’ quality [101, 102]. The accuracy of the measurement of head acceleration, the representativeness of the sample, the reporting of potential adverse events and the presence of sufficient power to detect relationships were items that were commonly scored as ‘no’ on this tool.

### Study Characteristics/Methods of Included Studies

**Fig. 5:**
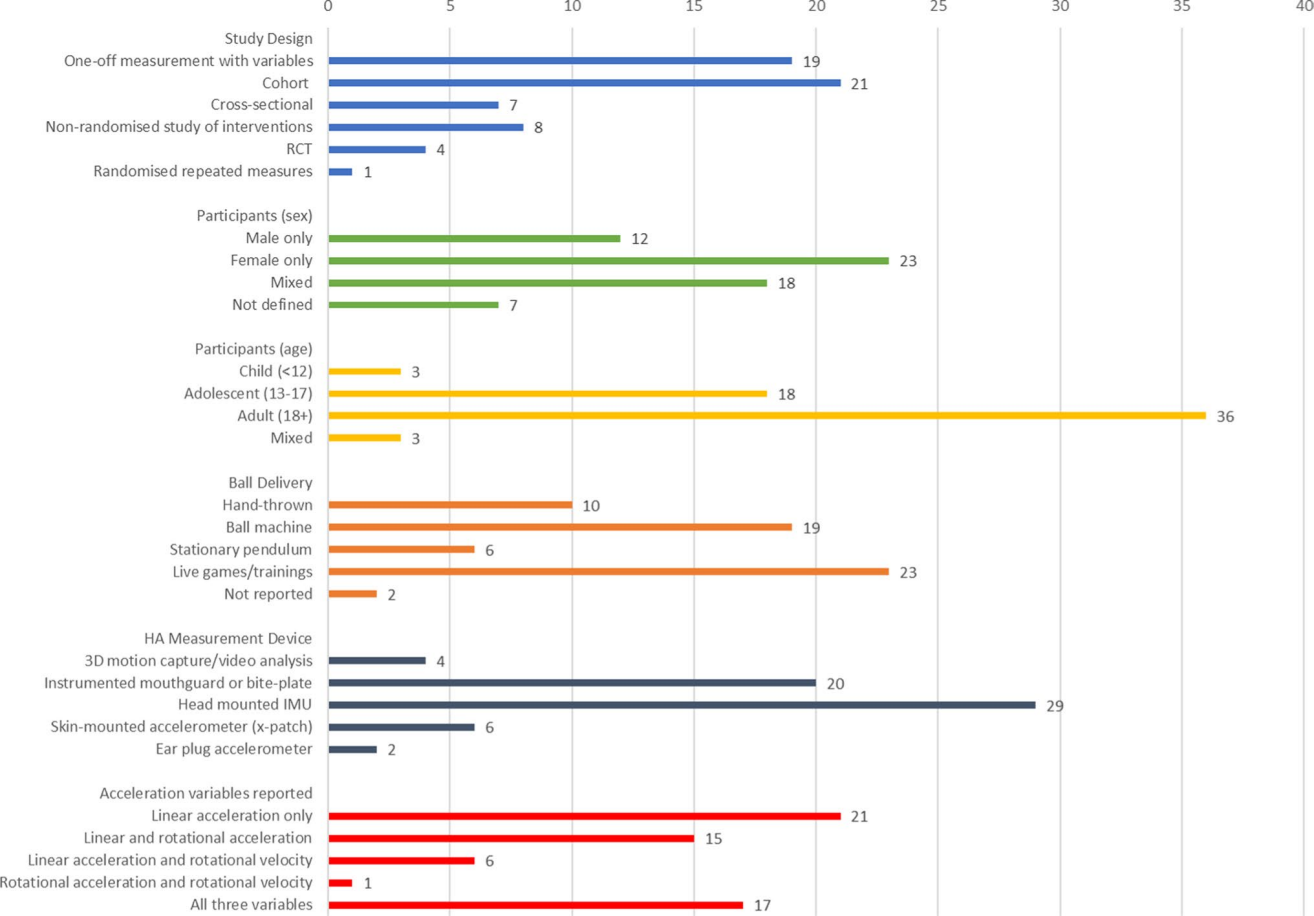
Study design, participants and methods. The figure represents the number of studies for each variable. one study used combined instrumented mouthguard and head mounted IMU; therefore, the HA measurement device section of this graph sums to 61 rather than 60 as all other sections. *HA* head acceleration, *IMU* inertial measurement unit

The 60 studies had various study designs, participant demographics and data collection methods (Fig. 5, Appendix V). The methods of delivering heading trials and measuring head acceleration varied. The most common methods of ball delivery were live games/trainings (35%), ball launching devices (31%) and hand-thrown balls (19%). Head acceleration was mostly measured using a head mounted inertial measurement unit (IMU), usually consisting of a tri-axial accelerometer with or without a gyroscope (52%). Following this, 27% of studies used an instrumented mouthguard (iMG) or biteplate, usually consisting of a tri-axial accelerometer with or without a gyroscope, with one paper using a combination of a head mounted IMU and an iMG. The remaining studies used a skin-mounted accelerometer over the mastoid process, three-dimensional (3D) motion capture/video analysis or an ear-plug accelerometer. Of the 60 studies included, only 18 studies transformed accelerometer data to a frame of reference (mainly centre of gravity of the skull), which is necessary to standardise data to account for different locations of accelerometer devices [104]. Finally, included studies reported varying measures of head acceleration from three possible variables (LA, RA/AA and RV/AV). In total, 20 studies reported LA only, 16 studies reported LA and RA/AA, 5 studies reported LA and RV/AV and 11 studies reported all three variables.

### Factors Affecting Head Acceleration

The results are grouped in categories of factors affecting head acceleration. Tables 6, 7, 8, 9, 10, 11, 12 review the results of each study. The narrative review accompanies this to outline key findings, agreement in the literature and where conflicting evidence is present. Several studies are included in more than one table where the study investigated more than one independent variable. Of note, no studies were identified that investigated sensorimotor factors such as cervical spine proprioception, oculomotor control and vestibular function and their relationship to head acceleration when performing a header.

#### Ball Characteristics and Speed

In total, 16 studies investigated the effect of ball speed and ball characteristics (Table 6) [52, 63, 78–80, 85–87, 91, 93–95, 97–100]. Agreement was seen across studies that head acceleration increased with increasing ball speed/velocity, with only 2 [80, 97] of the 12 studies rejecting this trend. Across the 12 studies that investigated the effect of ball speed, 6 [80, 85–87, 91, 97] undertook statistical analysis to investigate the relationship between ball speeds/velocities and resulting head acceleration (LA and/or RA/AA and/or RV/AA), while the remaining 6 [52, 63, 94, 95, 99, 100] reported raw acceleration data. Of studies where this relationship was investigated (*n* = 6), two found that with increasing ball speed, there was a statistical trend [87] and a statistically significant relationship with increased head acceleration [91]. One study found that greater pass distance (ball velocity) resulted in significantly higher LA, AA and AV [86]. A total of two studies had contrasting results [80, 97], with one finding no significant difference between low- and high-speed clearing headers (defensive header to clear the ball) [80]. Another compared two different ball speeds using two different header types; therefore, the effect of ball speed could not accurately be compared [97]. One study found increasing ball velocity had a significant effect on increasing AA, but the difference for LA did not reach significance [85]. Where studies did not undertake statistical analysis, our calculated MD and 95% CIs showed varying results. One study had a single participant, which meant MD and 95% CIs were not relevant [100]. One study found that head acceleration was significantly increased with increased ball speed [94]. However, in three studies [52, 63, 95], the increasing head acceleration was significant for LA only, with the calculated 95% CI for RA/AA crossing zero in all three studies. One further study found a significant increase in LA and AV, but no significant difference in AA [99].

**Table 6:** Results for the effect of ball speed and ball characteristics

Study	Design/participants	Independent variable	Head acceleration measures	Results	Conclusion
Austin et al. [85]	Controlled laboratory study—one-off measurement 12 male participants Age: 24 ± 3.2 years	Ball velocity 5 m/s versus 8 m/s Ball inflation pressure 6 psi versus 8 psi	Peak linear acceleration (g) Peak angular acceleration (rad/s^2^)	There was no interaction effect (*p* = 0.38) or main effect for velocity (*p* = 0.229) or pressure (*p* = 0.185) for LA For AA, there was no interaction effect (*p* = 0.595) or main pressure effect (*p* = 0.168), but there was a velocity effect (*p* < 0.001). High velocity headers showed an increase of 180 rad/s^2^ (95% CI: 104–255 rad/s^2^) over low velocity headers (*p* < 0.001)	• Limited effect on LA. This could potentially be due to the low accelerations reported • There was significantly different AA in both low- and high-pressure balls, with the high ball speed condition suggesting increased AA with increasing ball speed
Barnes-Wood et al. [86]	Controlled laboratory study—one-off measurement Seven male amateur soccer players Age: 20.1 ± 1 years	Ball velocity/length of ball delivery: 8 m/s short 13 m/s medium 16 m/s long	Peak linear acceleration (g) Peak angular acceleration (rad/s^2^) Peak angular velocity (rad/s)	Short pass LA: 18.72 ± 3.73 AA: 1242.30 ± 342.40 AV: 5.50 ± 1.27 Medium pass LA: 26.57 ± 5.51 AA: 1765.10 ± 369.90 AV: 6.77 ± 1.69 Long pass LA: 31.90 ± 7.28 AA: 2127.40 ± 663.95 AV: 9.05 ± 1.75 *Calculated mean difference (95% CI) Long versus short LA: 13.18 (9.55, 16.81); AA: 885.10 (553.55, 1216.65); AV: 3.55 (2.60, 4.50) Long versus medium LA: 5.33 (1.40, 9.26); AA: 362.30 (32.55, 629.05); AV: 2.28 (1.24, 3.32)	• Significant difference in LA, AA and AV between short and long passes and medium and long passes • Greater pass distance and increased ball velocity increases the acceleration of the head
Bretzin et al. [63]	Cross-sectional 13 NCAA Division I soccer players (8 F, 5 M)	Ball speed 25 mph versus 40 mph	Linear head acceleration (m/s) Rotational head velocity (rad/s^2^)	25 mph LA (m/s): 16.64 ± 3.22 RV (rad/s^2^): 891.85 ± 311.77 40 mph LA (m/s): 22.35 ± 5.25 RV (rad/s^2^): 1169.39 ± 582.71 *Calculated mean difference (95% CI) LA: 5.71 (2.18, 9.24) RV: 277.54 (− 100.76, 655.84)	• Greater LA and RV experienced with higher ball speed, although this is only significant for LA
Dorminy et al. [87]	Pre-test post-test design 16 collegiate soccer players (6 F, 10 M) Age: 20.44 ± 0.24 years	Ball speed 30, 40 or 50 mph	Linear head acceleration (g)	Ball speed = mean head acceleration (95% CI) 30 mph = 34.7 g (26.8–42.6) 40 mph = 49.2 g (39.0–59.4) 50 mph = 50.7 g (40.5–61.0) Post hoc *t*-tests 30 versus 40 mph, *p* = 0.097 30 versus 50 mph, *p* = 0.063 40 versus 50 mph, *p* = 1.000	• Significant increase in acceleration values between the 40 and 50 mph groups versus the 30-mph group • As ball speed increases, so does the magnitude of force applied to the head
Kalichova and Lukasek [91]	Controlled laboratory study—one-off measurement 63 footballers of four age categories U11–U17 16 U11 players with average age: 10.3 years 15 U13 footballers with average age: 12.4 years 18 U15 players with average age: 14.4 years 14 U17 players with average age: 16.3 years	Ball drop height 0.5 m, 1.0 m and 1.5 m from the players head	Linear acceleration (g)	A statistically significant relationship was established in all cases between maximum head acceleration and ball impact speed For the relationship between a_max0.5 m and a_max1.0 m (*p* < 0.05, *r* = .76), for a_max1.0 m and a_max1.5 m *r* = .78, for a_max0.5 m and a_max1.5 m *r* = .70 To compare ball speed alone data from just the U17 group analysed below 0.5 m: − 33.8 ± 13.3 1.0 m: − 53.3 ± 21.6 1.5 m: − 78.3 ± 24.8 *Calculated mean difference (95% CI) 1.5 m versus 1.0 m: − 25.00 (− 43.07, − 6.93) 1.5 m versus 0.5 m: − 44.50 (− 59.96, − 29.04)	• Negative head acceleration increased with increasing ball drop height and with increasing speed of the ball hitting the head
Liberi [93]	Controlled laboratory study – one-off measurement 16 collegiate male varsity soccer players (Division I) Age: 19.5 years	Wet ball versus dry ball	Linear head acceleration (g)	Dry ball: 22.34 ± 2.56 g Wet ball: 20.81 ± 3.07 g A one way ANOVA with repeated measures produced a statistically significant difference between the two conditions for the measure of head acceleration *Calculated mean difference (95% CI) LA: 1.53 (− 0.51, 3.57)	• No significant difference between the wet and dry ball conditions
Lukasek and Kalichova [94]	Controlled laboratory study – one-off measurement 16 male youth footballers (U11) Age: 10 years	Ball drop height/ball velocity	Head acceleration	Ball velocity 3.13 m/s: − 59.4 ± 15.8 m/s^2^ Ball velocity 4.43 m/s: − 76.4 ± 15 m/s^2^ Ball velocity 5.43 m/s: − 100.1 ± 25 m/s^2^ *Calculated mean difference (95% CI) Between 3.13 m/s and 4.43 m/s: − 17.00 (− 28.12, − 5.88) Between 3.13 m/s and 5.43 m/s: − 40.70 (− 55.80, − 25.60)	• Average head acceleration increased with increasing ball velocity
Naunheim et al. [95]	Controlled laboratory study – one-off measurement Four male subjects Age: 25–36 years	Ball speed 9 m/s versus 12 m/s	Linear head acceleration (m/s^2^) Angular head acceleration (rad/s^2^)	9 m/s = 158 ± 19 m/s^2^ and 1302 ± 324 rad/s^2^ 12 m/s = 199 ± 27 m/s^2^ and 1457 ± 297 rad/s^2^ *Calculated mean difference (95% CI) LA: 41 (0.61, 81.39) AA: 155 (− 382.74, 692.74)	• Significantly greater LA with increased ball speed • No significant difference in AA between ball speeds
Peek et al. [78]	Controlled laboratory study – one-off measurement 61 youth soccer players (26 F, 35 M) Age: 14.52 ± 1.37 years	Ball characteristics – inflation pressure, mass and characteristics 1. *KickerBall*, size 5, 192 g and 5.00 psi 2. *Adidas Starlancer*, size 5, 432 g and 5.00 psi 3. *Heading-pro*, size 4, 255 g and 5.00 psi 4. *Deploy Envision*, size 5, 430 g and 10.5 psi	Peak linear head acceleration (g) Peak angular velocity (dps)	Statistically significant difference found between ball type and the dependent variables (*p* < 0.001). Statistically significant differences for LA (adjusted R2 = 0.68; *p* < 0.001) and AV (adjusted R2 = 0.28; *p* < 0.001). The result of the one-way MANOVA by sex demonstrated statistically significant differences between ball type and both LA and AV for males (*p* < 0.001) and females (*p* < 0.001) for Balls 1–3. The mean LA of the players’ head during heading was 47% lower for Ball 3 when compared with Ball 4. The difference between means (using first Ball 4 as the reference ball followed by Ball 2), were statistically significant for all comparison balls, regardless of which ball was used as the reference For linear head acceleration, 3% of the variance was predicted by age (older players demonstrating lower PLA), 11% by sex (females demonstrating lower PLA), 38% by ball pressure (lower ball pressures associated with lower PLA) and 56% by ball mass (lighter balls associated with lower PLA). For AV, 11% of the variance was predicted by age (older age being associated with lower AV), 23% by sex (females demonstrating lower AV), 10% by the number of headers performed per week (players who headed the ball more often had lower AV), 24% by ball pressure (lower ball pressures were associated with lower AV) and 13% by ball mass (lighter balls were associated with lower AV)	• Head accelerations for youth players can be reduced by 59% using a lighter size 5 ball (Ball 1), 47% using a lighter size 4 ball (Ball 3) and 26% using a size 5 ball with reduced ball pressure (Ball 2) when compared with a size 5 match-regulated ball (430 g; 10.5 psi) • Lower head accelerations were also observed using a size 4 ball (5 psi) when compared with both a low pressure (5 psi) size 5 ball and a higher pressure (10.5 psi) size 5 match-regulated ball • Multiple regressions supported that lighter balls or balls with lower pressures were associated with lower PLA and PAV • Although older age and female sex were associated with lower head accelerations during heading, there was a stronger relationship between lower head acceleration and lower ball mass and pressure
Pereira et al. [79]	Randomised repeated measures 17 recreational adult football players (3 F, 14 M) Age: 22 ± 3.5 years	Low-pressure ball (58.6 kPa; 8.5 psi) versus high-pressure ball (103.4 kPa; 15.0 psi)	Peak linear acceleration (g) Peak angular velocity (rad/s)	Low pressure (mean (95% CI)) PLA: 12.8 (12–13.3) PAV: 15.3 (14.1–15.4) High pressure (mean (95% CI)) PLA: 14.4 (13.7–14.7) PAV: 16.2 (14.5–16) The result of the linear mixed effect model demonstrated a statistically significant difference between ball pressure and both peak linear acceleration (*F* = 15.2; *p* < 0.001) and peak angular velocity (*F* = 5.71; *p* = 0.018) An independent *t*-test revealed a statistically significant difference in PLA and PAV of the head between low- and high-pressure balls (*p* < 0.05). There was a 12% reduction in PLA and a 6% reduction in PAV when heading with a low-pressure ball compared with a high-pressure ball	• Head kinematics are influenced by ball pressure • Significant reduction in PLA and PAV with reduced ball pressure
Self et al. [97]	Controlled laboratory study – one-off measurement Ten participants from the US Air Force Academy Men’s Varsity Soccer Team	Ball speed 16.5 m/s versus 12.2 m/s	Linear head acceleration (g)	Average acceleration Straight: 16.5 m/s: 32.64 g Corner: 12.2 m/s: 29.26 g No significant difference between the resultant maximum accelerations between the corner and straight headers (*p* = 0.29)	• No significant difference in HA between corner headers (12.2 m/s) and straight headers (16.5 m/s)
Shewchenko et al. [80]	Controlled laboratory study – one-off measurement Seven non-professional soccer players aged 20–23 years	Ball speed 6 m/s versus 8 m/s	Peak linear acceleration (m/s^2^) Peak angular acceleration (krad/s^2^)	Many different heading scenarios tested. For comparison of ball speeds the low speed (6 m/s) clearing header and the high speed (8 m/s) clearing header are compared PLA (m/s^2^) Low speed: 169 ± 23 High speed: 158 ± 16 Peak AA (krad/s^2^) Low speed: 1.52 ± 0.13 High speed: 1.46 ± 0.36 *Calculated mean difference (95% CI) LA: − 11 (− 34.07, 12.07) AA: − 0.06 (− 0.38, 0.26)	• No significant difference between the low speed (6 m/s) and high speed (8 m/s) clearing headers
Shewchenko et al. [98]	Controlled laboratory study – one-off measurement Three non-professional soccer players aged 20–23 years	Ball characteristics – inflation pressure, mass and characteristics Balls tested: 1. Baseline, 444 g, 0.8 bar, *Fevernova Tri-Lance* 2. Low pressure 0.6 bar, *Fevernova Tri-Lance* 3. High pressure 1.1 bar, *Fevernova Tri-Lance* 4. Low mass 299 g, *Fevernova Junior 290* 5. Low mass 351 g, *Fevernova Junior 350*	Peak linear acceleration (m/s^2^) Peak angular acceleration (krad/s^2^)	PLA (m/s^2^) Ball 1: 156 ± 2 Ball 2: 141 ± 21 Ball 3: 175 ± 38 Ball 4: 142 ± 17 Ball 5: 140 ± 21 PAA (krad/s^2^) Ball 1: 1.47 ± 0.27 Ball 2: 1.60 ± 0.65 Ball 3: 1.61 ± 0.84 Ball 4: 1.47 ± 0.03 Ball 5: 1.23 ± 0.31 Decreases in PLA up to 10% were observed for a ball pressure decrease of 50%. Overall decrease in LA response with an increase in the contact duration. The AA were increased in all cases of change to the ball pressures and may be due to the associated variability with AA measurements	• A reduction in ball mass and pressure results in improvements to both the PLAs and power index • The magnitudes of these changes are equal or greater to those observed with changes in heading technique
Wang [99]	Controlled laboratory study – one-off measurement Eight participants (two F, six M) Age: 18–30 years	Ball speed Two impact levels (low-level and high-level)	Linear acceleration (g) Angular acceleration (rad/s^2^) Angular velocity (rad/s)	Low level impacts: Mean LA: 7 ± 1.4 g AV: 4.88 ± 1.27 rad/s AA of 928 ± 465 rad/s^2^ High level impacts: Mean LA: 10 ± 1.9 g AV of 8.29 ± 1.87 rad/s AA of 1331 ± 938 rad/s^2^ *Calculated mean difference (95% CI) LA: 3.00 (1.21, 4.79) AA: 403 (− 390.89, 1196.89) AV: 3.41 (1.70, 5.12)	• Linear and angular head acceleration along with AV were increased in the high-level impact condition. Significance met for LA and AV but no significant difference in AA between ball speeds
Waring et al. [52]	Randomised control trial 20 varsity collegiate soccer players (12 F, 8 M) Age: 20.15 ± 1.35 years	Ball speed 11.18 m/s versus 17.88 m/s	Peak linear acceleration (g) Peak rotational acceleration (deg/s^2^)	For ball speed analysis, pre-intervention data (mean and SD) were pooled across control and intervention groups 11.18 m/s PLA: 22.4 ± 2.39 g PRA: 4742.02 ± 884.47 deg/s^2^ 17.88 m/s PLA: 28.04 ± 4.32 g PRA: 4559.27 ± 840.23 deg/s^2^ *Calculated mean difference (95% CI) LA: 5.64 (2.36, 8.92) RA: − 182.75 (− 993.25, 627.75)	• Significant difference in LA between ball speeds, with faster ball speed resulting in increased acceleration • No significant difference in RA between the two ball speeds
Withnall et al. [100]	Controlled laboratory study – one-off measurement One participant (M) Age: 30 years	Ball speed 6.4 m/s versus 8.2 m/s	Linear acceleration (g)	Results for bare head trial presented here to compare ball speeds LA: 6.4 m/s = 5.4 g ± 1.5 g 8.2 m/s = 7.6 g ± 1.6 g	• Raw data shows increasing LA with increasing ball speed, no statistical analysis possible on this outcome to confirm the significance of this result

Italicised text indicates ball models/brand names

*AA* angular acceleration, *CI* confidence interval, *degree/s* degree per second, *dps* degrees per second, *g* gravitational acceleration, *HA* head acceleration, *kPa* kilopascal, *krad****/****s* kiloradian per second, *LA* linear acceleration, *m* metre, *m****/****s* metres per second, *mph* miles per hour, *PAA* peak angular acceleration, *PLA* peak linear acceleration, *PRA* peak rotational acceleration, *PRV* peak rotational velocity, *psi* pounds per square inch, *RA* rotational acceleration, *rad****/****s* radian per second, *RV* rotational velocity

^*^Indicates where mean difference and 95% CI have been calculated by the authors of this review on the basis of raw data provided in the included studies

A total of three studies found that heading balls with reduced mass or heading balls with lower pressure resulted in lower head accelerations [78, 79, 98]. The relationship between ball characteristics and head acceleration was stronger than the effect of age or sex [78], and the magnitude of change in head kinematics was equal to or greater than the changes observed with heading technique [98]. Increased ball pressure showed significant increases in peak linear acceleration (PLA) and peak angular velocity (PAV) [79]. One study found no effect of inflation pressure on LA or RA; however, this study noted the low velocities tested and that the head impact kinematics may have been too low to be affected by ball characteristics [85]. One study comparing wet and dry soccer balls found that LA of the head was greater in the wet ball condition [93]. Although statistically significant in the authors’ analysis, the authors questioned the clinical significance [93], and our MD and 95% CI calculation demonstrated no significant difference between wet and dry balls.

#### Header Type and Head Impact Location

In total, 14 studies investigated the effect of the type of header (jumping, standing, running, clearing and flick-on) and/or the location of the head impact to the player’s head (Table 7) [45, 50, 58, 68, 74, 81, 84, 88–90, 92, 96, 97, 101]. Appendix VI contains definitions of each header type from the included papers.

**Table 7:** Results for the effect of header type and head impact location

Study	Design/participants	Independent variable	Head acceleration measures	Results	Conclusion
Becker et al. [68]	Controlled laboratory study – one-off measurement 60 male active soccer players Age: 18.9 ± 4.0 years	Header type: standing, jumping, running	Linear head acceleration (g)	LA (g) Standing header: 6.41 ± 1.31 Jumping header: 5.21 ± 1.64 Running header: 6.80 ± 1.01 *Calculated mean difference (95% CI) Standing versus jumping: 1.20 (0.66, 1.74) Running versus jumping: 1.59 (1.10, 2.08) Running versus standing: 0.39 (− 0.03, 0.81)	• Running and standing headers had greater acceleration than jumping headers • No difference between running and standing headers
Becker et al. [58]	Non-randomised study of interventions 68 soccer players, fourth through ninth division, and active recreational players Age: 21.5 ± 3.8 years	Header type: standing, jumping, running	Linear head acceleration (g)	Pre-intervention data used for analysis LA (g) Standing: 6.0 ± 1.1 Jumping: 5.6 ± 1.1 Running: 7.3 ± 0.9 The post hoc Scheffé test showed significant differences between the standing and running variants (*p* = 0.000; *d* = 1.29) and between the jumping and running variants (*p* = 0.000, *d* = 1.69) *Calculated mean difference (95% CI) Standing versus jumping: 0.40 (0.03, 0.77) Running versus jumping: 1.70 (1.35, 2.05) Running versus standing: 1.30 (0.95, 1.65)	• Running header had greater head acceleration than standing and jumping headers • Standing headers had greater head acceleration than during jumping
Filben et al. [45]	Cohort Female NCAA Division I soccer players, 24 player season (from 14 unique players) Age: 19.8 ± 1.24 years	Head impact location Header type (standing versus jumping)	Linear head acceleration (g) Rotational head acceleration (krad/s^2^) Rotational velocity (rad/s)	*Impact location* Top of head: LA, g: 17.1 (14.2–20.7) RA, krad/s^2^: 1.77 (1.51–2.08) RV, rad/s: 8.10 (7.24–9.06) Side of head: LA, g: 17.7 (12.5–25.1) RA, krad/s^2^: 1.79 (1.19–2.69) RV, rad/s: 9.03 (6.95–11.7) Forehead: LA, g: 18.8 (15.4–22.9) RA, krad/s^2^: 1.75 (1.46–2.09) RV, rad/s: 7.66 (6.77–8.67) No significant difference in mean peak kinematics between jumping headers and standing headers	• No significant difference in mean peak kinematics between jumping headers and standing headers • No differences in mean peak kinematics were observed by header impact location
Filben et al. [88]	Cohort 14 female soccer players Age: 14.4 ± 0.9 years	Header type: jumping versus standing	Peak linear acceleration (g) Peak rotational acceleration (rad/s^2^) Peak rotational velocity (rad/s)	Jumping headers (*n* = 77): PLA: 15.4 ± 10.1 PRA: 1460 ± 1160 PRV: 7.1 ± 3.3 Standing headers (*n* = 212): PLA: 9.6 ± 5.5 PRA: 685 ± 721 PRV: 4.7 ± 2.5 *Calculated mean difference (95% CI) PLA: 5.8 (3.96, 7.64) PRA: 775 (549.94, 1000.06) PRV: 2.4 (1.68, 3.12)	• Jumping headers were generally higher magnitude than standing headers. This was found to be significant across all three acceleration variables with mean difference and 95% CI calculations
Hanlon and Bir [89]	Cohort 24 youth girls soccer players Age: U14 years	Location of header (left, right, top, front or back of head)	Linear head acceleration (g) Angular head acceleration (rad/s^2^)	LA (g) Back: 11.9 ± 5.9 Front: 17.4 ± 8.4 Top: 19.5 ± 10.7 Left side: 27.2 ± 14.4 Right side: 28.1 ± 20.8 **Pooled left and right sides: 27.86 ± 17.48 AA (rad/s^2^) Back: 723.2 ± 220.3 Front: 1657.5 ± 954.5 Top: 1851.8 ± 1061.7 Left side: 2586.6 ± 1501.5 Right side: 3003.4 ± 2823.9 **Pooled left and right sides: 2808.89 ± 2236.01 Calculations performed using front header as base variable and comparing to top and pooled sides data *Calculated mean difference (95% CI) Top versus front: LA: 2.1 (− 5.75, 9.95); AA: 194.3 (− 649.29, 1037.89) Side versus front: LA: 10.46 (0.75, 20.17); AA 1151.39 (− 63.33, 2366.11)	• The location of the head impact affects head acceleration, with side impacts having greater LA than back impacts and front and side impacts having greater AA than back impacts • As it is not common to perform a header with the back of your head, mean difference and 95% CIs were calculated between more likely impact locations. No significant differences between the front of the head versus the top of the head. With the data for the left and right sides pooled versus front of head there was no difference in AA; however, side impacts had significantly greater LA than front impacts
Harriss et al. [90]	Cohort 36 female elite youth soccer players Age: 13.4 ± 0.9 years	Head impact location	Peak linear acceleration (g) Peak rotational velocity (degrees/s)	LA (g) Front: 18.35 ± 8.50 Top: 19.69 ± 12.33 Side: 19.41 ± 14.89 RV (degrees/s) Front: 951.88 ± 550.52 Top: 1215.44 ± 558.56 Side: 1037.66 ± 499.62 *Calculated mean difference (95% CI) Top versus front of head LA: 1.34 (− 3.64, 6.32) RV: 263.56 (2.87, 524.25) Side versus front of head LA: 1.06 (− 4.64, 6.76) RV: 85.78 (− 161.34, 332.90)	• Head impact location did not significantly influence LA • RV varied significantly between headers performed with the top of the head and those performed with the front of the head • There was no statistically significant difference in RV between purposeful headers that occurred at the side of the head compared with the front of the head
Huber et al. [50]	Randomised control trial 19 soccer players (2 F, 17 M) Age: 15.7 ± 0.4 years	Type of header: frontal versus oblique headers	Peak linear acceleration (g) Peak angular velocity (rad/s) Peak angular acceleration (rad/s^2^)	Frontal headers resulted in higher mean PLA (17.4 ± 0.5 g) compared with oblique headers (12.1 ± 0.4 g, *p* < 0.001), and oblique headers resulted in higher PAV (frontal: 5.6 ± 0.2 rad/s, oblique: 10.1 ± 0.4 rad/s^1^, *p* < 0.001) and AA (frontal: 1147 ± 45 rad/s^2^, oblique: 1410 ± 65 rad/s^2^, *p* < 0.001)	• Frontal headers resulted in higher PLA than oblique headers • Oblique headers had 80% and 21% higher PAV and PAA, respectively, than frontal headers
Kenny et al. [74]	Cohort 13 female university varsity soccer players Age: 19.9 ± 1.6 years	Type of header Location of head impact	Peak linear acceleration (g) Peak angular acceleration (rad/s^2^) Peak angular velocity (rad/s)	Top of the head impacts (12 g; 1125 rad/s^2^) were significantly higher than forehead impacts (10.8 g; 858 rad/s^2^) in all mean peak kinematics (*p* < 0.001) Jumping head impacts (14.1 g; 1291 rad/s^2^) were significantly higher than non-jumping head impacts (10.7 g; 903.2 rad/s^2^) in mean peak kinematics (*p* < 0.001)	• These results imply that headers performed with the forehead may lead to lower impact kinematics, while top or side impacts may lead to higher impact kinematics
Larson [92]	Controlled laboratory study – one-off measurement 13 Division II soccer athletes (7 F, 6 M) Age: 18–22 years	Type of header High clearing versus driven versus flick-on	Linear acceleration (g)	To compare header types only, male and female data have been pooled for this analysis LA (g) High-clear header: 13.79 ± 1.24 Driven header: 13.25 ± 1.53 Flick-on header: 14.91 ± 2.47 *Calculated mean difference (95% CI) Flick-on versus high-clear: 1.12 (− 0.46, 2.70) Flick-on versus driven: 1.66 (0.00, 3.32) High-clear versus driven: 0.54 (− 0.59, 1.67)	• Pooled data between males and females show significance was just reached when comparing flick-on to driven headers, with flick-on headers having greater accelerations • No difference between driven or high-clear type of headers
Robinson [101]	Mixed laboratory-based and live game play data collection 14 varsity women’s soccer players	Type of header – passing versus clearing versus shooting and stationary versus running	Peak linear acceleration (g) Peak rotational acceleration (degree/s^2^) Peak rotational velocity (degree/s)	Most maximum values come from stationary headers. Particularly, the 20-yard stationary passing header tends to have the maximum PLA, PRA and PRV values, occurring as the maximum for at least 50% of the players In comparing the 10-yard stationary shooting header with the 10-yard running shooting header, it is seen that Players 1, 4, 5, 10 and 13 had higher PRA, PLA and PRV values for the running type than the stationary type. Players 2 and 9 had lower PRA, PLA and PRV values for the running type than the stationary type. Players 6, 7, 8, 11 and 12 had some mix of the two previous cases, with PRA, PLA and PRV not being constantly higher or lower in either stationary or running	• Limited information to be taken away from this paper owing to the lack of statistical analysis, the variability of results and the number of variables measured in a small sample size • The max PLA, PRA and PRV values for the 20-yard stationary passing header could be due more in part to the distance of the incoming header as opposed to the header type or stationary versus running classification
Sandmo et al. [96]	Controlled laboratory study – one-off measurement 6 male youth soccer players Age: 15.3 ± 0.3 years	Type of header	Peak linear acceleration (g) Peak rotational acceleration (rad/s^2^) Peak rotational velocity (rad/s)	Finishing headers had higher PLA and PRA than any of the other header types. This was followed by redirectional headers, with direct headers having lower PLA and PRA No numerical data given for each header type – box and whisker plots used to display results, unable to calculate mean difference and 95% CI	• No individual data provided for each header type and no statistical analysis done to compare between header type. With review of the box and whisker plots, it appears that finishing headers had the greatest PLA and PRA
Self et al. [97]	Controlled laboratory study – one-off measurement Ten participants from the US Air Force Academy Men’s Varsity Soccer Team	Header type Straight versus redirecting/corner	Linear head acceleration (g)	LA (g) Straight header: 32.64 Redirecting/corner header: 29.26 There was no significant difference between the resultant maximum accelerations between the comer and straight headers (*p* = 0.29)	• There was no significant difference between the resultant maximum accelerations between the comer and straight headers
Sokol-Randell et al. [81]	Cohort Ten male university soccer players Age: 20 ± 1 years	Header type	Peak linear acceleration (g) Peak angular acceleration (krad/s^2^)	For the header types (clearance flick-on, headed control, interception, pass and shot), there were no significant differences in kinematics Shot attempts had the highest median PLA and PAA	• No significant variations in head kinematics among differing header types
Tomblin et al. [84]	Cohort 14 youth female soccer athletes Age: 12–15 years	Type of header – standing versus jumping	Peak linear acceleration (g) Peak rotational velocity (rad/s) Peak rotational acceleration (rad/s^2^)	Standing headers (*n* = 257) PLA (g) Range: 4.4–56.1; 50th percentile: 8.3; 95th percentile: 22.5 PRA (rad/s^2^) Range: 225.1–5219.8; 50th percentile: 496.2; 95th percentile: 2775.2 PRV (rad/s) Range: 1.6–18.7; 50th percentile: 4.2; 95th percentile: 10.8 Jumping headers (*n* = 103) PLA (g) Range: 1.8–48.0; 50th percentile: 12.0; 95th percentile: 35.3 PRA (rad/s^2^) Range: 52.8–6016.3; 50th percentile: 1142.0; 95th percentile: 3936.8 PRV (rad/s) Range: 1.2–25.3; 50th percentile: 6.7; 95th percentile: 13.2	• The 95th percentile LA, RV and RA for jumping headers were greater than for standing headers

*AA* angular acceleration, *CI* confidence interval, *degree/s or dps* degree per second, *g* gravitational acceleration, *HA* head acceleration, *krad/s* kiloradian per second, *LA* linear acceleration, *m/s* metres per second, *mph* miles per hour, *PAA* peak angular acceleration, *PLA* peak linear acceleration, *PRA* peak rotational acceleration, *PRV* peak rotational velocity, *RA* rotational acceleration, *rad/s* radian per second, *RV* rotational velocity

^*^Indicates where mean difference and 95% CI have been calculated by the authors of this review on the basis of raw data provided in the included studies

^**^Indicates where data was pooled to allow mean difference and 95% CIs to be calculated

Results were mixed for the type of header and the effect on head acceleration. A total of two studies using a pendulum device for ball delivery found that running headers had the greatest head acceleration, followed by standing headers, then jumping headers [58, 68]. In contrast, two studies measuring headers during ‘live’ games or trainings found that jumping head impacts were significantly higher than non-jumping impacts (*p* < 0.001) [74], and 50th and 95th percentile LA and RA were both greater in jumping headers than in standing headers [84]. Filben et al. [88], found that jumping headers had greater PLA, peak rotational acceleration (PRA) and peak rotational velocity (PRV) when compared with standing headers. In total, two further studies found no difference in head kinematics between standing and jumping headers [45] and mixed results between running and standing headers for LA and RA measures [101]. Regarding studies investigating the type of header, one study found that finishing headers had higher PLA and PRA than redirectional and direct headers; however, no statistical analysis was performed by those authors, and it was not possible, as acceleration data were not provided [96]. When pooling the data of males and females for each header type, significance was met when comparing flick-on with driven headers (flick-on header with greater LA), with no difference between the other two header types [92]. Another study found no difference between straight and redirectional headers [97], and Sokol-Randell et al. [81] found no difference in PLA or peak angular acceleration (PAA) between six different header types. When comparing frontal and oblique headers, frontal headers had greater PLA while oblique headers had greater AA and AV [50].

There was some agreement in relation to the location of the head impact and resulting head acceleration. Headers performed with the top of the head had significantly greater LA and AA (*p* < 0.001) [74] and significantly greater RV [90] than headers performed with the front of the head/forehead. Comparing a wider variety of impact locations, it was found that side-of-the-head impacts had greater LA than impacts to the back of the head, and front- and side-of-the-head impacts had greater AA than back-of-head impacts [89]. As headers are not often performed with the back of the head, further calculations were made between top-, front- and side-of-head headers, with no significant differences in LA or AA between front- and top-of-head impacts [89]. However, a significant difference in LA (not significant for AA) was found between side-of-the-head and front-of-the-head impacts, with side impacts having greater acceleration [89]. One further study found no difference in mean peak kinematics between top-of-head, side-of-head or forehead impacts [45].

#### Fatigue Protocols

In total, four studies investigated the effect of fatigue protocols, with the hypothesis that fatigue would increase head acceleration (Table 8) [53, 56–58]. A total of three of these studies used core and trunk muscle fatigue protocols, utilising the Bourban test (a trunk muscle strength test), with additional exercises performed until complete exhaustion [56–58]. Contrary to their hypothesis, two of these studies [57, 58] showed a slight reduction in head acceleration following the fatigue intervention, while the third study [56] found no changes in head acceleration with fatigue. One study used a more generalised fatigue intervention with cardiovascular and strength-based exercises (more specific to expected game fatigue from playing soccer), noting increased LA and RA following the intervention, reaching significance for LA only [53].

**Table 8:** Results for the effect of fatigue protocols

**Study**	Design/Participants	Independent Variable	Head acceleration measures	Results	Conclusion
Becker et al. [56]	Non-randomised study of interventions 33 male soccer players Age: 20.3 ± 3.6 years Two intervention groups IG1 – adult team (*n* = 11) IG2 – youth team (*n* = 9) Control group: CG (*n* = 13)	Intervention: Trunk fatigue protocol. Fatigue of the trunk muscles was achieved by performing the Bourban test after the data acquisition of standing, jumping and running headers. The Bourban test was extended by two exercises (static hyperextension, sling: plank crunch) to ensure fatigue	Head acceleration (g)	Results pooled across the three groups (IG1, IG2 and CG). Measured as LA (g) Pre-fatigue jumping: 5.93 ± 1.06 Post-fatigue jumping: 5.72 ± 1.54 Pre-fatigue running: 7.75 ± 1.37 Post-fatigue running: 7.39 ± 1.45 *Calculated mean difference (95% CI) Jumping headers: − 0.21 (− 0.86, 0.44) Running headers: − 0.36 (− 1.05, 0.33)	• No significant changes in acceleration after fatigue in either jumping or running headers
Becker et al. [57]	Non-randomised study of interventions 41 amateur soccer players divided into two independent subgroups 1. Muscular activity (*n* = 12) Age: 23.6 ± 4.2 years 2. Kinematics and dynamics (*n* = 29) Age: 23.7 ± 2.8 years	Intervention: Core fatigue protocol. To exhaust the core-stabilizing musculature, the subjects carried out three abdominal (dynamic leg raising, sit-ups with dynamic rolling off and static forearm push-ups) and two dorsal exercises (dynamic trunk extension and static trunk extension) up to the point of subjective complete fatigue. All exercises were performed in one set with a 1-min break in between them. The post-test was conducted 1 min after the treatment	Head acceleration (g)	Pre-fatigue: 2.7 ± 0.5 g Post-fatigue: 2.5 ± 0.5 g *Calculated mean difference (95% CI) − 0.20 (− 0.46, 0.06)	• No significant changes in head acceleration after core muscle fatigue
Becker et al. [58]	Non-randomised study of interventions 68 soccer players, fourth through ninth division, and active recreational players Age: 21.5 ± 3.8 years	Intervention: Core fatigue protocol. The core-stabilizing musculature was fatigued directly after the pre-test based on an extended version of the Bourban test 1. Plank with alternating leg lifts; 2. right-side plank with pelvis drop and lift; 3. left-side plank with pelvis drop and lift; 4. dynamic hyperextension; 5. static hyperextension; 6. sling: plank crunch All exercises were performed in one set to the point of subjective complete exhaustion. The effectiveness of the treatment was checked during a separate electromyographical examination. All exercises were performed in one set with a 1-min break in between them. The post-test was conducted 1 min after the fatigue treatment	Linear head acceleration (g)	Jump headers: Pre-fatigue: 5.6 ± 1.1 g Post-fatigue: 5.3 ± 1.1 g Run headers: Pre-fatigue: 7.4 ± 0.9 g Post-fatigue: 7.2 ± 1.0 g *Calculated mean difference (95% CI) Jump headers: − 0.30 (− 0.68, 0.08) Run headers: − 0.20 (− 0.57, 0.17)	• No changes in head acceleration following the core muscle fatigue intervention
Cheever et al. [53]	Non-randomised study of interventions 40 current female collegiate soccer athletes Age: 18–25 years	Intervention: The 20-min fatigue protocol consisted of seven stations: 5-min 60% jog, 3 min of sprints up and down basketball court, 2 min of push-ups, 2 min of sit-ups, 3 min of 12-inch step-ups, 3 min of sprints and a 2-min run (participants were instructed to maintain the fastest pace possible for 2 min). The post-test was completed immediately following the fatigue protocol. Participants assigned to the fatigue + heading group completed the fatigue intervention while performing one header every 5 min. The first header was completed prior to starting the fatigue protocol and the final at the conclusion of the fatigue protocol. The post-test immediately followed this protocol	Peak linear acceleration (g) Peak rotational acceleration (rad/s^2^)	Mean LA (g) Heading only: 13.90 ± 3.8 Heading + fatigue: 19.00 ± 5.4 Mean RA (rad/s^2^) Heading only: 1079.40 ± 312.5 Heading + fatigue: 1202.50 ± 416.4 Mean difference (95% CI) calculated* LA: 5.10 (0.71, 9.49) RA: 123.10 (− 153.57, 399.77)	• Increased LA and RA following the fatigue protocol. Only LA showed a significant increase

*AA* angular acceleration, *CG* control group, *CI* confidence interval, *g* gravitational acceleration, *HA* head acceleration, *IG* intervention group, *LA* linear acceleration, *PAA* peak angular acceleration, *PLA* peak linear acceleration, *PRA* peak rotational acceleration, *PRV* peak rotational velocity, *RA* rotational acceleration, *rad/s* radian per second, *RV* rotational velocity

^*^Indicates where mean difference and 95% CI have been calculated by the authors of this review on the basis of raw data provided in the included studies

#### Neck Strength, Anthropometrics and Demographics

In total, 18 studies investigated the effect of neck strength and/or various anthropometric and demographic measures on head acceleration (Table 9) [44, 46, 48, 59, 61–65, 67, 68, 71, 79, 83, 88, 91, 92, 101].

**Table 9:** Effect of strength, anthropometric and demographic factors

Study	Design/Participants	Independent Variable	Head acceleration measures	Results	Conclusion
Becker et al. [68]	Controlled laboratory study – one-off measurement 60 male active soccer players Age: 18.9 ± 4.0 years	Head–neck–torso alignment	Linear head acceleration (g)	No significant relationship between the three angles (cervical spine angle, head angle anad thoracic spine angle) affecting the head–neck–torso alignment and head acceleration was found for the standing, jumping, and running approach	• No relationship between head acceleration and head–neck–torso alignment
Bretzin et al. [63]	Cross-sectional 13 NCAA Division I soccer players (8 F, 5 M) Age: 19.80 ± 0.94 years	Sex Anthropometrics Neck strength	Linear head acceleration (g) Rotational head velocity (rad/s^2^)	Males 25mph // 40 mph LA (m/s): 14.70 ± 2.24 // 19.58 ± 5.10 RV (rad/s^2^): 656.56 ± 258.03 // 774.60 ± 501.13 Females 25mph // 40 mph LA (m/s): 17.86 ± 3.24 // 24.08 ± 4.85 RV (rad/s^2^): 1038.90 ± 253.63 // 1416.13 ± 507.63 Significant differences in muscle groups between male and female athletes. RV had a statistically significant difference between sexes at both speeds. Significant negative relationships were identified between neck girth and LA at 25 mph, RV at 25 mph, and RV at 40 mph. Neck girth and head–neck segment mass each had significant positive relationships with varying strength groups. Significant negative correlations were found between LA and varying muscle groups. LA at 25 mph correlated negatively with flexors, left lateral flexors, and left lateral rotators. LA at 40 mph correlated negatively with flexors, right and left lateral flexors, as well as the left lateral rotators	• Female players displayed significantly lower flexor and extensor strength, smaller head mass, head–neck segment length and girth than male athletes • Greater head impact kinematics in participants with weaker muscle groups, which may create a smaller effective head mass • Significant relationship between anthropometrics and soccer heading kinematics for sex and ball speeds
Caccese et al. [61]	Cross-sectional 100 soccer players (58 F, 42 M) Age: 17.1 ± 3.5 years	Head and neck size Neck strength Sex	Peak linear acceleration (g) Peak rotational acceleration (rad/s^2^)	The regression model assessing size-related predictors explained 22.1% of the variance (*R*^2^ = 0.221, *F*_(2,91)_ = 13.480, *p* < 0.001, *ƒ*^2^ = 0.28) in PLA and 23.3% of the variance (*R*^2^ = 0.233, F_(2,91)_ = 14.442, *p* < 0.001, *ƒ*^2^ = 0.30) in PRA. Head mass significantly predicted peak RA (*β* = − 0.404, *p* = 0.034) The regression model assessing strength-related predictors explained 13.3% of the variance (*R*^2^ = 0.133, *F*_(2,91)_ = 7.276, *p* = 0.001, *ƒ*^2^ = 0.15) in PLA and 17.2% of the variance (*R*^2^ = 0.172, *F*_(2,91)_ = 9.888, *p* < 0.001, *ƒ*^2^ = 0.21) in PRA. The sternocleidomastoid strength significantly predicted PLA and PRA (linear: * β* = − 1.544, *p* = 0.012; rotational: * β* = − 0.117, *p* = 0.018)	• Findings suggest that greater head and neck size and increased neck strength predicted lower PLA and PRA • The findings suggest that anthropometric and neck strength measures should be considered when determining readiness to begin soccer heading
Caccese et al. [62]	Cross-sectional 100 active soccer players (58 F,42 M) Youth female (*n* = 18)//male (*n* = 8) Age: 12.8 ± 0.9//13.0 ± 0.9 years High school female (*n* = 19)//male (*n* = 14) Age: 16.7 ± 0.9//6.9 ± 1.0 years Collegiate female (*n* = 21)//male (*n* = 20) Age: 19.3 ± 1.1//20.8 ± 1.3 years	Sex Age	Peak linear acceleration (g) Peak rotational acceleration (rad/s^2^)	The MANOVA revealed a significant multivariate main effect for sex (Pillai’sTrace = 0.165, F_(2,91)_ = 11.868, *p* < .001, ${\eta }_{p}^{2}$= 0.207, power = 0.994). Given the significance of the overall test, the univariate main effects were examined for sex. Significant univariate main effects for sex were obtained for resultant PLA (F(_1,92)_ = 23.427, *p* < 0.001, ${\eta }_{p}^{2}$ = 0.203, power = 0.998) and resultant PRA (F_(1,92)_ = 21.560, *p* < .001, ${\eta }_{p}^{2}$= 0.190, power = 0.996), whereby females had higher head resultant peak linear (40.9 ± 13.3 g) and rotational (3279 ± 1065 rad/s^2^) accelerations than males (27.6 ± 8.5 g, 2219 ± 823 rad/s2) The MANOVA did not reveal a significant multivariate main effect for age (Pillai’sTrace = 0.033, *F*_(4,182)_ = 0.646, *p* = 0.630, ${\eta }_{p}^{2}$= 0.014, power = 0.209). There was no difference in PLA or PRA across youth (38.5 ± 13.6 g, 3026 ± 1121 rad/s^2^), high school (33.5 ± 12.1 g, 2745 ± 942 rad/s^2^) and collegiate (34.8 ± 13.9 g, 2792 ± 1216 rad/s^2^) soccer players	• There was a significant main effect for sex in head acceleration. There was no significant main effect for age • Female soccer players have higher resultant peak linear and rotational head accelerations than male soccer players in these controlled, laboratory conditions
Chrisman et al. [44]	Cohort 46 youth soccer players (25 F, 21 M) Age: 11–14 years	Age Sex	Linear acceleration (g)	57% of the athletes sustained a head impact > 15 g during the 1-month study period, and males were more likely than females to be in this category (76% of males versus 40% of females, *p* = 0.02). When restricting the sample to the 25 athletes who sustained head impacts > 15 g, females sustained head impacts of greater magnitude than males (median 47.4 versus 33.3 g, *p* = 0.04). Older athletes experienced greater HIE than younger athletes. Only 15% of athletes on U12 teams sustained head impacts > 15 g, compared with 85% of athletes on U14 teams (*P* < 0.001). Multivariate analyses using Poisson regression to predict the quantity of HIE adjusting for clustering by team and stratifying by sex suggested that age effects were only significant for females (males: *p* = 0.53, females: *p* = 0.02)	• Males were more likely than females to experience at least one head impact above the threshold • Older athletes experienced more head impacts than younger athletes • However, of those who sustained head impacts > 15 g, females sustained head impacts of significantly greater magnitude than males
den Hollander and Gouttebarge [71]	Cohort/Observational descriptive 31 elite/professional players (16 F, 15 M) Age: F: 14–18 years; M: 22–33 years	Age Sex	Head acceleration (g)	Average head acceleration (g) of headers measuring over 10 g Females: 17.67 g Males: 24.2 g Females had no headers measuring greater than 80 g (max was 71 g), while males recorded five headers over 80 g (max was 137 g). Only 10% of headers by females were over 10 g; 74% of headers by males were greater than 10 g No standard deviations provided, unable to calculate mean difference or 95% CI	• Appears males had greater head acceleration than females – no statistical analysis to confirm the significance of this • Greater average acceleration and much greater percentage of impacts measuring over 10 g in male players
Dezman et al. [64]	Cross-sectional 16 subjects (8 F, 8 M) Age: 20.5 ± 1.9 years	Neck strength	Linear (translational) acceleration (m/s^2^) Angular acceleration (rad/s^2^)	Mean neck strength difference was positively correlated with AA (rho = 0.497; *p* = 0.05), with a trend toward significance for LA (rho = 0.485; *p* = 0.057). Correlation between mean flexion strength and head acceleration and mean extension strength and head acceleration were nonsignificant. Change in ball velocity did not correlate with either mean flexion strength (*P* > 0.05), mean extension strength (*P* > 0.05) or mean neck strength difference (*P* > 0.05)	• Symmetrical strength in neck flexors and extensors may reduce head acceleration of experienced collegiate players at low ball velocities • Suggests a correlation between neck strength imbalance and AA during heading
Filben et al. [88]	Cohort 14 female soccer players Age: 14.4 ± 0.9 years	Heading technique	Peak linear acceleration (g) Peak rotational acceleration (rad/s^2^) Peak rotational velocity (rad/s)	Standing headers: technique score (*p* = 0.043) and the technique score–session type interaction (*p* = 0.004) were associated with PRA, whereby better technique score had a negative relationship with PRA for headers during games but a positive relationship with PRA during practices. Technique score was not associated with PLA, PRV or HIP ratio (all *p* > 0.050), but *β* values suggest the relationship tended to be negative (4.9%, 3.9% and 8.9% decrease per 10-unit increase in technique score, respectively). Standing headers with ‘back extension’ criterion rated ‘yes’ were associated with a 7.8% reduction in PLA compared with headers rated ‘no’ or ‘mixed.’ Standing headers with ‘body is side on’ criterion rated ‘yes’ were associated with a 19.6% reduction in PRV compared with standing headers rated ‘no.’ Jumping headers: neither the technique score–session type interaction nor the main effect of technique score were associated with any dependent variable (all p > 0.050), but *β* values suggest technique score tended to have a negative relationship with PLA, PRA and PRV (7.7%, 13.1% and 5.8% decrease per 10-unit increase in technique score, respectively). Jumping headers with back extension criterion rated ‘no’ were associated with a 78.4% increase in PRA and 39.7% increase in PRV compared with headers rated ‘yes’ or ‘mixed’	• Significant association between technique score and the technique score–session type interaction with PRA of standing headers, whereby better technique was associated with lower PRA in games but not in practices • Trends reported herein suggest that heading technique – particularly during games and higher-magnitude impacts – may be associated with small reductions in head impact magnitude in female youth soccer players, but additional study with a larger sample size is required to confirm these relationships
Gutierrez et al. [65]	Cross-sectional 17 female varsity high school players Age: 15.9 ± 0.9 years	Neck strength	Peak linear acceleration (g)	Forward header peak acceleration (g) Neck flexion strength: *r* = − 0.639, *p* = 0.008 Neck extension strength: *r* = − 0.632, *p* = 0.009 L lateral flexion strength: *r* = − 0.608, *p* = 0.012 R lateral flexion strength: *r* = − 0.610, *p* = 0.012 Left header peak acceleration (g) Neck flexion strength: *r* = − 0.541, *p* = 0.030 Neck extension strength: *r* = − 0.545, *p* = 0.029 L lateral flexion strength: *r* = − 0.621, *p* = 0.010 R lateral flexion strength: *r* = − 0.500, *p* = 0.048 Right header peak acceleration (g) Neck flexion strength: *r* = − 0.701, *p* = 0.003 Neck extension strength: *r* = − 0.685, *p* = 0.003 L lateral flexion strength: *r* = − 0.757, *p* = 0.001 R lateral flexion strength: *r* = − 0.688, *p* = 0.003 Moderate, consistent negative correlations (*r* = − 0.500, 0.757; *p* < 0.05) were found for all directions of neck strength tested and resultant head acceleration in the header drills	• Increased neck strength was related to decreases in the magnitude of impacts during heading • Statistically significant (*p* < .05), moderate, negative correlations (*r* = –0.500, 0.757) between isometric neck strength and resultant head impact for all three-header directions • Suggests that neck strength is an important variable in minimizing impact during soccer heading
Kalichova and Lukasek [91]	Controlled laboratory study – one-off measurement 63 footballers of four age categories U11–U17 16 U11 players with average age: 10.3 years 15 U13 footballers with average age: 12.4 years 18 U15 players with average age: 14.4 years 14 U17 players with average age: 16.3 years	Age	Linear acceleration (g)	For this analysis, comparison was made between the youngest (U11) and oldest (U17) age groups U11 0.5 m: − 6 ± 1.6 g 1 m: − 7.8 ± 1.5 g 1.5 m: − 10.2 ± 2.6 g U17 0.5 m: − 3.5 ± 1.4 g 1 m: − 5.4 ± 2.2 g 1.5 m: − 8 ± 2.5 g *Calculated mean difference (95% CI) 0.5 m: 2.5 (1.37, 3.63) 1 m: 2.4 (1.01, 3.79) 1.5 m: 2.2 (0.29, 4.11)	• Significant differences in maximum head accelerations between U11 and U17 players. The U17 players had lower head acceleration values than the U11 players across all three ball speeds • A relationship with age showed a tendency of decreasing maximum head acceleration with increasing age • In U11 and U13 a smaller, lighter ball was used. The aim was not to create homogeneous conditions for all groups but rather to establish differences between headings in various age groups with their usual playing balls
Larson et al. [92]	Controlled laboratory study – one-off measurement 13 Division II soccer athletes (7 F, 6 M) Age: 18–22 years	Neck strength Sex	Linear acceleration (g)	Positive correlations between neck strength and accelerations experienced by the head during bouts of flick-on type headers; significance was not met between linear or rotational strength and high clear or driven headers The neck strength of males was significantly greater across both linear and rotational measures (*p* < 0.01), and males had higher acceleration values across all three header types. Specifically, accelerations (g) experienced by the head during driven type headers were significantly (*F*_(1,12)_ = 5.15, *p* = 0.04) higher for males (14.07 ± 1.45) than females (12.43 ± 1.16) When pooling the acceleration data across the three header types for each sex results are as below: Males: 14.73 ± 1.60 Females: 13.24 ± 1.90 *Calculated mean difference (95% CI) 1.49 (− 0.68, 3.66) For females, a negative correlation was supported between linear neck strength and driven type headers, r(7) = − 0.73, *p* = 0.03. As linear neck strength increased for females, the accelerations experienced by the head during driven-type header trials decreased	• Regarding heading and neck strength during flick-on headers, the greatest accelerations were experienced among athletes with the greatest neck strength • Males experienced significantly greater accelerations for flick-on headers; however, there were no significant differences between males and females for either driven or high-clear headers or when pooling data to combine all three header types • The role of neck strength in soccer heading is not simple; stronger muscles do not necessarily attenuate accelerations experienced by the head during heading
Muller and Zentgraf [59]	Non-randomised study of interventions 37 players Age: 15–18 years 22 players (7 F, 15 M) Age: 15–18 years 28 participants involved in the intervention section of the study 14 intervention (8 F, 6 M) 14 usual training group (5 F, 9 M) Age: 15–18 years	Neck strength Sex	Peak linear acceleration (g)	Cylindrical neck volume (CNV) was a significant predictor of total acceleration during low velocity purposeful headers (*p* = 0.005) The low ball velocity condition revealed a significant regression equation with *R*^2^_adj_ = 0.24 (*F*_(1,20)_ = 7.60, *p* = 0.012) in which functional neck strength assessment (FNSA) was the only significant predictor of total acceleration. We estimated that during low ball velocity conditions, the players’ total PLA decreased by 0.53 g for every 10 newtons (N) increase in neck strength in flexion. For male players during high ball velocity impacts, FNSA was once more the only significant predictor of total PLA with *R*^2^_adj_ = 0.54 (*F*_(1,13)_ = 17.60, *p* = 0.001). Results indicated that total PLA decreased by 0.84 g for every 10 N increase in neck strength in flexion during the high ball velocity condition. Comparing PLA in female and male players revealed a significant effect of sex on acceleration variables (F_(3,18)_ = 6.29, *p* = 0.004, ${\eta }_{p}^{2}$ = 0.51). However, we found no significant differences in acceleration variables after adjusting for CNV (*F*_(3,17)_ = 0.51, *p* = 0.249, ${\eta }_{p}^{2}$ = 0.21), with corresponding small (head acceleration) and moderate effects (total and trunk acceleration)	• Higher PLA for females compared with males while heading a ball traveling at 9.6 m/s. However, this effect was not present after adjusting for CNV • Maximum isometric strength was associated negatively with kinematic responses during purposeful headers. We found that functional neck strength in flexion best predicts total PLA • Important to integrate functional exercises and strength assessments replicating the muscular demands of a specific task (e.g. purposeful header) or sport
Nevins et al. [46]	Cohort 23 high school soccer players (15 F, 8 M) Females Age: 15.33 ± 1.01 years Males Age: 16.75 ± 1.09 years	Sex	Peak linear acceleration (g) Peak angular acceleration (rad/s^2^)	Males PLA (g): 33.2 (11.6–69.6) PAA (rad/s^2^): 6923 (1089–15,864) Females PLA (g): 23.2 (10.1–70.6) PAA (rad/s^2^): 5515 (635–18,042) Maximum impact magnitudes observed over the seasons were comparable between the groups, and ball-to-head impacts resulted in the largest median and maximum impact magnitudes (PLA and PAA) for males and females. There was a statistically significant difference in the distribution of impact magnitudes between males and females for both PLA (*D* = 0.26, *P* < 0.01) and PAA (*D* = 0.28, *P* < 0.01), indicating that the distribution of impacts for male participants included a larger proportion of high magnitude impacts	• Males experienced direct head impacts 48% more often than females, owing to more frequent heading of the ball • Males were also more likely to experience higher impact acceleration magnitudes than females (on average 34% higher PLA and 76% higher PAA), but maximum impact magnitudes were comparable for males and females overall (1% difference for PLA and 16% difference for PAA)
Pereira et al. [79]	Randomised repeated measures 17 recreational adult football players (3 F, 14 M) Age: 22 ± 3.5 years	Peak isometric neck flexor and extensor strength Sex	Peak linear acceleration (g) Peak angular velocity (rad/s)	Strength related predictors for LA (g) and AV (rad/s) Peak isometric neck flexor strength LA: *R*^2^ = 0.17, *p* = 0.031, *F* = 15.6 AV: *R*^2^ = 0.11, *p* < 0.001, *F* = 8.4 Peak isometric neck extension strength LA: *R*^2^ = 0.19, *p* < 0.001, *F* = 18.5 AV: *R*^2^ = 0.07, *p* < 0.021, *F* = 6.48 For PLA, 17% of the variance was explained by peak isometric neck flexor strength, and 19% of the variance was explained by peak isometric neck extensor strength. For PAV of the head, 11% of the variance was explained by peak isometric neck flexor strength and 7% by peak isometric neck extensor strength	• Head kinematics are influenced by maximal isometric neck strength • Peak isometric neck flexor and peak isometric neck extensor strength significantly predicted PAV and PLA
Robinson [101]	Mixed laboratory-based and live game play data collection 14 varsity women’s soccer players	Age Heading proficiency	Peak linear acceleration (g) Peak rotational acceleration (degree/s^2^) Peak rotational velocity (degree/s)	Players 4 and 9 were the youngest of the participants and had two of the highest PLA values (44.6 and 34.6 g). There appears to be a relative consistency of minimum average values for PLA, PRA, PRV and duration for players considered to have ‘excellent’ heading proficiency Players 14 was evaluated as having ‘poor’ heading form. Player 14 recorded maximum average PLA, PRA, PRV and duration for the 5-yard stationary passing header, and player 14’s values for this header were all at least 42% higher than the next highest magnitude header type values	• No statistical analysis undertaken so cannot comment on significance of results. From the raw data it appears that the youngest players and the player assessed as having ‘poor’ heading technique had higher head acceleration measures
Saunders et al. [48]	Cohort 28 NCAA Division III soccer players (16 F, 12 M) Females Age: 19.94 ± 1.06 years Males Age: 20.25 ± 1.14 years	Sex	Linear acceleration (g) Rotational acceleration (degree/s^2^)	LA (g) Males: 19.48 ± 14.57 Females: 22.31 ± 12.19 RA (deg/s^2^) Males: 185,531.39 ± 192,426.01 Females: 262,047.11 ± 186,254.19 Pairwise differences in head impact magnitude across sex: mean difference (men to women) (95% CI), *p*-value PLA: − 2.824 (− 4.30, − 1.35), *p* < 0.001 PRA: − 76,515.716 (− 97,612.50, − 55,418.93), *p* < 0.001	• Women had the highest head impact frequency when heading a soccer ball, while men were most likely to sustain head to body contact • Women sustained ball-to-head impacts with significantly higher LA and RA than male players
Tierney et al. [83]	Controlled laboratory study – one-off measurement 44 soccer players (29 F, 15 M) Females Age: 19.5 ± 1.8 years Males Age: 20.3 ± 2.9 years	Sex Head–neck segment anthropometrics Isometric neck strength	Linear acceleration (g)	Women exhibited 15% less head–neck segment mass, 5% less head–neck segment length and 12% less neck girth compared with men. Women also exhibited 50% less isometric neck flexor strength and 53% less isometric extensor strength compared with men Head acceleration was 10% greater in women than in men during free heading (20.16 ± 4.12 g versus 18.25 ± 4.48 g). However, this was not statistically significant (*t*_1,42_ = 1.42, *p* = 0.164)	• Women had lower anthropometric values and significantly less neck flexor and extensor strength than males • Although there was some increase in head acceleration in females when compared with males, this was not statistically significant
Worsey et al. [67]	Cross-sectional Eight participants for the field heading tests ranged from novice to semi-professional soccer players Age: 27 ± 7.2 years Neck Muscle Activity (EMG) Test: One healthy male with no prior experience of playing competitive soccer	Playing experience Activation of neck muscles	Head acceleration (g)	Novice participants have a much larger ratio and larger range of values when compared with more experienced and semi-professional players To explore this further, an experienced player gave one novice player instruction to activate the neck muscles at the time of impact. A paired sample *t*-test found a significant difference in EMG voltage before and after muscle activation. A paired sample *t*-test also found that there was a significant difference between the impact ratio before and after muscle activation (*p* = 0.008). Following muscle activation there was a clear increase in the muscle activity and decrease in the acceleration ratio. There was also a mean percentage decrease of 34% in the impact ratio after muscle activation (2.00 ± 0.58 and 1.49 ± 0.32) before and after, respectively	• Suggests that players with higher experience have a different heading technique, which reduces the acceleration impact • The results from this investigation suggest that increasing neck muscle activity reduces the impact acceleration of the head and, in turn, the force experienced solely by the head

*AA* angular acceleration, *AV* angular velocity, *CI* confidence interval, *CNV* cylindrical neck volume, *degree/s or dps* degree per second, *EMG* electromyography, *FNSA* functional neck strength assessment, *g* gravitational acceleration, *HA* head acceleration, *HIE* head impact exposure, *HIP* head impact power, *krad/s* kiloradian per second, *LA* linear acceleration, *MANOVA* multivariate analysis of variance, *m/s* metres per second, *mph* miles per hour, *PAA* peak angular acceleration, *PAV* peak angular velocity, *PLA* peak linear acceleration, *PRA* peak rotational acceleration, *PRV* peak rotational velocity, *RA* rotational acceleration, *rad/s* radian per second, *RV* rotational velocity

^*^Indicates where mean difference and 95% CI have been calculated by the authors of this review on the basis of raw data provided in the included studies

##### Neck Strength

Female athletes exhibited significantly lower maximal neck strength compared with males [63, 83, 92]. Several studies found a negative correlation between neck strength and head acceleration, meaning players with weaker necks had greater head kinematics [59, 61, 63, 65, 79, 83]. The significance of this result varied between studies; some papers found strength only correlated with either PLA or PRA, and others found that only the strength of certain muscle groups correlated with head acceleration. One study showed moderate, consistent negative correlations with all directions of neck strength and resulting LA, suggesting that increased neck strength was related to reduced magnitude of head impacts during heading [65]. Examining the strength ratio between neck flexors and extensors, one study found that having equal strength in the neck flexors and neck extensors may reduce head acceleration [64]. Pereira et al., [79] found that peak isometric neck flexor and extensor strength significantly predicted PLA and PAV, with higher maximal isometric neck strength reducing head acceleration. Most of these studies used isometric neck strength as the outcome measure, either using a hand-held dynamometer or some form of fixed dynamometer. From the study methods, it appears these studies used ‘make’ measurements (exerting a maximal force against a fixed resistance). However, Muller and Zentgraf [59] included a novel functional neck strength assessment. They found that functional neck strength assessment was the only significant predictor of head acceleration [59]. Every 10 N increase in neck flexor strength, specifically, predicated a reduction for PLA by 0.53 g and 0.84 g for low- and high-velocity conditions, respectively [59].

A study investigating different header types found conflicting results in relation to the association with neck strength [92]. Neck strength positively correlated with head acceleration during flick-on headers, meaning athletes with greater neck strength had greater head acceleration [92]. For driven headers, there was a statistically significant negative correlation between linear neck strength and head acceleration in females, suggesting that increased neck strength reduced head acceleration [92]. Another study found that when an experienced player gave a novice player the instruction to activate the neck muscles, there was an increase in neck muscle activation and a reduction in head acceleration [67].

##### Anthropometric Variables

Smaller neck girth [63] and smaller head size [61] were both shown to have a relationship with greater LA and RA. Cylindrical neck volume was also a significant predictor of head acceleration during low velocity headers [59]. Female players had reduced head–neck segment mass (body mass × sex-specific head–neck segment to total body mass percentage) and length and less neck girth than male players, and their head acceleration was 10% higher than men (not statistically significant) [83]. There was no significant relationship between head–neck–torso alignment and head acceleration [68].

##### Demographic Characteristics

In terms of biological sex, there was some consistency that female players sustained greater head accelerations than male players, with increased PLA and PRA [48, 62] and increased RV [63] during purposeful headers. A further study also found higher PLA in females; however, when adjusted for cylindrical neck volume, there was no difference between sexes [59]. Male players sustained more head impacts owing to performing headers more frequently than female players [44, 46]; however, when considering specifically at the head impacts measuring greater than 15 g, females sustained head impacts of greater magnitude [44]. In contrast to this, one study found males had a larger proportion of higher impact acceleration magnitude than females; however, the maximum impact magnitudes were comparable between sexes [46]. Furthermore, den Hollander et al. [71] found that males had greater head acceleration than female players, but this relationship’s significance was not investigated.

Regarding age, in one study, as age increased, head acceleration decreased, with significant differences between under 11 years and under 17 years age groups [91]. One study concluded that older athletes experienced greater head-impact exposure than younger athletes (owing to more frequent heading), and that the age effect in this study was only significant for female players [44]. A descriptive study found that the two youngest participants had two of the highest PLA values of the participants included [101]. In contrast to these findings, one study found no difference in PLA or PRA across youth (12–14 years), high school (15–17 years), and collegiate (18–24 years) aged players [62].

Regarding heading proficiency/technique, two studies examined heading proficiency (identified as excellent, good, decent or poor by coaching staff) and experience. Novice players [67] and those considered to have ‘poor’ heading technique [101] had increased head acceleration and a larger range of head acceleration. Those with ‘excellent’ heading technique [101] and those with more playing experience [67] had more consistent head acceleration. One study focused more specifically on heading technique and found that better technique reduced PRA in practices but not in games, and reported trends suggest that technique may be associated with small reductions in head impact magnitude; however, this study was underpowered [88].

#### Neck Training Interventions

In total, 8 studies assessed the effect of neck or plyometric training interventions to improve neck strength or neuromuscular control, with the aim of altering head kinematics during a header (Table 10) [17, 51, 52, 54–56, 59, 60]. Protocols had different durations and exercises and identified conflicting results regarding the effect of neck training programmes on head kinematics. A potential confounder in several studies was that the control group(s) continued with ‘usual training’ which could affect strength and control, depending on what ‘usual training’ entails.

**Table 10:** Results for the effect of neck training interventions

Study	Design/participants	Independent variable(s)	Head acceleration measures	Results	Conclusion
Becker et al. [56]	Non-randomised study of interventions 33 male soccer players Age: 20.3 ± 3.6 years Two intervention groups IG1 – adult team (*n* = 11) IG2 – youth team (*n* = 9) Control group: CG (*n* = 13)	Intervention: A 6-week neck strength training for the neck flexors and extensors. Participants in IG1 and IG2 completed two training sessions per week with three exercises (neck flexion, neck extension and isometric head pushing). Neck flexion and extension exercises were performed with the addition of an elastic rubber band (Thera-BandTM, Artzt GmbH, Dornburg, Germany), in which the rubber bands were changed every four training sessions to increase the intensity	Head acceleration (g)	The IMVC for neck flexion increased in all groups. The IMVC for neck extension decreased for IG1 and CG and increased for IG2 In relation to head acceleration, there was no significant changes for standing header (time: *p* = 0.906; time*group: *p* = 0.460), jumping header (time: *p* = 0.218; time*group: *p* = 0.295), running header (time: *p* = 0.119; time*group: *p* = 0.362), post-jumping header (time: *p* = 0.117; time*group: *p* = 0.833) or post-running header (time: *p* = 0.219; time*group: *p* = 0.507) The training-induced alterations (within group and between group) did not show any significant changes for pre–post difference (Δ) standing header (*p* = 0.460), Δ jumping header (*p* = 0.295), Δ running header (*p* = 0.362), Δ post-jumping header (*p* = 0.833) or Δ post-running (*p* = 0.507). No correlations could be established between the change of force for neck flexors (*p* = 0.683) and neck extensors (*p* = 0.104) nor for the changes in head acceleration between the groups (*p* = 0.780)	• This training programme did not lead to significant increases in strength in the intervention groups • No significant changes in head acceleration for any of the header types • Changes in force do not allow prediction as to the changes in head acceleration
Doewes et al. [55]	Non-randomised study of interventions – one group pre-test post-test design Three female football players Age: 18–22 years	Intervention: An 8-week neck flexor and extensor strengthening program. Participants underwent the intervention for 8 weeks with three sessions per week. Strength training for neck flexors and extensors was performed with neck machine flexion and extension, partner assisted neck resistance and head-ball-head isometrics. Exercise duration from 10 to 25 s, 6–8 reps, 3–6 sets and 15–30 s rest between sets	Angular velocity (rad/s) Angular acceleration (rad/s^2^)	Standing header AV: pre-test: 0.04, post-test: 0.04, MD: 0.01, sig: 0.184 AA: pre-test: 0.005, post-test: 0.009, MD: 0.004, sig: 0.074 Jumping header AV: pre-test: 0.08, post-test: 0.12, MD: 0.04, sig: 0.554 AA: pre-test: 0.031, post-test: 0.076, MD: 0.044, sig: 0.105 Running header AV: pre-test: 0.09, post-test: 0.23, MD: 0.14, sig: 0.098 AA: pre-test: 0.058, post-test: 0.233, MD: 0.175, sig: 0.125 The intervention did not show statistically significant differences in heading biomechanics in standing, jumping or running headers	• No significant effect of strength training neck flexors and extensors on the biomechanics of heading
Muller and Zentgraf [59]	Non-randomised study of interventions 37 players Age: 15–18 years 22 players (7 F, 15 M) Age: 15–18 years 28 participants involved in the intervention section of the study 14 intervention (8 F, 6 M) 14 usual training group (5 F, 9 M) Age: 15–18 years	Intervention: A 14-week neck-strengthening and neuromuscular training was implemented twice weekly for 15 min before the start of each regular training session as part of a warmup. An additional home workout plan was available when team training was not possible. Exercises included isometric and dynamic resistance exercises for the neck muscles, isometric exercises for the trunk, plyometric exercises for the trunk and shoulder girdle and perturbation exercises for the neck muscles, while isometrically engaging the trunk musculature. Exercise sessions consisted of a warm-up and an additional five to seven exercises. By selecting different exercises and varying movements, the training program followed a progressive design across group workouts Controls continued regular training	Peak linear acceleration (g)	Repeated-measures MANOVAs revealed significant differences between the intervention group and controls on strength variables and CROM, *F*_(8,18)_ = 4.19, *p* = 0.006, ${\eta }_{p}^{2}$ = 0.65. Univariate tests indicated significant intervention effects on all strength variables, whereas we found no effects on either frontal plane strength symmetry or overall CROM Significant differences in PLA variables were found between pre-intervention and post-intervention measurements for low but not for high ball velocity conditions: *F*_(2,12)_ = 4.77, *p* = 0.030, ${\eta }_{p}^{2}$ = 0.44 and *F*_(2,4)_ = 6.36, *p* = 0.057, ${\eta }_{p}^{2}$ = 0.76, respectively. The mean total PLA decreased by 22.4 g (95% CI 24.0–20.8, *p* = 0.008), the mean trunk PLA decreased by 20.8 g (95% CI 21.6–0, *p* = 0.044) and the mean head PLA decreased by 21.5 g (95% CI 22.6–20.4, *p* = 0.009) during low ball velocity conditions. Similar results were found in the high ball velocity condition in male players: the mean total PLA decreased by 22.4 g (95% CI 25.6–0.7), the mean trunk PLA decreased by 21.1 g (95% CI 21.8–20.3), and the mean head PLA decreased by 21.4 g (95% CI 24.5–1.7). Univariate tests demonstrated beneficial intervention effects with large effect sizes (${\eta }_{p}^{2}$< 0.21)	• The results indicate the efficacy of the exercise intervention for increasing neck and trunk muscle strength • Maximum isometric strength is associated negatively with kinematic responses during purposeful headers • Functional neck strength in flexion best predicts total PLA, which points to the importance of integrating functional exercises and strength assessments replicating the muscular demands of the specific task (e.g. purposeful header) or sport
Omdal [51]	RCT Eight high school female soccer players (four intervention, four control) Age: 16.24 ± 1.07 years	Intervention: An 8-week plyometric and functional training program was incorporated into regular training schedules for the intervention group. The program consisted of three drills to be performed three times per week: partner ball tosses for accuracy (3-m, low velocity tosses performed from a straight-ahead position, as well as 90° right and left of the participant with the return passes attempted as accurately as possible), partner ball tosses for distance (6-m, medium velocity headers executed with maximal effort to a straight-ahead area, as well as 3 m to the left and right of the designated target) and partner ball tosses for speed (athlete to be seated in a hook lying position while completing as many return passes as possible in a 20 s time period)	Peak linear acceleration (g)	Significant differences were found over time in *z*-axis amplitude area (*p* = 0.04) and normalized mean peak acceleration (*p* = 0.04). Significant differences occurred between groups related to normalized mean peak impact (*p* = 0.01). The control group decreased in mean peak impact from 1.157 to 1.136 g force, while the mean peak impact group increased among the intervention group from 1.197 g to 1.235 g. No significant results were identified after an analysis of maximum peak G force value over time, mean peak acceleration specific to each heading trial or maximum peak acceleration regardless of axis (*p* ≥ 0.05) Peak accelerations were higher in the intervention group compared with the control (*p* ≤ 0.01), with those who performed the heading intervention increasing their normalized mean peak impact by 0.038 g, while the mean peak acceleration of control subjects decreased by 0.021 g No significance was identified related to neck strength when comparing pre- and post-test isometric strength measurements	• No significance was identified related to neck strength when comparing pre- and post-test isometric strength measurements • Peak accelerations were higher in the intervention group compared with the control (*p* ≤ 0.01) • The increase in normalized peak acceleration in the intervention group may be due to increased neuromuscular control from the repetitive heading training, given that an emphasis of the sport-specific intervention was to improve the ability to control the ball for both accuracy and power
Peek et al. [17]	Pilot randomised control trial 52 players completed the study (31 intervention, 21 control) Intervention group (14 F, 17 M) Age: 14.35 ± 0.29 years Control group (7 F, 14 M) Age: 14.95 ± 0.26 years	Intervention: Players completed the FIFA 11 + injury reduction programme with the addition of three neck exercises, three times per week for 5 weeks. For the first exercise, based on the Versteegh Roll and Tuck (VRT), players were instructed to sit on the ground and hold their knees before rolling backwards and forwards as fast as they could whilst keeping their chin tucked in and not allowing their head to touch the ground/floor. Players completed the VRT for one set of the following (each lasting 30 s): VRT with the head (1) pointed straight ahead, (2) turned to the left and (3) turned to the right Controls completed the FIFA 11 + as usual without the addition of neck exercises	Peak linear acceleration (g) Peak angular velocity (dps)	Mixed-model ANOVAs for the combined and girls’ data revealed large effects for neck strength variables (*η*^2^ > 0.22; *p* < 0.001), medium effects for PLA (*η*^2^ > 0.08; *p* < 0.05) and medium effects for PAV (η^2^ > 0.07; *p* < 0.05) between the intervention and control groups over time. Boys’ data demonstrated large effects for neck strength variables (η^2^ > 0.28; *p* ≤ 0.001), medium effects for PLA (η^2^ 0.13; *p* = 0.05) and small effects for AV (η^2^ 0.03; *p* = 0.13), although neither PLA nor PAV were statistically significant Intervention players demonstrated greater increases in mean composite neck strength (53.8% intervention versus 15.6% control) as well as greater decreases in mean PLA during heading (− 11.8% versus − 5.0%) from baseline to follow-up when compared with control players. Reduction in PAV was more pronounced in girls (− 27.7%) than in boys (− 11.5%) in the intervention group	• Boys and girls in the intervention group demonstrated an increase in isometric neck strength and a decrease in head impact magnitude (most notably PLA for all players and PAV for predominantly girls) when compared with control players • Given these exercises took less than 2 min to complete and that they were perceived as feasible and acceptable, the integration of neuromuscular neck exercises into the FIFA 11 + for adolescent players warrants further investigation
Wahlquist et al. [54]	Non-randomised study of interventions 27 female youth soccer players (13 control, 14 intervention) Age: 10.8 ± 0.5 years	Intervention: While adhering to their normal practice and game routines, the experimental group also participated in the Get aHEAD Safely in Soccer™ program, which included drills that taught appropriate purposeful heading along with neck/core strengthening exercises. Once a week, the experimental group would complete a header drill(s) involving 15–20 purposeful headers using a lightweight ‘Header Trainer’ ball. In addition, twice weekly, a member of the research team directed the experimental group in completing the neck/core strengthening exercises outlined in the Get aHEAD Safely in Soccer™ program The control group conducted normal soccer practices and games, which did not include any neck strengthening or heading techniques	Peak linear acceleration (g) Peak rotational acceleration (krad/s^2^) Peak rotational velocity (rad/s)	There was a significant main effect for time (*p* = 0.001) for neck flexion whereby all players improved from pre- to post-season. There was a 20.75% increase in neck flexion strength in the experimental versus an 11.9% increase in the control group. Interestingly, there was also a significant main effect for time (*p* = .039) and a group × time interaction (*p* = 0.008) where the control group showed significant improvement or increase in neck extension strength as compared with the experimental group. In stark contrast to the neck flexion strength gains, the experimental group showed a slight decline (− 2.16%) in strength over the 3.5-month period. Both groups showed improvement in torso flexion strength pre- to post-testing (*p* = 0.001). Improvement included a 21% gain in the control group compared with a 15.98% gain in torso flexion strength in the experimental group. There were no statistically significant changes in torso extension strength for either group, even though there was a 12.83% improvement pre- to post-season in the experimental group versus a very small (0.48%) improvement in the control group. There were no significant differences in head impact kinematics observed between the control and experimental groups in PLA (*p* = 0.692), PRA (*p* = 0.379) or PRV (*p* = 0.852)	• Following the Get aHEAD Safely in Soccer™ program, some positive changes were seen in neck and torso strength; however, there were no changes in head impact kinematics
Wahlquist and Kaminski [60]	Non-randomised study of interventions 12 female soccer players Age: 10.5 ± 0.5 years	Intervention: During the soccer season, participants took part in the Get aHEAD Safely in Soccer™ program. Twice a week, players participated in neck and core strengthening exercises. Once a week, players took part in appropriate purposeful heading drills which included 15–20 purposeful headers using lightweight, size 5 “Header Trainer” soccer balls	Peak linear acceleration (g) Peak rotational velocity (rad/s) Peak rotational acceleration (krad/s^2^)	Head impact kinematics were recorded for pre- and post-season acute heading sessions for eight players Pre-season PLA (g) 22.6 ± 4.9 PRA (krad/s^2^) 2.7 ± 1.3 PRV (rad/s) 12.1 ± 3.6 Post-season PLA (g) 24.7 ± 11.4 PRA (krad/s^2^) 2.4 ± 1.3 PRV (rad/s) 10.7 ± 5.5 *Calculated mean difference (95% CI) PLA: − 2.10 (− 9.53, 5.33) PRA: 0.30 (− 0.80, 1.40) PRV: 1.40 (− 2.54, 5.34)	• No change in PLA, PRA or PRV following the intervention • Although there were no changes in head impact kinematics, coaches and researchers noted an improvement in heading technique
Waring et al. [52]	Randomised control trial 20 varsity collegiate soccer players (12 F, 8 M) Age: 20.15 ± 1.35 years	Intervention: The experimental group followed a neck-strengthening program for 6 weeks as part of the normal strength and conditioning program (both groups participated in the same soccer specific strength and conditioning program – controls did not perform neck exercises). The Shingo Imara™ (Shingo Imara, Ann Arbor, MI, USA) was used to provide resistance during the four neck-strengthening exercises (resisted cervical flexion, extension and lateral flexion on both sides from a seated position) while monitored by a certified strength and conditioning specialist	Peak linear acceleration (g) Peak rotational acceleration (degree/s^2^)	There was no interaction present between group and time for PLA at 11.18 m/s (*F*_1,11_ = 0.66, *p* = 0.43, ${\eta }_{p}^{2}$ = 0.06, 1-* β* = 0.12) or at 17.88 m/s (*F*_1,11_ = 0.98, *p* = 0.34, ${\eta }_{p}^{2}$ = 0.08, 1-* β* = 0.15) There was no interaction present between group and time for PRA at 11.18 m/s (*F*_1,11_ = 0.00., *p* = 0.96, ${\eta }_{p}^{2}$ < 0.01 1-* β* = 0.05) or at 17.88 m/s (F_1,11_ = 0.002, *p* = 0.97, ${\eta }_{p}^{2}$ < 0.001, 1-* β* = 0.05) There was no interaction present between group and time for head impact duration at 11.18 m/s (*F*_1,11_ = 0.41, *p* = 0.53, ${\eta }_{p}^{2}$ = 0.04, 1-* β* = 0.09) or at 17.88 m/s (*F*_1,11_ = 0.08, *p* = 0.79, ${\eta }_{p}^{2}$ = 0.01, 1-* β* = 0.06)	• The neck strengthening program only significantly improved strength for the muscles in the anterior and anterolateral (right and left) directions • There were no significant findings for the heading biomechanics, with no changes in PLA or PRA seen after the intervention

*AA* angular acceleration, *ANOVA* analysis of variance, *AV* angular velocity, *CG* control group, *CI* confidence interval, *CROM* cervical range of motion, *degree/s or dps* degree per second, *g* gravitational acceleration, *HA* head acceleration, *IG* intervention group, *IMVC* isometric maximal voluntary contraction, *krad/s* kiloradian per second, *LA* linear acceleration, *MANOVA* multivariate analysis of variance, *m/s* metres per second, *mph* miles per hour, *PAA* peak angular acceleration, *PLA* peak linear acceleration, *PRA* peak rotational acceleration, *PRV* peak rotational velocity, *RA* rotational acceleration, *rad/s* radian per second, *RV* rotational velocity, *VRT* Versteegh roll and tuck

^*^Indicates where mean difference and 95% CI have been calculated by the authors of this review on the basis of raw data provided in the included studies

A 6-week strength training programme targeting the neck flexors and extensors using resistance bands and an isometric head push found no significant changes in either strength or head acceleration in the intervention group [56]. A total of three studies found that following strength interventions, there was significant improvements in various strength measurements, but this did not translate to changes in head acceleration or head kinematics [52, 54, 60]. Continuing this trend, a 6-week strengthening intervention of resisted neck flexion, extension and lateral flexion found significant anterior and anterolateral strength improvements, but no significant changes in PLA or PRA [52]. Moreover, Doewes et al. [55] found no effect on heading biomechanics following 8 weeks of neck flexor and extensor strengthening. One study implemented an 8-week plyometric and functional training programme that involved three different header drills performed three times per week [51]. This study found no significant changes in neck strength, and while the control group showed a slight reduction in head acceleration, the intervention group showed a small increase in head acceleration [51].

In total, two studies that incorporated neuromuscular exercises into the intervention showed changes in head kinematics [17, 59]. A 14-week intervention of neck and trunk strengthening and neuromuscular stabilisation exercises showed significant differences in all strength variables and a significant difference in PLA post-intervention for the low-ball velocity (but not high-ball velocity) conditions [59]. A 5-week intervention in youth soccer players using dynamic neuromuscular exercises showed a large effect in increasing isometric neck strength and a reduction in head impact magnitude when compared with controls [17]. PLA was reduced in both male and female players following the intervention [17]. The effect on AA was significant in females (27.7% reduction); however, only a small non-significant effect on AA was seen in male players (11.5% reduction) [17].

#### Game Scenario and Player Position

In total, 17 papers investigated the effect of game scenario and player position (Table 11) [45, 47–49, 66, 69, 70, 72–76, 81, 82, 84, 90, 102]. Most of these studies were undertaken with female players of youth and collegiate age groups during live practices or games.

**Table 11:** Effect of game scenario and playing position

Study	Design/ participants	Independent variable	Head acceleration measures	Results	Conclusion
Brooks et al. [69]	Cohort 36 female youth soccer players Age: 13.4 ± 0.9 years	Game scenario (corner kick, deflection, punt, goal kick, pass in the air, free kick and throw-in)	Linear acceleration (g) Angular velocity (rad/s)	In order of highest-lowest median LA (g): corner kick (31.4), goal kick (24.8), free kick (22.9), pass in air (16.2), throw in (16.0), punt (13.4) and deflection (9.9) In order of highest-lowest median AV ( rad/s): corner kick (39.6), goal kick (22.4), pass in air (19.1), free kick (18.9), throw in (13.7), punt (12.7) and deflection (9.5) The median (interquartile range) LA for the purposeful headers was 16.1 g (12.3–22.1), with a maximum of 74.8 g. The median AV was 16.2 rad/s (10.5–21.2), with a maximum of 50.6 rad/s	• Purposeful headers that occurred from corner kicks had the largest median magnitudes for both LA and AV • Purposeful headers that resulted from throw-ins, punts and deflections fell below the total median for LA and AV
Caccese et al. [70]	Cohort 16 female NCAA Div I collegiate soccer players Age: 19.6 ± 1.0 years	Game scenario: (bounce, secondary header, punt, throw-in, goal kick, corner kick and kick (base variable))	Peak linear acceleration (g) Peak rotational acceleration (krad/s^2^)	Base variable: kick (LA: 30.0 ± 19.5 g; RA: 7.5 ± 4.1 krad/s^2^) Bounce (16.2 ± 7.0 g) (*p* < 0.001; * β* = − 12.9) and secondary header (19.5 ± 12.9 g) (*p* = 0.002; * β* = − 10.3) impacts resulted in lower LA than the base variable Goal kick (38.8 ± 19.4 g) (*p* = 0.001; * β* = 8.9) impacts resulted in higher LA than the base variable Bounce (4.3 ± 3.3 krad/s^2^) (*p* < 0.001; * β* = − 2.9) and secondary header (5.2 ± 3.4 krad/s^2^) (*p* = 0.004; * β* = − 2.3) impacts resulted in lower RA than the base variable Goal kick (9.3 ± 3.9 krad/s^2^) (*p* = 0.004; * β* = 1.9) and punts (10.1 ± 4.8 krad/s^2^) (*p* = 0.002; * β* = 2.5) impacts resulted in higher RA than the base variable	• Purposeful headers from goal kicks and punts result in significantly higher head accelerations than headers of other strategic scenarios • Ball impact prior to heading, either with the ground or another athlete’s head, results in significantly lower head accelerations
Filben et al. [72]	Cohort 16 female NCAA Div I soccer players Age: 19.8 ± 1.24 years	Play state (i.e. the on-field scenario in which the header occurred) Intent (i.e. the objective of the player performing the header) Outcome (i.e. whether the header achieved its intended goal)	Linear acceleration (g) Rotational acceleration (rad/s^2^) Rotational velocity (rad/s)	Play state was associated with variance in peak LA, RA and RV (all *p* < 0.001). Headers during corner kicks, goal kicks, free kicks and live balls each had significantly greater mean peak LA (*p* = 0.002, *p* < 0.001, *p* = 0.039, *p* = 0.005, respectively), RA (all *p* < 0.001) and RV (all *p* < 0.001) than headers during drills. Headers during goal kicks had a significantly greater mean RA compared with headers during live ball scenarios (*p* = 0.025). Headers during goal kicks and corner kicks had the greatest 95th percentile peak LA, RA and RV among play states while drill headers had the lowest Intent was not associated with variance in peak LA (*p* = 0.057), RA (*p* = 0.074) or RV (*p* = 0.076). However, clearances had the greatest 95th percentile peak LA and RA while shots had the highest 95th percentile peak RV. Header outcome was associated with variance in peak LA (*p* = 0.009), but not RA (*p* = 0.259) or RV (*p* = 0.349)	• Play state was associated with variance in PLA, PRA and PRV • Headers during corner kicks, free kicks, goal kicks and live balls each had greater mean kinematics than headers during drills • Headers during goal kicks had a greater mean RA than headers during live ball scenarios
Filben et al. [73]	Cohort 19 female soccer players Six youth Age 15.27 ± 0.11 years 13 collegiate Age: 20.19 ± 1.34 years	Level of play Session type	Linear acceleration (g) Rotational acceleration (krad/s^2^) Rotational velocity (rad/s)	Compared with youth players, headers experienced by collegiate players had greater mean and 95th percentile PLA, PRV and PRA (all *p* < 0.001) Headers received from kicks had the greatest mean PLA and PRA (24.2 g, 2.51 krad/s^2^) among ball delivery methods followed by headers received from ‘other’ methods (13.0 g, 1.43 krad/s^2^), headers received from throws (11.0 g, 0.850 krad/s^2^) and headers received from headers (10.7 g, 0.779 krad/s^2^) Additionally, session type and ball delivery method were both significantly associated with PLA (*p* = 0.007, *p* = 0.001, respectively), PRV (*p* = 0.017, *p* < 0.001, respectively) and PRA (*p* = 0.021, *p* < 0.001, respectively)	• Both level of play and the session type and ball delivery method affect head acceleration • Collegiate soccer players experienced higher kinematic magnitudes during headers • Headers received from kicks had the greatest mean PLA and PRA
Filben et al. [45]	Cohort Female NCAA Division I soccer players, 24 player season (from 14 unique players) Age: 19.8 ± 1.24 years	Player position Session type Ball delivery	Linear head acceleration (g) Rotational head acceleration (krad/s^2^) Rotational velocity (rad/s)	LA (g); RA (krad/s^2^); RV (rads/s) Player position Defender: LA: 18.6 (15.4–22.6); RA: 1.75 (1.45–2.11); RV: 8.00 (7.07–9.05) Forward: LA: 19.7 (15.0–25.9); RA: 1.85 (1.43–2.39); RV: 8.61 (7.27–10.2) Goalie: LA: 6.25 (4.14–9.43); RA: 0.582 (0.389–0.872); RV: 7.91 (6.07–10.3) Midfielder: LA: 17.3 (12.6–23.9); RA: 1.86 (1.29–2.70); RV: 8.33 (6.53–10.6) Session type Practice: LA: 16.3 (12.4–21.3); RA: 1.58 (1.19–2.10); RV: 8.02 (7.34–8.73) Game: LA: 19.9 (14.6–27.3); RA: 1.86 (1.31–2.66); RV: 9.25 (7.96–10.8) Ball delivery Long kick: LA: 21.4 (18.6–24.6); RA: 2.25 (2.02–2.51); RV: 9.25 (8.46–10.1) All other methods: LA: 12.3 (10.4–14.6); RA: 1.00 (0.847–1.18); RV: 5.66 (5.03–6.38) The mean LA and RA of head impacts experienced by defenders, forwards and midfielders were each higher than that of the goalkeeper (LA: *P* = 0.002, *P* = 0.002, *P* = 0.007, respectively; RA: *P* = 0.002, *P* = 0.002, *P* = 0.004, respectively). No differences were found in RV between positions nor between defenders, midfielders and forwards No significant differences were found among mean peak kinematics between practices and games; however, games had greater 95th percentile head kinematics Headers delivered via long kicks had higher mean LA (*P* < 0.001), RA (*P* < 0.001) and RV (*P* < 0.001) than headers delivered via all other ball delivery methods combined	• Differences in head impact exposure were observed between session types with games having greater 95th percentile head kinematics. However, there were no significant differences among mean peak kinematics between practices and games • Player position was associated with differences in head kinematic magnitudes in LA and RA only when comparing the goalie to the other three positions • Headers delivered via long kicks had higher mean LA, RA and RV than headers delivered via all other ball delivery methods combined
Harriss et al. [90]	Cohort 36 female elite youth soccer players Age: 13.4 ± 0.9 years	Game scenario (pass in air, throw in, deflection, punt, shot, goal kick and corner)	Peak linear acceleration (g) Peak rotational velocity (degree/s)	On average, purposeful headers that occurred from shots resulted in the largest LA (27.35 ± 13.11 g), while corner kicks resulted in the largest RV (1447.42 ± 589.80 degree/s) The mixed effects model evaluating LA revealed that game scenario had a significant effect on the LA that resulted from purposeful headers, compared with the null model (χ^2^ = 37.97, *p* = 0.0001). Headers that occurred from passes in the air resulted in larger LA as compared with deflections (*t*_(417.79)_ = − 3.88, *p* = 0.0001) and smaller LA as compared with shots (*t*_(426.93)_ = 3.70, *p* = 0.002). There was significant interaction between head impact location and game scenario on LA, since the interaction model fit the data significantly better than the main effects model (*χ*^2^ = 20.10, *p* = 0.02). Punts resulted in significantly larger LA when completed with the top of the head compared with the front of the head (*t*_(410.26)_ = 3.34, *p* = 0.001). The mixed effects model evaluating RV indicated that game scenario had a significant effect on the RV that resulted from purposeful headers (*χ*2 = 20.84, *p* = 0.002). Passes in the air resulted in significantly larger RV compared with deflections (*t*_(419.58)_ = 3.20, *p* = 0.001) and throw-ins (*t*_(425.98)_ = 2.18, *p* = 0.03)	• Purposeful headers occurring from punts completed with the top of the head had the largest LA • There was no interaction between game scenario and head impact location for RV • Passes in the air accounted for the greatest proportion (41%) of purposeful headers performed by youth players, and shots were the only game scenario that resulted in larger head impact acceleration magnitudes • Passing the ball on the ground, rather than in the air could help reduce the number of recorded headers in this study sample by as much as 41%
Kenny et al. [74]	Cohort 13 female university varsity soccer players Age: 19.9 ± 1.6 years	Session type Player position	Peak linear acceleration (g) Peak angular acceleration (rad/s^2^) Peak angular velocity (rad/s)	When comparing session types, games were significantly higher than practices in mean PLA, PAV and PAA (*p* < 0.001). When comparing ball delivery methods, mean PLA and PAA in long kicks were significantly higher than short kicks, underhand throw, overhand throws, player-to-player and ground-to-player impacts (*p* < 0.001). Long kicks were not significantly different from overhand throws in AV (*p* < 0.5) but were significantly higher for all other comparisons in AV (*p* < 0.001). When comparing player position, mean peak kinematics by defenders were significantly higher than midfielders and forwards (*p* < 0.001) but there were no significant differences between midfielders and forwards	• Games and scrimmages resulted in significantly higher mean peak impact kinematics compared with practices, while the upper range of game/scrimmage and practice head impacts were comparable • Among different ball delivery methods, long kicks resulted in the highest peak kinematics • Greater mean peak kinematics in defenders
Lamond et al. [75]	Cohort 23 NCAA Division I female players Age: 19.7 ± 1.2 years	Playing position Playing scenario	Linear acceleration (g)	Header performed for shots (32.94 g ± 12.91 g, *p* = 0.02) and clears (31.09 g ± 13.43 g, *p* = 0.008) resulted in higher mean LA than passes (26.11 g ± 15.48 g) We found no differences in PLA across player position PLA did not differ between games (29.29 g ± 18.06 g) and practices (25.85 g ± 15.19 g, *p* = 0.09)	• No difference was evident in overall PLA between games and practices • Defenders and midfielders had the highest numbers of impacts, but head acceleration magnitude did not differ across player position • Headers for shots and clears had higher mean LA than passes
Ludwig [66]	Cross-sectional 24 female collegiate soccer players Frequent headers group Age: 24.2 ± 3.4 years Infrequent headers group Age: 25.4 ± 4.2 years	Frequent headers versus infrequent headers	Average linear acceleration	Average LA: Frequent headers: 194.97 ± 105.99 Infrequent headers: 339.09 ± 269.52 A statistical analysis of the average LA was not possible owing to violations of linearity and homogeneity of variances. On qualitative analysis, the infrequent header group had a higher average LA and greater variance in values than the frequent header group	• The higher values shown in the infrequent header group could indicate that they are receiving a greater blow to the head than the frequent header group • It could be posited that the technique displayed by the frequent header group somehow lessens the blow
Miller et al. [76]	Cohort Seven U14 female soccer players Age: 13.4 ± 0.6 years	Ball delivery method	Peak linear acceleration (g) Peak angular acceleration (rad/s^2^) Peak angular velocity (rad/s)	Mean LA was significantly greater for headers received from a kick compared with those received from another header (*p* < 0.0001) or a throw (*p* = 0.0003) and for headers received from a throw compared with those from another header (*p* < 0.0001) Headers from a kicked ball had significantly greater mean RV and mean RA than both headers from a thrown ball (both *p* < 0.0001) and from another header (both *p* < 0.0001) Statistically significant differences in mean LA, RV and RA between delivery methods. Heading the ball after a teammate headed it resulted in significantly greater mean LA than heading it to oneself (*P* < 0.0001). A long kick resulted in significantly greater mean LA (*P* < 0.0001), RV (*P* < 0.0001) and RA (*P* < 0.0001) than a short kick. Last, an overhead throw resulted in significantly greater mean LA (*P* = 0.0091), RV (*P* = 0.0037) and RA (*P* = 0.0134) than an underhand throw	• Although accounting for only 20% of head impacts, headers following a kicked ball resulted in significantly larger kinematic values than headers following a header or a thrown ball
Pritchard et al. [47]	Cohort Eight female youth soccer players Age: 13.5 ± 0.11 years	Session type: technical training versus game play	Linear acceleration (g) Rotational velocity (rad/s) Rotational acceleration (rad/s^2^)	Technical training LA: 8.18 (7.62, 8.78) RV: 3.80 (3.55, 4.07) RA: 0.55 (0.49, 0.60) Game play LA: 13.35 (12.05, 14.79) RV: 6.72 (6.10, 7.41) RA: 1.38 (1.20, 1.59) Not able to calculate MD and 95% CI owing to transformations of the data that were required for statistical analysis	• The mean head impact magnitude during technical training drills was lower than during game play • The total time that players were participating in technical drills was 46% greater than that of game play, and the rate of high magnitude head impacts was 43% lower during technical training compared with game play. Therefore, players sustain an equivalent number of high magnitude impacts in technical training and game play activities within a year
Saunders et al. [48]	Cohort 28 NCAA Division III soccer players (16 F, 12 M) Females Age: 19.94 ± 1.06 years Males Age: 20.25 ± 1.14 years	Session type: game versus practice	Linear acceleration (g) Rotational acceleration (deg/s^2^)	Practice Mean LA: 19.04 ± 10.96 Mean RA: 203,134.91 ± 162,365.40 Game Mean LA: 24.92 ± 14.92 Mean RA: 289,453.96 ± 217,569.09 *Calculated mean difference (95% CI) LA: 5.88 (− 1.13, 12.89) RA: 86,319.05 (− 16,538.61, 189,176.71)	• No significant difference between LA or RA between practices and games
Segars et al. [49]	Cohort 16 NCAA Div I female soccer players Age: 19.8 ± 1.24 years	Playing scenario: technical training versus set pieces	Peak linear acceleration (g) Peak rotational acceleration (rad/s^2^) Peak rotational velocity (rad/s)	Technical training, median (95th percentile) LA: 14.3 (33.6) RV: 6.4 (11.3) RA: 1033 (2958) Set pieces, median (95th percentile) LA: 25.0 (45.2) RV: 9.2 (18.6) RA: 2309 (5849)	• Technical training had the lowest LA, RV and RA • Set pieces had highest mean LA, RV and RA
Sokol-Randell et al. [81]	Cohort Ten male university soccer players Age: 20 ± 1 years	Ball delivery – cross, goal kick, long ball, pass	Peak linear acceleration (g) Peak angular acceleration (krad/s^2^)	Goal kicks had a significantly greater PAA than long balls (*p* = 0.024) and pass attempts (*p* = 0.011) No significant different in PLA between different ball delivery methods	• Goal kicks had significantly greater resultant PAA than long balls or pass attempts • Modifications to goal kicks present an opportunity to decrease peak speeds at which players head the ball, thereby reducing the cumulative exposure to head acceleration
Stelzer-Hiller et al. [102]	Cohort 15 male university soccer players Age: 20 ± 1 years	Pitch location – inside versus outside the penalty areas	Peak linear acceleration (g) Peak angular acceleration (krad/s^2^) Peak change in angular velocity (rad/s)	23.0% of all HAE from headers were below PLA of 10 g, 56.4% below 15 g, 84.8% below 20 g and 97.6% below 30 g Headers outside the box (*n* = 133, 81.6%) occurred more often than headers inside the box (*n* = 30, 18.4%) Preliminary analysis indicated all headers produced similar magnitudes for PLA, PAA and ∆PAV, regardless of pitch location	• No effect of pitch location was observed between heading kinematics (PLA, PAA and ∆PAV) inside and outside of the box
Stucker [82]	Cohort 25 Div I female collegiate soccer players Age: 19.58 ± 1.15 years	Game versus practice	Linear acceleration (g) Rotational acceleration (rad/s^2^)	Linear (*F*_1,24_ = 50.14, *P* < 0.001) and rotational (*F*_1,24_ = 19.28, *P* < 0.001) accelerations sustained during practice (20.4 g; 3230.7 rad/s^2^) were significantly greater than those sustained during games (18.5 g; 2898.5 rad/s^2^). There was an association between event type and severity of LA and RA; more severe head accelerations occurred in practices compared with games (*X*^2^[2] = 53.30, *P* < 0.001). Specifically, athletes were 1.13 times more likely to suffer a severe head acceleration in practice as compared with games. There was an association between event type and severity of RA; more severe head accelerations occurred during practices compared with games (*X*^2^[2] = 45.53, *P* < 0.001). Specifically, athletes were 1.18 times more likely to suffer a severe head acceleration in practice as compared with games	• Head accelerations (LA and RA) were more severe in practices compared with games
Tomblin et al. [84]	Cohort 14 youth female soccer athletes Age: 12–15 years	Player position Session type	Peak linear acceleration (g) Peak rotational velocity (rad/s) Peak rotational acceleration (rad/s^2^)	Between session types, practices (6.7 g) were associated with higher median LA than games (5.6 g) (*p* = 0.029), but games (5.8 rad/s) were associated with higher median RV than practices (4.9 rad/s) (*p* = 0.004) Game events were associated with higher 95th percentile LA, RV and RA (24.9 g, 15.2 rad/s and 2775.0 rad/s^2^) compared with practices (16.3 g, 13.2 rad/s and 1665.2 rad/s^2^) Stratified by game position irrespective of team, defenders experienced the highest 95th percentile LA and RA (30.2 g and 3791.9 rad/s^2^), while goalkeepers had the highest 95th percentile RV (16.5 rad/s). Player position did not have a significant effect on mean head kinematics	• Player position did not have a significant effect on mean head kinematics • The difference in means by session type resulted in conflicting results, with the mean LA significantly higher in practices and mean RV significantly higher in games; however, the difference in means was nominal (0.7 g and 0.8 rad/s, respectively) • Game events were associated with higher 95th percentile LA, RV and RA

^*^Indicates where mean difference and 95% CI have been calculated by the authors of this review on the basis of raw data provided in the included studies

*AA* angular acceleration, *AV* angular velocity, *CI* confidence interval, *degree/s* degree per second, *dps* degrees per second, *g* gravitational acceleration, *HA* head acceleration, *HAE* head acceleration events, *HIP* head impact power, *krad/s* kiloradian per second, *LA* linear acceleration, *m/s* metres per second, *PAA* peak angular acceleration, *PLA* peak linear acceleration, *PRA* peak rotational acceleration, *PRV* peak rotational velocity, *RA* rotational acceleration, *rad/s* radian per second, *RV* rotational velocity

Studies assessing different types of ball delivery found that headers performed from long range kicks, such as goal kicks, punts and corners, had the highest LA and RA [45, 69, 70, 72–74, 76, 90]. Sokol-Randell et al. [81] found that headers from goal kicks significantly increased PAA, but there was no significant difference in PLA. Headers from throw-ins, a header or a deflection tended to have lower head acceleration values [69, 70, 72, 74, 76]. Segars et al. [49] found that set pieces had greater LA, RA and RV compared with technical training (qualitative conclusion, as there was no statistical investigation).

Results for head acceleration in practices versus games were varied. A total of two studies found no difference in mean peak kinematics between practices and games [45, 75]; however, it was noted that the games had greater 95th percentile head kinematics compared with those collected at practice sessions across the season(s) [45]. One study found that games had significantly higher mean PLA and PRA than practices [74], and Pritchard et al. [47] found greater LA, RA and RV in games when compared with training. However, it was also noted that there was an equivalent number of high-magnitude impacts in training owing to greater time spent training compared with game play [47]. A further study showed greater acceleration in both PLA and PRA in games compared with practices, but this was found to be non-significant when calculating MD and 95% CI [48]. Conflicting with these findings, Stucker et al. [82] found that PLA and PRA sustained in practices was greater than in games, and players were 1.13–1.18 times more likely to sustain a severe head acceleration event in a practice compared with games [82]. Finally, the results of Tomblin et al. [84] were mixed, with LA being higher in practices and RA being higher in games. Regarding pitch location, no difference in head kinematics in headers performed inside or outside of the penalty box was found [102].

With respect to outfield player position, three studies measuring head impacts in games and practices across a playing season found no differences in mean head acceleration across defenders, midfielders and forwards [45, 75, 84]. One of these studies did note that defenders had the highest 95th percentile LA and RA [84]. Results from Kenny et al. [74], who collected data across two playing seasons, found significantly higher mean peak kinematics in defenders compared with midfielders and forwards [74].

Head acceleration in female players who were classified as ‘frequent headers’ compared with those classified as ‘infrequent headers’ was compared in one study [66]. Although group comparison was not possible, qualitatively, it appeared that frequent headers had lower LA, suggesting that technique may play a role in head kinematics [66].

#### Personal Protective Equipment

In total, three studies investigated the effect of protective equipment on head acceleration: protective headgear [83, 100] and clenching with and without a mouthguard [77] (Table 12). Withnall et al. [100] assessed head acceleration in a single subject in free heading and compared this to three different types of headgear, showing that there was no difference in head acceleration. Tierney et al. [83] investigated the use of headgear in both male and female soccer players and found that while there was a small preventative effect for males while wearing the headgear, females had increased LA using both types of headgear.

**Table 12:** Effect of personal protective equipment

Study	Design/participants	Independent variable	Head acceleration measures	Results	Conclusion
Narimatsu et al. [77]	Controlled laboratory study – one-off measurement 11 male high school soccer players Age: 16.8 years	Effect of clenching with and without a mouthguard	Linear acceleration (g)	LA (g) Free heading: 28.4 ± 7.0 Clenching – no mouthguard: 23.9 ± 6.2 Clenching with mouthguard: 21.5 ± 4.6 Significant difference in both clenching without a mouthguard and clenching with the mouthguard compared when compared with free heading (*p* < 0.05) *Calculated mean difference (95% CI) Free heading versus clenching only: 4.5 (− 1.38, 10.38) Free heading versus mouthguard clenching: 6.9 (1.63, 12.17)	• Clenching while performing a header increases masseter and SCM muscle activity and reduces head acceleration • These results were even greater when clenching with a mouthguard
Tierney et al. [83]	Controlled laboratory study – one-off measurement 44 soccer players (29 F, 15 M) Females Age: 19.5 ± 1.8 years Males Age: 20.3 ± 2.9 years	Headgear	Linear acceleration (g)	Women exhibited greater head accelerations versus men when wearing the Head Blast (*t*_1,42_ = 2.89, *p* = 0.006) and Full90 Select (*t*_1,42_ = 3.79, *p* < 0.001). With the control condition, we found no difference (t_1,42_ = 1.42, *p* = 0.164). Specifically, head acceleration in women was 32% greater than in men when wearing the Head Blast (21.52 ± 5.47 g versus 16.27 ± 6.16 g) and was 44% greater than in men when wearing the Full90 Select (21.84 ± 5.34 g versus 15.20 ± 5.83 g). Head acceleration was 10% greater in women than in men during the control condition (20.16 ± 4.12 g versus 18.25 ± 4.48 g)	• Sex differences in head acceleration response associated with wearing soccer headgear • These differences indicate increased LA in women and a protective effect, with reduced acceleration, in men when wearing headgear
Withnall et al. [100]	Controlled laboratory study – one-off measurement One participant (M) Age: 30 years	Headgear	Linear acceleration (g)	For both the 6.4 m/s and 8.2 m/s ball speeds, no significant differences in LA were observed between the bare head and the three different headgears (*p* > 0.05)	• Neither of the three headgears reduced the impact from ball contact • Although only one volunteer represented the human response, tests with the Hybrid III head form showed similar results

*CI* confidence interval, *g* gravitational acceleration, *HA* head acceleration, *LA* linear acceleration, *m/s* metres per second, *SCM* sternocleidomastoid

^*^Indicates where mean difference and 95% CI have been calculated by the authors of this review on the basis of raw data provided in the included studies

Narimatsu et al. [77] investigated the use of clenching with and without a mouthguard during heading. Clenching was associated with significant reductions in head acceleration, both with and without the mouthguard, and the results were more pronounced when clenching with the mouthguard [77].

#### Summary of Key Findings

1. Neck strength and sex affected head acceleration (reduced head acceleration associated with greater neck strength [59, 61, 63, 79, 83] and male sex [48, 62, 63, 71]), although this was not consistent across all studies and all head acceleration variables.
2. Functional neck strength was found to be a more significant predictor of head acceleration compared with isometric neck strength [59].
3. Despite the trend that increased neck strength reduced head acceleration, traditional neck-strengthening interventions did not affect head kinematics [51, 52, 54–56, 60].
4. Neck training interventions that included neuromuscular exercises [17, 59] reduced head acceleration.
5. Ball characteristics affect head acceleration, with reduced ball pressure and ball mass significantly reducing head acceleration [78, 79, 98].
6. Increased ball speed [52, 63, 86, 87, 91, 94, 95, 99] tended to result in greater head acceleration. In line with this, long-range kicks such as goal kicks, corners and punts [45, 69, 70, 72–74, 76, 90] consistently and significantly resulted in greater head acceleration.
7. Playing scenario and head impact location showed mixed results, with some evidence that better heading technique/more experience [67, 88, 101] and forehead impact location [74, 90] lowers head acceleration.
8. There was no conclusive evidence that age or player position affects head acceleration and no consistent difference in head acceleration between practices and games.
9. No studies were identified that investigated sensorimotor factors such as cervical spine proprioception, oculomotor control and vestibular function and their relationship to head acceleration when performing a header.

### Reporting Bias

Reporting bias was assessed in each RoB or quality assessment tool. As such, the item related to reporting bias was marked for each study and was included as part of the overall RoB/quality assessment scoring.

### Certainty of Evidence

The GRADE approach [33, 34] was used as a guide to rate the certainty of evidence and strength of recommendations. Owing to the complexity of the review, with a number of different study designs and RoB/quality assessment tools used, full GRADE assessment was not possible. However, the GRADE approach could still be used as a framework to guide the certainty of evidence assessment. The results of the RoB/quality assessment tools were used to guide the certainty of evidence assessment. Certainty of evidence was graded as very low, low, moderate or high. Certainty of evidence could be rated down owing to risk of bias, inconsistency, indirectness, imprecision and publication bias [34]. The reasons for marking up (having a large magnitude effect, dose–response gradient and the effect of plausible residual confounding [34]) were not relevant for any of the studies/outcomes in this review.

We defined *very low certainty of evidence* across nearly all independent variables investigated in relation to head acceleration. The main reason for this was that only four of the included studies were RCTs, indicating that the starting point for most of the studies in this review using the GRADE approach was ‘low’ certainty of evidence. In total, five of the included studies were considered at ‘serious’ RoB or ‘poor’ quality, which means the certainty of evidence for these specific studies would be ‘very low’. However, pooling the total included studies, with most studies being rated as ‘good’, ‘fair’ or ‘some concerns’, the overall rating at this point was ‘low–very low’ certainty. The certainty of evidence was also marked down owing to indirectness and inconsistency. Indirectness was an issue as there were different population groups, differences in interventions and differences in outcome measures. Inconsistency was marked down as across most of the independent variables, there were mixed results, often without a clear direction of influence to head acceleration. These inconsistencies likely relate to the heterogeneous study designs and methodologies.

The three areas where there was greater consistency in the findings and acceptable–good quality evidence were: ball inflation pressure or mass, neuromuscular training interventions and the type of ball delivery. The certainty of evidence for these three variables was graded higher than the other variables and is considered *low certainty of evidence*. A total of three studies of acceptable–good quality found that reduced inflation pressure and/or mass of the soccer ball reduced head acceleration [78, 79, 98]. In total, two studies of acceptable quality found that including neuromuscular exercises in a neck training programme reduced head acceleration [17, 59]. A total of eight studies (seven rated good quality and one fair quality) all found that head acceleration was increased with longer kicks such as goal kicks, punts and corners [45, 69, 70, 72–74, 76, 90].

Considering the above certainty of evidence discussion, the strength of recommendations for the outcomes of this systematic review would be considered *weak*.

## Discussion

The results of this study demonstrate that a range of intrinsic and extrinsic factors affect head acceleration during a header. With evidence suggesting a link between head acceleration and concussion risk [6, 7] and concerns around the potential long-term effects secondary to repetitive non-concussive impacts involved with heading [8–11], it is important that we understand these factors shown to affect head acceleration. Of note, some papers measured LA only, while others included a rotational measure (RA and/or RV). This is important to consider as LA and RA are thought to be related to different injury mechanisms (intracranial pressure response and strain response respectively), with both contributing to concussion risk [6, 7]. More recently, RV has been suggested as having a stronger correlation to relative brain motion [7].

### Intrinsic Factors

#### Neck Strength

Several studies showed that greater neck strength [59, 61, 63, 79, 83] reduced head acceleration during a header, with functional neck strength a more significant predictor of head acceleration [59]. These findings suggest that players with weaker neck musculature sustained greater head accelerations, possibly putting them at greater risk for sustaining a concussion and having a greater overall head acceleration exposure over their career. However, males had higher neck strength and higher head acceleration for flick-on headers in one study when compared with females, suggesting that stronger muscles do not necessarily reduce head acceleration [92]. One potential limitation is that only one study normalised the strength data to body mass [79]. However, as several studies used one group rather than comparing groups, and most studies in which groups were compared showed no significant difference in body mass between groups, this is not expected to impact the results. Whether neck strength has a direct correlation with head kinematics and concussion risk is yet to be well-understood, but the results of this study suggest that greater neck strength may reduce head acceleration during a purposeful header. This is in conflict with the finding from this review that traditional neck-strengthening interventions did not reduce head acceleration (despite several of these interventions resulting in increased neck strength). With the ballistic nature of a header, more than isometric neck strength alone is needed to affect head acceleration, which is a possible explanation for this difference. Measuring rate of force development of neck muscles is challenging [105] but may be a more relevant outcome in this case compared with isometric neck strength. It is important to note the variability of methods used for assessing neck strength in the included studies. Differences between studies not only in neck strength measurement but also in the methods of measuring head acceleration and the chosen ball delivery method may account for some of the inconsistencies in the relationship between neck strength and head acceleration.

#### Demographics and Anthropometrics

There was agreement that female athletes had greater head acceleration than males across a range of different age groups, from youth to collegiate athletes [48, 62, 63]. A study evaluating postural responses to perturbations identified that males and females use different strategies to stabilise the head in response to impulsive loads [106]. The inference from this study is that females demonstrate increased reliance on neuromuscular activation, while males appear to rely on greater muscle size and strength [106]. This perturbation study offers a potential explanation for the finding in this review that females had greater head acceleration, especially as greater neuromuscular activation may result in more rapid fatigue [106]. Females may also experience greater head accelerations owing to lower head mass and lower neck strength [63], thus creating less effective mass opposing the ball-to-head contact [107]. It should also be noted that a recent study found that females were less likely to be trained in headers compared with males [108], which may lead to poorer technique and execution of the skill.

Results respecting the influence of age on head acceleration were inconsistent. It is thought that one of the reasons younger players sustain greater head accelerations is due to not having fully developed muscular control and comparatively weaker necks than adult populations [107]. Kalichova et al. [91] consolidated this hypothesis showing that as age increased, head acceleration decreased. However, Caccese et al. [62] showed no significant differences between age groups, and Chrisman et al. [44] showed greater head-impact exposure in older athletes. These differences are possibly explained by older athletes being exposed to greater ball velocities and more frequent heading. While there is some conflict in the findings of this review, it is widely accepted that younger athletes should be a group targeted for risk reduction programmes [17, 107] owing to their less developed neck musculature, relative inexperience in heading technique, increased concussion risk and increased recovery times following a concussion [109]. In these formative years, it is important players are safely taught the correct techniques and physically prepared for the task of performing a header. Researchers in Australia are developing a framework (HeaderPrep) to prepare players for heading the ball, which is a positive step forwards for the safety of our youngest players [110]. This is an important consideration, with limitations and/or bans being imposed globally to prevent the number of repetitive ball-to-head impacts [111, 112]. This creates a dilemma in being able to train players to head the ball well from a performance and injury prevention standpoint. A recent study using immersive virtual reality header training showed that the intervention group had significantly improved heading performance and improved perceptions of confidence in heading ability and self-efficacy [113]. Further research is needed in this area, but this result is promising for training the skill of heading without the repetitive head impacts.

#### Header Technique and Impact Location/Playing Experience

One study [88] suggested that better header technique reduced PAA in practices, but not games; however, it should be noted that this study was underpowered. Although heading technique was not specifically investigated, studies investigating the effect of head impact location showed increased acceleration with top and side of the head impacts when compared with a header performed with the forehead. It should be noted that while one study showed no significant difference in head acceleration between impact locations [45], other studies showed significantly higher LA and RA [74] and significantly increased RV [90] in top-of-head impacts (compared with the forehead). Correct heading technique would be considered using the forehead to contact the ball [114]; these studies demonstrate that suboptimal header technique may result in greater head accelerations. This makes a case for ensuring players have adequate header technique prior to starting to head the ball.

Although they did not have a direct objective measure of technique, two studies explored header proficiency and the relationship to head acceleration [67, 101]. Players considered to have ‘poor’ header technique and novice players had greater head acceleration than those with ‘excellent’ technique and greater experience. Another important aspect related to technique is that the player is an active part of the header and meets the ball with purpose, as opposed to the ball hitting them and causing greater displacement of the head. A study using mathematical models based on Newton’s second law showed that the players’ ‘effective mass’ is a major determinant of head acceleration during a header [107]. The effective mass is the mass that opposes the acceleration, and the greater the effective mass of the player, the smaller the acceleration of the head [107]. A greater effective mass was created by having good technique, greater neck strength and stiffness and greater size [107]. This model also supports the findings from this review that players with weaker necks, smaller cylindrical neck volume and female and younger athletes had increased head acceleration.

### Extrinsic Factors

#### Neck Training Interventions

With the correlation identified between increased neck strength and decreased head acceleration, authors from several studies hypothesised that a neck training intervention would reduce head acceleration. A total of six studies [51, 52, 54–56, 60] found no changes in head acceleration following a traditional neck strengthening intervention, despite a number of these studies showing improved neck strength. The two programmes [17, 59] that were able to show a reduction in head acceleration both included neuromuscular training exercises. Benefits of performing isometric and/or isotonic exercises with the aim of reducing concussion risk in contact sports have been discussed for several years, and neck strengthening programmes have previously been thought of as concussion prevention tools. More recently, there has been growing evidence that isometric and isotonic strengthening are not enough, and recent reviews [18, 115] have questioned the reliability and sensitivity of the strength measures used in the Collins et al., 2014 [116] study which suggested benefits of neck strength for reducing concussion risk. A number of review papers published in recent years [15, 16, 18, 115] have highlighted that traditional neck strengthening alone may not be an effective concussion prevention tool and that interventions need to target improving neuromuscular control, neck muscle activation, dynamic strength and rate of force development. Since players require quick and effective recruitment of neck musculature when heading, the need for an appropriate motor strategy is clear, and neuromuscular exercises should be incorporated when the training aims to influence controlling the head in a ballistic task such as heading the ball.

#### Fatigue Protocols

A total of three [56–58] of the four studies assessing head acceleration following fatigue protocols rejected the hypothesis that head acceleration would increase following a fatigue protocol, with one showing no change and two showing a slight reduction in head acceleration. It was hypothesised that inducing fatigue would cause greater head acceleration during a header, potentially owing to reduced head control and poorer technique. It should be noted that these three studies were all at moderate RoB, used a core fatigue protocol and used a hanging pendulum device for the header protocols. The one other study assessing the effect of a fatigue protocol [53] did show a significant increase in LA (and a non-significant increase in RA) following a more generalised fatigue protocol of both cardiovascular and strength exercises. This fatigue protocol would be considered more specific to expected fatigue in a soccer training session or game, but it should also be noted this study was at serious risk of bias owing to confounding.

#### Ball Speed, Ball Delivery and Ball Characteristics

Reduced-mass and pressure balls appear to have a significant effect on reducing head acceleration [78, 79, 98]. With these findings in mind, it seems sensible to consider both inflation pressure and ball weight and reduce these or ensure balls are at the lowest end of manufacturer pressure recommendations to reduce head acceleration during headers. It is of particular importance in younger players, with a smaller effective mass as described above, that they are playing with the correct size/weight/inflation balls. The effect of ball velocity was more mixed but generally showed that increasing velocity led to higher acceleration values, which was often more significant for linear acceleration [52, 63, 87, 91, 94, 95, 99]. Head acceleration increased with long range kicks such as goal kicks, corners and punts [45, 69, 70, 72–74, 76, 90]. Some literature calls for bans on heading balls from long range kicks such as goal kicks and punts from the keepers’ hands, especially in ‘high-risk’ populations such as children/youth and females [70]. Conversely, other studies note that headers from punts and goal kicks are uncommon in female youth players. Heading is more common from passes in the air or throw-ins, suggesting that if passing the ball was limited to along the ground, the number of headers could be reduced by as much as 41% [90]. While this may not reduce the highest magnitude headers, it would affect the overall head-impact exposure. Both measures could be useful to lower the rate of headers and to remove headers with the greatest acceleration. Considering overall heading exposure over a playing career, simple steps even prior to any heading bans such as encouraging short corners, short goal kicks and playing out from the back (therefore reducing the number of long distance and high velocity balls) could be beneficial. These recommendations (and those in relation to ball pressure and neuromuscular exercises) are in line with a recent narrative review [114] which also suggested that such strategies are worthwhile to implement while further research is ongoing.

#### Playing Scenario, Player Position and Header Type

The literature was inconclusive for any consistent significant differences in head acceleration between either player position or in games versus practices. There was limited evidence that defenders may sustain higher accelerations compared with midfielders and forwards [74]; this may relate to the fact they are more likely to head long-range kicks from corners and goal kicks. With there being no consistent difference in head acceleration between games and practices, limiting heading during practices, particularly repetitive headers from long-range kicks (as would happen at a set-piece training), could be a useful way to reduce overall head acceleration exposure.

#### Personal Protective Equipment

In agreement with a recent systematic review including soccer and rugby players which concluded that there was no protective effect of wearing headgear on concussion risk [117], this review found that wearing headgear did not provide any benefit, with no reduction in head acceleration. As previously discussed, the use of headgear may be problematic as it has no benefit from a concussion-risk standpoint [117], but it can often provide players with a false sense of security or feeling of being protected. While only investigated by one study, there is some evidence that jaw clenching, particularly clenching with a mouthguard in place, may reduce head acceleration during a header through increased masseter and sternocleidomastoid (SCM) activation [77]. There was increased masseter and SCM activity with the clenching conditions in this study [77], which suggests that increased muscle activation and strength of these muscles could reduce head acceleration. While mouthguards are not routinely worn by soccer players, further research into the relationship between mouthguard use in soccer and concussion risk would be beneficial.

Given the number of studies eligible for inclusion in this review (and the number of new studies (nine) included following re-running the search strategy), it is evident that there is growing interest in the head acceleration involved with performing a purposeful header. Much of this interest stems from the debate around the safety of performing headers in relation to concussion risk and, more so, in relation to the potential long-term effects on brain health of repetitive ball-to-head impacts over a playing career. This review highlights factors that are known to affect head acceleration and proposes several measures that can be taken to reduce head acceleration with the aim of improving the safety of purposeful headers. In addition, the lack of evidence surrounding a wider range of sensorimotor outcomes that are likely to affect head acceleration is highlighted.

### Methodological Considerations

While conducted in accordance with best practice guidelines (PRISMA [22] and JBI [23]), this systematic review is not without limitations. Large variability in the study design, methodology and measurement of head acceleration within included studies meant that study results were too heterogeneous to synthesise, and pooling of data via meta-analysis (and full GRADE assessment) was not possible. Differences in study quality and substantial variability in study methods mean that the results of some studies must be interpreted with care. Due to the variability in studies assessing head kinematics, an expert group created methodological guidelines to reduce the risk of bias and improve the consistency, rigor and quality of studies using head kinematic devices [118]. One limitation of this review is that, for 70% of studies included, the acceleration data were not transformed to the head-centre of gravity (or other suitable frame of reference), which is a checklist point in the guidelines [118]. This made pooling of data implausible and comparison of results between studies challenging [104]. However, as this review was investigating the relationship between variables and head acceleration in each paper and not comparing numerical acceleration data across papers (but rather comparing the relationships found), the lack of transformation of the data had less of an effect on this review and is not a major limitation. It does, however, mean that the acceleration data found in individual studies should not be compared with each other. It is important to consider that the methodological guidelines discussed above [118] were published in 2022, and 75% of the studies included in this review were published before 2022. It was noted that the more recent studies included more commonly transformed data to the head-centre of gravity, suggesting that these guidelines are being utilised. Along with the variability in the measurement of head kinematics, there was also large variation in the heading trial methods (ball speed, method of delivery, number of headers performed). This is important to ensure participant safety and that heading trials are representative of game scenario headers. Recently, guidelines for studies in acute heading research have been published [119]. The application of these guidelines for future heading studies, along with continued implementation of the head kinematic methodology guidelines [118], will improve the methodological quality of heading studies.

### Implications of Results

The implications of this review are twofold. First, areas highlighted in relation to ball characteristics/playing style that are shown to increase head acceleration (increased ball velocity, long range kicks, higher ball weight and inflation) should be noted. Measures could be considered to potentially reduce head-impact exposure in ways such as: ensuring correct ball inflation/weight at the lower end of the manufacturer’s pressure/weight recommendations and limiting the number of headers performed from long range kicks, particularly in higher risk groups such as children/adolescents and female players. Additionally, studies suggest that neuromuscular exercises need to be included in training programmes that are targeting concussion prevention and/or altering head kinematics. This review highlights that traditional neck strengthening did not affect head acceleration, while programmes with neuromuscular exercises and/or dynamic training were more likely to reduce head acceleration. This shows the importance of designing training interventions specific to the task of performing a header.

### Directions for Future Research

Researchers should continue the work exploring neuromuscular neck exercises, which is underway [17], following the lack of translation from peak isometric strength gains to reducing head kinematics with traditional neck-strengthening interventions. There may be a required level of neck strength, but with the focus correctly shifting to neuromuscular control and dynamic head stability, there is a need to consider other factors that contribute to head and neck control in heading the ball. Factors such as cervical spine proprioception, oculomotor control, vestibular function and balance all have a role to play in position sense, ball tracking and head control. A review [120] assessing visual and sensorimotor performance in relation to concussion risk highlighted the lack of research in this area, despite it being an area that warrants further investigation, with potential effects of visual and oculomotor performance and anticipation on the frequency and severity of head impact in helmeted sports. Such factors listed above have not yet been investigated in relation to head acceleration during a purposeful header. Understanding whether these modifiable and intrinsic factors influence head acceleration during a header would mean training interventions could be trialled. This would be a useful addition to the body of work investigating neuromuscular exercises, as well as to the evidence relating to extrinsic factors, such as changes to ball characteristics/playing style, as discussed.

## Conclusions

This systematic review provides a comprehensive overview of the current evidence base surrounding factors that influence head acceleration during a purposeful header. While there are several areas where evidence is conflicting and study quality is mixed, there are some extrinsic factors related to the game (ball pressure and mass, ball delivery method and ball speed) and individual factors related to the player (neck strength, sex and heading technique) that influence head acceleration during heading. Implementation of neuromuscular exercises reduced head acceleration. There is a lack of evidence for a broader range of modifiable potential risk factors and in understanding how other sensorimotor factors influence head acceleration during a header. Further research into factors such as vestibular and oculomotor function will provide information to assist the current research base aiming to improve the safety of headers.

## Supplementary Information

Below is the link to the electronic supplementary material.

Supplementary file1 (DOCX 104 KB)

## Declarations

### Availability of data and material

The full data extracted for this review are held by the lead author. The template data collection form is included in the appendices. Results and methods tables in the manuscript and appendices show the key data extracted and the data used for analysis.
