# Supplementary material for: What Factors Influence Head Acceleration During a Purposeful Header in Soccer Players? A Systematic Review
Source: Sports Med. 2025 Apr 24;55(7):1677–741. doi: 10.1007/s40279-025-02209-2 (PMC12296814; doi:10.1007/s40279-025-02209-2)
Supplement: Supplementary file 1 — Supplementary file1 (DOCX 104 KB) [file 40279_2025_2209_MOESM1_ESM.docx]

**Appendix I** Search strategy for each database/search engine used

*Scopus: 1202 results*

( TITLE-ABS-KEY ( "head acceleration*" OR "head impact*" OR "head impact magnitude" OR "head kinematic*" OR "head movement*" OR "head stabil*" OR "head motion" OR "head velocity" OR header* OR heading OR "purposeful header*" OR "purposeful heading" ) AND TITLE-ABS-KEY ( soccer OR football ) )

*CINAHL: 425 results*

( "head acceleration*" or "head impact*" or "head impact magnitude" or "head kinematic*" or "head movement*" or "head stabil*" or "head motion" or "head velocity" or header* or heading or "purposeful header*" or "purposeful heading" ) AND ( soccer OR football )

*EMBASE: 1155 results*

1 ("head acceleration*" or "head impact*" or "head impact magnitude" or "head kinematic*" or "head movement*" or "head stabil*" or "head motion" or "head velocity" or header* or heading or "purposeful header*" or "purposeful heading").mp. [mp=title, abstract, heading word, drug trade name, original title, device manufacturer, drug manufacturer, device trade name, keyword heading word, floating subheading word, candidate term word] 29483

2 (soccer or football).mp. [mp=title, abstract, heading word, drug trade name, original title, device manufacturer, drug manufacturer, device trade name, keyword heading word, floating subheading word, candidate term word] 28302

3 1 and 2 1155

*MEDLINE: 767 results*

1 ("head acceleration*" or "head impact*" or "head impact magnitude" or "head kinematic*" or "head movement*" or "head stabil*" or "head motion" or "head velocity" or header* or heading or "purposeful header*" or "purposeful heading").mp. [mp=title, book title, abstract, original title, name of substance word, subject heading word, floating sub-heading word, keyword heading word, organism supplementary concept word, protocol supplementary concept word, rare disease supplementary concept word, unique identifier, synonyms] 16818

2 (soccer or football).mp. [mp=title, book title, abstract, original title, name of substance word, subject heading word, floating sub-heading word, keyword heading word, organism supplementary concept word, protocol supplementary concept word, rare disease supplementary concept word, unique identifier, synonyms] 20230

3 1 and 2 767

*Web of Science: 4914 results*

"head acceleration*" OR "head impact*" OR "head impact magnitude" OR "head kinematic*" OR "head movement*" OR "head stabil*" OR "head motion" OR "head velocity" OR header* OR heading OR "purposeful header*" OR "purposeful heading" AND soccer OR football

*SPORTDiscus: 1105 results*

("head acceleration*" or "head impact*" or "head impact magnitude" or "head kinematic*" or "head movement*" or "head stabil*" or "head motion" or "head velocity" or header* or heading or "purposeful header*" or "purposeful heading") AND ( soccer OR football )

*ClinicalKey: 22 results*

"head acceleration" OR "head impact*" OR "head kinematic*" OR header* OR heading AND soccer OR football

*Google Scholar: 1350 results*

978 results able to be exported for screening as per Google Scholars limit on search results

"head acceleration*" OR "head impact*" OR "head impact magnitude" OR "head kinematic*" OR "head movement*" OR "head stabil*" OR "head motion" OR "head velocity" AND header* OR "purposeful header*" OR heading* OR "purposeful heading*" AND soccer OR football

*MedNar: 779 ‘top results’*

‘Patents’ results excluded – 753 results exported for screening

"head acceleration*" OR "head impact*" OR "head impact magnitude" OR "head kinematic*" OR "head movement*" OR "head stabil*" OR "head motion" OR "head velocity" OR header* OR "purposeful header*" OR heading* OR "purposeful heading*" AND soccer OR football

*TRIP Pro: 4668 total results*

"head acceleration*" OR "head impact*" OR "head impact magnitude" OR "head kinematic*" OR "head movement*" OR "head stabil*" OR "head motion" OR "head velocity" OR header* OR "purposeful header*" OR heading* OR "purposeful heading*" AND soccer OR football

*Filters applied: results suitable for screening: 2335*

Categories removed from search: guidelines, regulatory guidance, clinical Q&A, ongoing systematic reviews, ongoing clinical trials, patient information leaflets, blogs, eTextbooks, medical images and videos.
Remaining categories searched: systematic reviews, evidence-based synopses, key primary research, controlled trials, primary research.

*Clinical Trial Sites*

*ANZCTR* – individual searches for ‘head acceleration’, ‘header’, ‘soccer/football’ – all results screened by RB and none were suitable for inclusion

*EU Clinical Trials Register* - individual searches for ‘head acceleration’, ‘header’, ‘heading’, ‘soccer/football’ – all results screened by RB and none were suitable for inclusion

*International Clinical Trials Registry Platform (ICTRP) hosted by WHO* - individual searches for ‘head acceleration’, ‘header’, ‘soccer/football’ – all results screened by RB and none were suitable for inclusion.

*Clinical Trials*– individual searches for ‘head acceleration’, ‘header’, ‘soccer/football’ – all results screened by RB and none were suitable for inclusion.

**Appendix II** Data extraction tool

|  |  |
| --- | --- |
| **STUDY DETAILS** |  |
| Citation Details |  |
| **STUDY METHODS/CHARACTERISTICS** |  |
| Study Design |  |
| Setting |  |
| Participants (age, sex, playing level) |  |
| Recruitment Procedures |  |
| Follow-up/Study Duration |  |
| Frame of Reference |  |
| Exposure (Heading Method) |  |
| Influencing Factor(s) Investigated |  |
| **OUTCOMES** |  |
| Primary Outcome |  |
| Measurement Method of Head Acceleration (HA) |  |
| **DATA ANALYSIS** |  |
| Data Analysis Methods |  |
| **STUDY RESULTS** |  |
| Resulting HA |  |
| Relationship Between HA and Influencing Factor |  |
| Measures of Effect Size |  |
| P value and 95% Confidence Intervals |  |
| **REVIEWER COMMENTS** |  |
| Relevant Comments Related to Review Question |  |

Review Title: What factors influence head acceleration during a purposeful header in soccer players? A systematic review.

Aim: To review the literature to investigate what variables (intrinsic and extrinsic) influence head acceleration during a purposeful header in soccer players.

**Appendix III:** Study designs and risk of bias/quality assessment tools

| **Study Design** | **Definition** | **Tool Used** |
| --- | --- | --- |
| Cohort studies | Observational studies that followed participants over a period (commonly a soccer season). Cohort studies in this review were often those measuring head acceleration during live trainings and games during a soccer season. | Modified Downs and Black |
| Cross-sectional studies | Observational studies measuring a single variable at one point in time. | AXIS |
| Non-randomised trials of interventions | Prospective studies where an intervention was implemented, but participants were not randomised into groups. In this review this was often as one soccer team would be the intervention group and another team the control group. | ROBINS-I |
| One-off measurement involving different variables | Observational studies measuring multiple different variables, usually at one point in time or over a short time period. For this review, this was often studies measuring different ball speeds/inflation pressures/types of headers etc. | Modified Downs and Black |
| Randomised control trials | Prospective studies where an intervention was tested against a control with participants randomised into groups. | RoB2 |

| **Appendix IV** Risk of bias/quality assessment tools | |
| --- | --- |
| ROBINS-I | |
| **Signaling Questions** | **Response Options** |
| **Bias due to confounding** | |
| 1.1 Is there potential for confounding of the effect of intervention in this study?  **If N/PN to 1.1:** the study can be considered to be at low risk of bias due to confounding and no further signalling questions need be considered  **If Y/PY to 1.1**: determine whether there is a need to assess time-varying confounding: | Y / PY / PN / N |
| 1.2. Was the analysis based on splitting participants’ follow up time according to intervention received?  **If N/PN**, answer questions relating to baseline confounding (1.4 to 1.6)  **If Y/PY**, go to question 1.3. | NA / Y / PY / PN / N / NI |
| 1.3. Were intervention discontinuations or switches likely to be related to factors that are prognostic for the outcome?  **If N/PN**, answer questions relating to baseline confounding (1.4 to 1.6)  **If Y/PY**, answer questions relating to both baseline and time-varying confounding (1.7 and 1.8) | NA / Y / PY / PN / N / NI |
| **Questions relating to baseline confounding only** | |
| 1.4. Did the authors use an appropriate analysis method that controlled for all the important confounding domains? | NA / Y / PY / PN / N / NI |
| 1.5. **If Y/PY to 1.4**: Were confounding domains that were controlled for measured validly and reliably by the variables available in this study? | NA / Y / PY / PN / N / NI |
| 1.6. Did the authors control for any post-intervention variables that could have been affected by the intervention? | NA / Y / PY / PN / N / NI |
| **Questions relating to baseline and time-varying confounding** | |
| 1.7. Did the authors use an appropriate analysis method that controlled for all the important confounding domains and for time-varying confounding? | NA / Y / PY / PN / N / NI |
| 1.8. **If Y/PY to 1.7**: Were confounding domains that were controlled for measured validly and reliably by the variables available in this study? | NA / Y / PY / PN / N / NI |
| **Risk of bias judgement** | Low / Moderate / Serious / Critical / NI |
| Optional: What is the predicted direction of bias due to confounding? | Favours experimental / Favours comparator / Unpredictable |
| **Bias in selection of participants into the study** | |
| 2.1. Was selection of participants into the study (or into the analysis) based on participant characteristics observed after the start of intervention?  **If N/PN to 2.1:** go to 2.4 | Y / PY / PN / N / NI |
| 2.2. **If Y/PY to 2.1**: Were the post-intervention variables that influenced selection likely to be associated with intervention?  2.3 **If Y/PY to 2.2**: Were the post-intervention variables that influenced selection likely to be influenced by the outcome or a cause of the outcome? | NA / Y / PY / PN / N / NI  NA / Y / PY / PN / N / NI |
| 2.4. Do start of follow-up and start of intervention coincide for most participants? | Y / PY / PN / N / NI |
| 2.5. **If Y/PY to 2.2 and 2.3, or N/PN to 2.4**: Were adjustment techniques used that are likely to correct for the presence of selection biases? | NA / Y / PY / PN / N / NI |
| **Risk of bias judgement** | Low / Moderate / Serious / Critical / NI |
| Optional: What is the predicted direction of bias due to selection of participants into the study? | Favours experimental / Favours comparator / Towards null /Away from null / Unpredictable |
| **Bias in classification of interventions** | |
| 3.1 Were intervention groups clearly defined? | Y / PY / PN / N / NI |
| 3.2 Was the information used to define intervention groups recorded at the start of the intervention? | Y / PY / PN / N / NI |
| 3.3 Could classification of intervention status have been affected by knowledge of the outcome or risk of the outcome? | Y / PY / PN / N / NI |
| **Risk of bias judgement** | Low / Moderate / Serious / Critical / NI |
| Optional: What is the predicted direction of bias due to classification of interventions? | Favours experimental / Favours comparator / Towards null /Away from null / Unpredictable |
| **Bias due to deviations from intended interventions** | |
| **If your aim for this study is to assess the effect of assignment to intervention, answer questions 4.1 and 4.2** | |
| 4.1. Were there deviations from the intended intervention beyond what would be expected in usual practice? | Y / PY / PN / N / NI |
| 4.2. **If Y/PY to 4.1**: Were these deviations from intended intervention unbalanced between groups *and* likely to have affected the outcome? | NA / Y / PY / PN / N / NI |
| **If your aim for this study is to assess the effect of starting and adhering to intervention, answer questions 4.3 to 4.6** | |
| 4.3. Were important co-interventions balanced across intervention groups? | Y / PY / PN / N / NI |
| 4.4. Was the intervention implemented successfully for most participants? | Y / PY / PN / N / NI |
| 4.5. Did study participants adhere to the assigned intervention regimen? | Y / PY / PN / N / NI |
| 4.6. **If N/PN to 4.3, 4.4 or 4.5**: Was an appropriate analysis used to estimate the effect of starting and adhering to the intervention? | NA / Y / PY / PN / N / NI |
| **Risk of bias judgement** | Low / Moderate / Serious / Critical / NI |
| Optional: What is the predicted direction of bias due to deviations from the intended interventions? | Favours experimental / Favours comparator / Towards null /Away from null / Unpredictable |
| **Bias due to missing data** | |
| 5.1 Were outcome data available for all, or nearly all, participants? | Y / PY / PN / N / NI |
| 5.2 Were participants excluded due to missing data on intervention status? | Y / PY / PN / N / NI |
| 5.3 Were participants excluded due to missing data on other variables needed for the analysis? | Y / PY / PN / N / NI |
| 5.4 **If PN/N to 5.1, or Y/PY to 5.2 or 5.3**: Are the proportion of participants and reasons for missing data similar across interventions? | NA / Y / PY / PN / N / NI |
| 5.5 **If PN/N to 5.1, or Y/PY to 5.2 or 5.3**: Is there evidence that results were robust to the presence of missing data? | NA / Y / PY / PN / N / NI |
| **Risk of bias judgement** | Low / Moderate / Serious / Critical / NI |
| Optional: What is the predicted direction of bias due to missing data? | Favours experimental / Favours comparator / Towards null /Away from null / Unpredictable |
| **Bias in measurement of outcomes** | |
| 6.1 Could the outcome measure have been influenced by knowledge of the intervention received? | Y / PY / PN / N / NI |
| 6.2 Were outcome assessors aware of the intervention received by study participants? | Y / PY / PN / N / NI |
| 6.3 Were the methods of outcome assessment comparable across intervention groups? | Y / PY / PN / N / NI |
| 6.4 Were any systematic errors in measurement of the outcome related to intervention received? | Y / PY / PN / N / NI |
| **Risk of bias judgement** | Low / Moderate / Serious / Critical / NI |
| Optional: What is the predicted direction of bias due to measurement of outcomes? | Favours experimental / Favours comparator / Towards null /Away from null / Unpredictable |
| **Bias in selection of the reported result** | |
| Is the reported effect estimate likely to be selected, on the basis of the results, from... | Y / PY / PN / N / NI |
| 7.1. ... multiple outcome *measurements* within the outcome domain? | Y / PY / PN / N / NI |
| 7.2 ... multiple *analyses* of the intervention-outcome relationship? | Y / PY / PN / N / NI |
| 7.3 ... different *subgroups*? | Low / Moderate / Serious / Critical / NI |
| **Risk of bias judgement** | Favours experimental / Favours comparator / Towards null /Away from null / Unpredictable |
| Optional: What is the predicted direction of bias due to selection of the reported result? |  |
| **Overall bias** | |
| **Risk of bias judgement** | Low / Moderate / Serious / Critical / NI |
| Optional: What is the overall predicted direction of bias for this outcome? | Favours experimental / Favours comparator / Towards null /Away from null / Unpredictable |

| RoB2 | |
| --- | --- |
| **Signalling questions** | **Response options** |
| **Domain 1: Risk of bias arising from the randomization process** | |
| 1.1 Was the allocation sequence random? | Y / PY / PN / N / NI |
| 1.2 Was the allocation sequence concealed until participants were enrolled and assigned to interventions? | Y / PY / PN / N / NI |
| 1.3 Did baseline differences between intervention groups suggest a problem with the randomization process? | Y / PY / PN / N / NI |
| **Risk-of-bias judgement** | Low / High / Some concerns |
| Optional: What is the predicted direction of bias arising from the randomization process? | NA / Favours experimental / Favours comparator / Towards null /Away from null / Unpredictable |
| **Domain 2: Risk of bias due to deviations from the intended interventions (*effect of assignment to intervention*)** | |
| 2.1. Were participants aware of their assigned intervention during the trial? | Y / PY / PN / N / NI |
| 2.2. Were carers and people delivering the interventions aware of participants' assigned intervention during the trial? | Y / PY / PN / N / NI |
| 2.3. If Y/PY/NI to 2.1 or 2.2: Were there deviations from the intended intervention that arose because of the trial context? | NA / Y / PY / PN / N / NI |
| 2.4 If Y/PY to 2.3: Were these deviations likely to have affected the outcome? | NA / Y / PY / PN / N / NI |
| 2.5. If Y/PY/NI to 2.4: Were these deviations from intended intervention balanced between groups? | NA / Y / PY / PN / N / NI |
| 2.6 Was an appropriate analysis used to estimate the effect of assignment to intervention? | Y / PY / PN / N / NI |
| 2.7 If N/PN/NI to 2.6: Was there potential for a substantial impact (on the result) of the failure to analyse participants in the group to which they were randomized? | NA / Y / PY / PN / N / NI |
| **Risk-of-bias judgement** | Low / High / Some concerns |
| Optional: What is the predicted direction of bias due to deviations from intended interventions? | NA / Favours experimental / Favours comparator / Towards null /Away from null / Unpredictable |
| **Domain 2: Risk of bias due to deviations from the intended interventions (*effect of adhering to intervention*)** | |
| 2.1. Were participants aware of their assigned intervention during the trial? | Y / PY / PN / N / NI |
| 2.2. Were carers and people delivering the interventions aware of participants' assigned intervention during the trial? | Y / PY / PN / N / NI |
| 2.3. [If applicable:] If Y/PY/NI to 2.1 or 2.2: Were important non-protocol interventions balanced across intervention groups? | NA / Y / PY / PN / N / NI |
| 2.4. [If applicable:] Were there failures in implementing the intervention that could have affected the outcome? | NA / Y / PY / PN / N / NI |
| 2.5. [If applicable:] Was there non-adherence to the assigned intervention regimen that could have affected participants’ outcomes? | NA / Y / PY / PN / N / NI |
| 2.6. If N/PN/NI to 2.3, or Y/PY/NI to 2.4 or 2.5: Was an appropriate analysis used to estimate the effect of adhering to the intervention? | NA / Y / PY / PN / N / NI |
| **Risk-of-bias judgement** | Low / High / Some concerns |
| Optional: What is the predicted direction of bias due to deviations from intended interventions? | NA / Favours experimental / Favours comparator / Towards null /Away from null / Unpredictable |
| **Domain 3: Missing outcome data** | |
| 3.1 Were data for this outcome available for all, or nearly all, participants randomized? | Y / PY / PN / N / NI |
| 3.2 If N/PN/NI to 3.1: Is there evidence that the result was not biased by missing outcome data? | NA / Y / PY / PN / N |
| 3.3 If N/PN to 3.2: Could missingness in the outcome depend on its true value? | NA / Y / PY / PN / N / NI |
| 3.4 If Y/PY/NI to 3.3: Is it likely that missingness in the outcome depended on its true value? | NA / Y / PY / PN / N / NI |
| **Risk-of-bias judgement** | Low / High / Some concerns |
| Optional: What is the predicted direction of bias due to missing outcome data? | NA / Favours experimental / Favours comparator / Towards null /Away from null / Unpredictable |
| **Domain 4: Risk of bias in measurement of the outcome** | |
| 4.1 Was the method of measuring the outcome inappropriate? | Y / PY / PN / N / NI |
| 4.2 Could measurement or ascertainment of the outcome have differed between intervention groups? | Y / PY / PN / N / NI |
| 4.3 If N/PN/NI to 4.1 and 4.2: Were outcome assessors aware of the intervention received by study participants? | NA / Y / PY / PN / N / NI |
| 4.4 If Y/PY/NI to 4.3: Could assessment of the outcome have been influenced by knowledge of intervention received? | NA / Y / PY / PN / N / NI |
| 4.5 If Y/PY/NI to 4.4: Is it likely that assessment of the outcome was influenced by knowledge of intervention received? | NA / Y / PY / PN / N / NI |
| **Risk-of-bias judgement** | Low / High / Some concerns |
| Optional: What is the predicted direction of bias in measurement of the outcome? | NA / Favours experimental / Favours comparator / Towards null /Away from null / Unpredictable |
| **Domain 5: Risk of bias in selection of the reported result** | |
| 5.1 Were the data that produced this result analysed in accordance with a pre-specified analysis plan that was finalized before unblinded outcome data were available for analysis? | Y / PY / PN / N / NI |
| Is the numerical result being assessed likely to have been selected, on the basis of the results, from... | |
| 5.2. ... multiple eligible outcome measurements (e.g. scales, definitions, time points) within the outcome domain? | Y / PY / PN / N / NI |
| 5.3 ... multiple eligible analyses of the data? | Y / PY / PN / N / NI |
| **Risk-of-bias judgement** | Low / High / Some concerns |
| Optional: What is the predicted direction of bias due to selection of the reported result? | NA / Favours experimental / Favours comparator / Towards null /Away from null / Unpredictable |
| **Overall risk of bias** | |
| **Risk-of-bias judgement** | Low / High / Some concerns |
| Optional: What is the overall predicted direction of bias for this outcome? | NA / Favours experimental / Favours comparator / Towards null /Away from null / Unpredictable |

**Appendix V** Methods Table

| Study | Design/Participants | Independent Variable(s) | Methods | Head acceleration measures | Frame of Reference |
| --- | --- | --- | --- | --- | --- |
| Austin et al. [85] | Controlled laboratory study - one off measurement 12 male participants  Age: 24 ± 3.2 years | Ball velocity 5 m/s versus 8 m/s  Ball inflation pressure 6 psi versus 8 psi | Ball delivery - underhand throw from 4.5 m away (low velocity) and 4.5 m away and 4 m drop height (high velocity) Ball speed(s) and characteristics – 5 m/sand 8 m/s  Header type(s) - headed back to the throwers feet Number of headers - 20 Data collection - three-dimensional motion capture sampling at 1000 Hz (Vicon T40s, Vicon Motion Systems, UK). | Peak linear acceleration (g) Peak angular acceleration (rad/s^2^) | Yes - head segment centre of mass. |
| Barnes-Wood et al. [86] | Controlled laboratory study - one off measurement 7 male amateur soccer players Age: 20.1 ± 1 years | Ball velocity/length of ball delivery:  8 m/s short  13 m/s medium  16m/s long | Ball delivery - Globus EuroGoal 3000 ball launcher from short, medium and long distances Ball speed(s) and characteristics – 8 m/s , 13 m/s and 16 m/s. Adidas Champions League 21/22 soccer ball inflated to 0.8 bar, mass = 434.17 g ± 0.35 g. Header type(s) - return the ball back towards the delivery location, using the frontal portion of head Number of headers - 3 short, 3 medium and 4 long passes Data collection – custom fit iMG (Opro; Hertfordshire, UK). The iMG main board comprised: a 3-axis accelerometer (H3LIS331DL; STMicroelectronics, Genova, Switzerland), sampling at 1 kHz (± 200 g, 16-bit resolution); a 3-axis gyroscope (LSM9DS1; STMicroelectronics, Genova, Switzerland)) sampling at 0.952 kHz (± 35 rad s−1, 16-bit resolution); and an additional accelerometer with a focussed bandwidth (0.5–1 kHz). The iMG was programmed to trigger data collection when a threshold of 5 g was met in any of the X, Y or Z directions for linear acceleration. Once the threshold was achieved, a 104 ms window of linear and angular acceleration data was collected. All iMG data was manually cross-checked against highspeed video. | Peak linear acceleration (g) Peak angular acceleration (rad/s^2^)  Peak angular velocity  (rad/s) | Yes – head centre of gravity. |
| Becker et al. [57] | Non-randomised study of interventions 41 amateur soccer players divided into two independent subgroups. 1. Muscular activity (n= 12) Age: 23.6 ± 4.2 years 2. Kinematics and dynamics (n=29)  Age: 23.7 ± 2.8 years | Effect of fatigue of the core stabilising muscles | Ball delivery - stationary pendulum header (Derbystar model: Swing) Ball speed(s) and characteristics - size 5 ball, diameter 22 cm Header type(s) - from a standing position, jumping with both legs and heading the ball as forcefully as possible in a horizontal forward direction Number of headers - 6 Data collection - a 3D accelerometer (Noraxon, Scottsdale, AZ, USA) was attached to the occipital area. | Head acceleration (g) | Not reported |
| Becker et al. [58] | Non-randomised study of interventions 68 soccer players, 4th-9th division and active recreational players  Age: 21.5 ± 3.8 years | Header type: standing, jumping, running Effect of fatigue of the core stabilising muscles | Ball delivery - stationary pendulum header (DerbystarGoch, Germany; model: Swing) Ball speed(s) and characteristics - size 5 ball, diameter 22 cm Header type(s) - standing, jumping and running headers with the ball headed as powerfully as possible in a horizontal forward direction Number of headers - not reported  Data collection - DTS 3D accelerometer (Noraxon USA Inc., Scottsdale, AZ, USA; 22 × 16 × 7 mm, 2.8 gm) attached to the occipital area. It was attached using an individually adjustable rubber band (Noraxon USA Inc.; 1000 × 35 mm). | Linear head acceleration (g) | Not reported |
| Becker et al. [56] | Non-randomised study of interventions 33 male soccer players Age: 20.3 ± 3.6 years  Two intervention groups IG1 -adult team (n=11) IG2 - youth team (n=9) Control group: CG (n=13) | 6-week neck strength training for the neck flexors and extensors Effect of fatigue protocol | Ball delivery - stationary pendulum header (Derbystar, model: Swing) Ball speed(s) and characteristics - size 5 ball, 22 cm diameter  Header type(s) - standing, jumping, running, post-jumping, post-running with the ball headed horizontally to the front as hard as possible Number of headers - not reported other than 3 headers of each variant before testing to reduce learning effect Data collection - telemetric DTS 3D accelerometer (Noraxon, Scottdale, USA; frequency: 1500 Hz, filter: Lowpass 500 Hz) fixed in the occipital area of the head. An individually adjustable rubber band (Noraxon, Scottdale, USA; size: 1000x35 mm) was used for attachment and wrapped at least two times over the accelerometer and head to prevent motion artifacts in the best possible way. | Head acceleration (g) | Not reported |
| Becker et al. [68] | Controlled laboratory study - one off measurement 60 male active soccer players Age: 18.9 ± 4.0 years | Header type: standing, jumping, running Head-neck torso alignment | Ball delivery - stationary pendulum header (Derbystar, model: Swing) Ball speed(s) and characteristics - size 5 ball, diameter 22 cm Header type(s) - standing, jumping and running headers with the ball headed as powerfully as possible in a horizontal forward direction Number of headers - 18 Data collection - telemetric DTS 3D accelerometer (Noraxon, Scottdale, USA; frequency: 1500 Hz, lowpass filter: 500 Hz; operational range: 6.25 G per axis). It was attached to the occipital area of the head with an individually adjustable rubber band (Noraxon, Scottdale, USA; size: 1000 x 35 mm). | Linear head acceleration (g) | Not reported |
| Bretzin et al. [63] | Cross-sectional 13 NCAA Division I soccer players (8 F, 5 M) | Ball speed 25 mph versus 40 mph Sex Anthropometrics Neck strength | Ball delivery - JUGS machine (JUGS Sports International) Ball speed(s) and characteristics - 25 mph and 40 mph; size 5, 450 g soccer ball, inflated to 8 psi Header type(s) - not reported Number of headers - 5 Data collection - head impact kinematic measurements were recorded with an accelerometer (Gforcetracker Inc). During testing, sensors were positioned on the back of the participants’ head using prewrap and tape. Impacts measuring <10 g were not recorded. | Linear head acceleration (g) Rotational head velocity (rad/s^2^) | Not reported |
| Brooks et al. [69] | Cohort 36 female youth soccer players Age: 13.4 ± 0.9 years | Game scenario (corner kick, deflection, punt, goal kick, pass in the air, free kick, throw-in) | Ball delivery - kicks/throws/deflections etc during live game play Ball speed(s) and characteristics - not controlled Header type(s) - live headers during game play confirmed via video analysis Number of headers - not controlled Data collection - wireless sensors (GForce Tracker; Artaflex Inc., Markham, Ontario, Canada) secured at the back of the head with a headband. A 7 g trigger threshold was used. The sensors recorded data from 8 ms preceding the triggered threshold and 32 ms after it. | Linear acceleration (g) Angular velocity (rad/s) | Not reported |
| Caccese et al. [70] | Cohort  16 female NCAA Div I collegiate soccer players  Age: 19.6 ± 1.0 years | Game scenario: (bounce, secondary header, punt, throw-in, goal kick, corner kick, kick (base variable)) | Ball delivery - kicks/throws/deflections etc during live game play Ball speed(s) and characteristics - not controlled Header type(s) - live headers during game play confirmed via video analysis Number of headers - not controlled Data collection - Triax SIM, SIM-G model (Triax Technologies Inc., Norwalk, CT) and custom headband. The SIM, once in the headband, was positioned at the back of the athlete’s head around the nuchal line. The SIM contains a high-g and low-g triaxial accelerometer to measure linear acceleration levels within a 3–150 g range and a triaxial gyro to measure angular head motion. A 10 g threshold was set on the accelerometers. | Peak linear acceleration (g) Peak rotational acceleration (krad/s^2^) | Yes - centre of gravity of the head. |
| Caccese et al. [61] | Cross-sectional 100 soccer players (58 F, 42 M) Age: 17.1 ± 3.5 years | Head and neck size Neck strength Sex | Ball delivery - JUGS soccer machine (JUGS, Tualatin, OR, US) from 12m distance Ball speed(s) and characteristics - 11.2 m/s at 40°; size 5 balls, 450 g, inflated to 9 psi Header type(s) - standing headers, headed at a target located approximately 2 m in front of them Number of headers - 12 (10 measured) Data collection - heading kinematics along with the local coordinate systems for the head and triaxial accelerometer/gyroscope (SIM, Triax Technologies Inc., Norwalk, CT, US) were determined using an 8-camera motion capture system (Motion Analysis Corporation, Santa Rosa, CA, US). Data were recorded at 100 Hz with a 1/1,000 shutter speed. | Peak linear acceleration (g) Peak rotational acceleration (rad/s^2^) | Yes - head centre of gravity. |
| Caccese et al. [62] | Cross-sectional 100 active soccer players (58 F,42 M) Youth female (n=18) // male (n=8) Age: 12.8 ± 0.9 // 13.0 ± 0.9 years High school female (n=19) // male (n=14) Age: 16.7 ± 0.9 // 16.9 ± 1.0 years Collegiate female (n=21) // male (n=20) Age: 19.3 ± 1.1 // 20.8 ± 1.3 years | Sex Age | Ball delivery - JUGS soccer machine (JUGS, Tualatin, OR, US) from 12m distance Ball speed(s) and characteristics - 11.2 m/s at 40°; size 5 balls, 450 g, inflated to 9 psi Header type(s) - standing headers, headed at a target located approximately 2 m in front of them Number of headers - 12 (10 measured) Data collection - triaxial accelerometer/gyroscope (SIM-G, Triax Technologies, Norwalk, CT), secured to the back of the head using a custom tight-fitting elastic cap. Signals from the accelerometer and gyroscope were sampled at 1000 Hz. | Peak linear acceleration (g) Peak rotational acceleration (rad/s^2^) | Yes - head centre-of-mass. |
| Cheever et al. [53] | Non-randomised study of interventions 40 current female collegiate soccer athletes Age: 18-25 years | Effect of fatigue intervention | Ball delivery - JUGS soccer machine (Jugs Sports Inc, Tualatin, OR) Ball speed(s) and characteristics - 25 mph; standard size 5 soccer ball Header type(s) - not reported Number of headers - 5 Data collection - gForce tracker (gForce Tracker INC, Richmond Hill, ON, Canada) head impact monitoring system. Head impact data were collected as an observational measure to control for resultant head accelerations between groups and provide context to the magnitude of impacts being administered. | Peak linear acceleration (g) Peak rotational acceleration (rad/s^2^) | Not reported |
| Chrisman et al. [44] | Cohort 46 youth soccer players (25 F, 21 M) Age: 11-14 years | Age Sex | Ball delivery - kicks/throws/deflections etc during live game play Ball speed(s) and characteristics - not controlled Header type(s) - live headers independently confirmed by an observer Number of headers - not controlled Data collection - head impacts were measured using the xPatch. The xPatch is an adhesive-mounted device that is placed over the mastoid bone and measures triaxial linear acceleration of head impacts. The device measures acceleration continuously at a frequency of 1 kHz and records when it measures an impact greater than 10 g (set threshold). If an impact exceeds the threshold, the device saves 10 ms before that impact and 90 ms after the impact, providing X, Y, and Z coordinates of linear acceleration at 1 ms intervals. | Linear acceleration (g) | Not reported |
| den Hollander et al. [71] | Cohort/Observational descriptive 31 elite/professional players (16 F, 15 M) Age: F: 14-18 years; M: 22-33 years | Age  Sex | Ball delivery - kicks/throws/deflections etc during live game play Ball speed(s) and characteristics – not controlled Header type(s) - live headers confirmed by video analysis Number of headers – not controlled Data collection – ACT Head Impact Tracker (Northern Sports Insight and Intelligence Oy). The tracker measured any impacts to the head over a force of 10 g. Each observed header was noted and the timestamp of the header and were aligned to timestamps in the video footage. | Head acceleration (g) | Not reported |
| Doewes et al. [55] | Non-randomised study of interventions - one group pretest-post-test design 3 female football players  Age: 18-22 years | 8-week neck flexor and extensor strengthening intervention | Ball delivery – stationary pendulum header Ball speed(s) and characteristics – stationary ball, ball characteristics not defined Header type(s) - standing, jumping and running headers headed horizontally forward as hard as possible Number of headers – not reported Data collection - Kinovea software - video analysis. No further details provided. | Angular velocity (rad/s)  Angular acceleration (rad/s^2^) | Not reported |
| Dezman et al. [64] | Cross-sectional 16 subjects (8 F, 8 M) Age: 20.5 ± 1.9 years | Neck strength | Ball delivery - hand thrown from 3m distance Ball speed(s) and characteristics - mean velocity of 4.29 m/s; 450 g ball inflated to 82.7 kPa Header type(s) - headed back to the throwers hands Number of headers - approx. 20 headers for 5 useable recordings Data collection - 14-camera Vicon MX3 Motion Capture System (Vicon Motion Systems, Los Angeles, California). A stationary marker set was used to estimate the sampling error at 450 Hz. To record motion, 11 retro-reflective markers were attached to anatomic landmarks. Both temples (left and right front of head; LFH, RFH) and along the parietal/occiput suture (left and right back of head; LBH, RBH) were marked and provided data for kinetic analysis of head acceleration. The soccer ball had 6 low profile soft markers attached, enabling simultaneous recording of ball speed and subject movement. | Linear (translational) acceleration (m/s^2^) Angular acceleration (rad/s^2^) | The numerical derivative of each velocity vector was used to calculate component acceleration vectors for each marker. Because the skull is a rigid body, the component acceleration vectors were averaged to find a head acceleration vector. |
| Dorminy et al. [87] | Pre-test post-test design 16 collegiate soccer players (6 F, 10 M) Age: 20.44 ± 0.24 years | Ball speed 30, 40 or 50 mph | Ball delivery - JUGS soccer machine approximate angle of projection 40 degrees Ball speed(s) and characteristics -30, 40 or 50 mph; size 5 ball inflated to 8 psi Header type(s) - straight-on directed at a target in the air Number of headers - 5 Data collection – a custom-fit mouthpiece with accelerometer (model 35A, Endevco Corporation, San Juan Capistrano, CA, USA) was used to assess head acceleration data along x, y and z axes. | Linear head acceleration (g) | Not reported |
| Filben et al. [72] | Cohort 16 female NCAA Div I soccer players Age: 19.8 ± 1.24 years | Play state (i.e., the on-field scenario in which the header occurred) Intent (i.e., the objective of the player performing the header) Outcome (i.e., whether the header achieved its intended goal) | Ball delivery - kicks/throws/deflections etc during live game play Ball speed(s) and characteristics - not controlled Header type(s) - live headers during game play confirmed via video analysis Number of headers - not controlled Data collection - validated mouthpiece sensor containing an accelerometer and angular rate sensor embedded in acrylic and custom-fit to each participant using 3D dental scans (TRIOS intraoral scanners, 3Shape A/S, Copenhagen, Denmark). The mouthpieces were configured to collect 60 milliseconds (ms) of data (15 ms pre-trigger and 45 ms post-trigger) whenever a 5 g linear acceleration was detected along any axis for at least 3 ms. | Linear acceleration (g) Rotational acceleration (rad/s^2^) Rotational velocity (rad/s) | Yes - head centre of gravity. |
| Filben et al. [73] | Cohort 19 female soccer players 6 youth Age 15.27 ± 0.11 years 13 collegiate Age: 20.19 ± 1.34 years | Level of play Session type | Ball delivery - kicks/throws/deflections etc during live game play Ball speed(s) and characteristics - not controlled Header type(s) - live headers during game play confirmed via video analysis. Ball delivery method was defined as the method by which the ball was delivered to the player prior to the header. These were kicks, throws, and headers. Number of headers - not controlled, live headers.  Data collection - mouthpiece sensor outfitted with a tri-axial accelerometer and gyroscope. The mouthpiece was custom fitted to a 3D printed dental model created from a high-resolution scan (3shape, Copenhagen, DK) of the upper dentition. Kinematic data and time-synchronized video data were collected during each game and practice. Sensors were configured to collect 60 ms of data (15 ms pre-trigger and 45 ms post-trigger) at a sampling frequency of 4681 Hz when the linear acceleration magnitude exceeded 3.5 g (youth) or 5 g (collegiate) for at least 3 ms along any axis. | Linear acceleration (g) Rotational acceleration (krad/s^2^) Rotational velocity (rad/s) | Not reported |
| Filben et al. [45] | Cohort Female NCAA Division I soccer players, 24 player season (from 14 unique players)  Age: 19.8 ± 1.24 years | Head impact location Header type (standing versus jumping) Player position Session type Ball delivery | Ball delivery - kicks/throws/deflections etc during live game play Ball speed(s) and characteristics - not controlled Header type(s) - live headers verified with time-synchronized video  Number of headers - not controlled Data collection - mouthpiece sensor containing a triaxial accelerometer and gyroscope. The mouthpieces were fit to each individual player by obtaining a dental scan of each player’s upper dentition (TRIOS intraoral scanners; 3Shape A/S, Copenhagen, Denmark). The mouthpieces were configured to trigger recordings whenever the accelerometer exceeded a 5 g threshold for at least 3 ms along any axis. | Linear head acceleration (g) Rotational head acceleration (krad/s^2^) Rotational velocity (rad/s) | Yes - head centre of gravity. |
| Filben et al. [88] | Cohort  14 female soccer players  Age: 14.4 ± 0.9 years | Heading technique  Ball delivery  Session type (practice versus. game) | Ball delivery - kicks/throws/deflections etc during live game play Ball speed(s) and characteristics – not controlled Header type(s) - live headers, film verified.  Number of headers – not controlled Data collection – instrumented mouthpieces. Mouthpieces recorded 60 ms of tri-axial linear acceleration and rotational velocity at 4684 samples per second (sps) and 1565 sps, respectively, when the accelerometer detected linear acceleration of 5 g for 3 ms on any axis. | Peak linear acceleration (g)  Peak rotational acceleration (rad/s^2^)  Peak rotational velocity (rad/s) | Yes - head centre of gravity. |
| Gutierrez et al. [65] | Cross-sectional 17 female varsity high school players Age: 15.9 ± 0.9 years | Neck strength | Ball delivery - standard throw-in from 30 ft Ball speed(s) and characteristics - not defined/controlled Header type(s) - directed to the left, right or back to the thrower Number of headers - 15 (5 in each direction) Data collection - subjects were equipped with a custom headband, which held a triaxial accelerometer (Type 8690C5, Kistler Instrument Corp., Amherst, NY) to the back of their head (opposite from where they will be heading the ball). The accelerometer measured impact in three planes—anterior/posterior, right/left, and superior/inferior. | Peak linear acceleration (g) | Not reported |
| Hanlon and Bir [89] | Cohort 24 youth girls soccer players Age: U14 | Location of header (left, right, top, front, or back of head) | Ball delivery - kicks/throws/deflections etc during live game play Ball speed(s) and characteristics - not controlled Header type(s) - live headers confirmed by video recording Number of headers - not controlled Data collection - HITS headgear; a snug fit was required to maintain accelerometer contact with the back of the head. The system has six (T250 g) single-axis linear accelerometers (Analog Devices, Inc., Norwood, MA) placed tangentially to the head to measure both linear and angular accelerations during an impact event. Data were recorded at 1000 Hz for each of the six separate scrimmages. When any event exceeds 10 g, the system triggered and recorded 8 ms before the event and 32 ms after the event. | Linear head acceleration (g) Angular head acceleration (rad/s^2^) | Not reported |
| Harriss et al. [90] | Cohort 36 female elite youth soccer players Age: 13.4 ± 0.9 years | Head impact location Game scenario (pass in air, throw in, deflection, punt, shot, goal kick, corner) | Ball delivery - kicks/throws/deflections etc during live game play Ball speed(s) and characteristics - not controlled Header type(s) - live headers matched with video recordings Number of headers - not controlled Data collection - wireless sensors (GForce Tracker, Artaflex Inc., Markham, Ontario, Canada) at the back of the head that were secured with a headband. The GForce Tracker sensors contains a tri-axial accelerometer and a tri-axial gyroscope that measure linear acceleration, and rotational velocity. The sensors triggered when head impacts exceeded a linear acceleration of 7 g. The devices recorded 8 ms of data preceding the threshold and 32 ms of the data following the threshold. Linear accelerations were sampled at 3000 Hz and filtered through an onboard analog low-pass filter with a cutoff frequency of 300 Hz. Rotational velocity was sampled at 800 Hz, and low pass filtered with a cutoff frequency of 100 Hz. | Peak linear acceleration (g) Peak rotational velocity (degrees/s) | Not completed |
| Huber et al. [50] | Randomised control trial  19 soccer players (2 F, 17 M) Age: 15.7 ± 0.4 years | Type of header: frontal versus. oblique headers | Ball delivery – JUGS ball launcher from 10 m away  Ball speed(s) and characteristics – 11.2 m/s; size 5 soccer ball (450 g) inflated to 12 psi Header type(s) - frontal heading participants headed forwards directly toward the launch direction, oblique heading participants directed the ball at a target 90° to the right of the launch direction Number of headers - 10 Data collection – Prevent Biometrics Impact Monitoring Mouthguard (IMM) which comprises a triaxial linear accelerometer and triaxial gyroscope measuring both linear acceleration (6200 g in each axis) and angular velocity (635 rad/s in each axis) at 3200 Hz. Premade boil-and-bite IMMs were individually fit for a single athlete. The IMM sensor recorded linear acceleration and angular velocity for all three axes for 50 ms (pretrigger, 10 ms; posttrigger, 40 ms) when linear acceleration measured in any axis exceeded 5 g. Headers were video verified. | Peak linear acceleration (g) Peak angular velocity (rad/s) Peak angular acceleration (rad/s^2^) | Yes – head centre of gravity |
| Kalichova and Lukasek [91] | Controlled laboratory study - one off measurement 63 footballers of four age categories U11 – U17.  16 U11 players with average age of 10.3 years  15 U13 footballers with average age of 12.4 years  18 U15 players with average age of 14.4 years  14 U17 players with average age of 16.3 years | Age Ball drop height (ball velocity) | Ball delivery - hand dropped from 20 cm in front of the players head Ball speed(s) and characteristics -drop heights of 0.5 m, 1.0 m, and 1.5 m; size 4 for U11/U13 and size 5 for U15/U17 Header type(s) - to head the ball as far as possible maintaining foot contact with the ground Number of headers - one successful attempt at each height (no average number of total headers) Data collection - a sensitive unit MPU-6000 is placed on the board containing a tri-axial accelerometer and tri-axial gyroscope. We chose to attach the device on the vertex of head using a specially created headband. The accelerometer unit was inserted into a tight pocket sewed into the circumference band located at occipital bone so that a positive direction of one of the axes led from the forehead to the back of the head. | Linear acceleration (g) | Not reported |
| Kenny et al. [74] | Cohort 13 female university varsity soccer players Age: 19.9 ± 1.6 years | Type of header Location of head impact Session type Player position | Ball delivery - kicks/throws/deflections etc during live game play Ball speed(s) and characteristics - not controlled Header type(s) - live headers confirmed via video analysis Number of headers - not controlled Data collection - mouthpieces containing a tri-axis accelerometer, to measure translational acceleration in the anterior/posterior (X), left/right (Y) and superior/inferior (Z) directions, and a tri-axis gyroscope, to measure angular velocity in the coronal (X), sagittal (Y), and horizontal (Z) planes. The mouthpieces were set to trigger impact recording when the sensor detected over 5 g linear acceleration for at least 2 ms in any of the X, Y or Z axes. | Peak linear acceleration (g) Peak angular acceleration (rad/s^2^) Peak angular velocity (rad/s) | Yes - head centre of gravity. |
| Lamond et al. [75] | Cohort 23 NCAA Division I female players Age: 19.7 ± 1.2 years | Playing position Playing scenario | Ball delivery - kicks/throws/deflections etc during live game play Ball speed(s) and characteristics - not controlled Header type(s) - live headers confirmed and manually recorded by researcher observation Data collection - Smart Impact Monitor (SIM; firmware version 3.7; SIM-G, version 3.3; AP, version 0.9.150413; software, Triax Technologies, Norwalk, CT). The SIM contains a low-g and high-g triaxial accelerometer to measure linear accelerations and a gyroscope to measure the rotational velocity of the head. Each participant was assigned a SIM and a custom headband and once fitted with the SIM, was positioned around the nuchal line. | Linear acceleration (g) | Yes - centre of gravity of the head. |
| Larson [92] | Controlled laboratory study - one off measurement 13 Division II soccer athletes (7 F, 6 M) Age: 18-22 years | Type of header High clearing versus. driven versus flick-on Neck strength Sex | Ball delivery - JUGS soccer machine from 35 ft distance Ball speed(s) and characteristics - 25 mph Header type(s) - high clearing (JUGS angle 42.1 degrees), driven (JUGS angle 26.8 degrees) and flick-on (JUGS angle 26.8 degrees).  Number of headers - 9 Data collection - tri-axial accelerometer screwed on to the back of a standard water polo cap (with chin strap, but ear caps removed) through a 1/4-inch-thick piece of foam to better mould to the back of each participants’ head. The accelerometer was affixed to the cap so that it would rest at the centre back of the participants’ heads. The MicroStrain G-Link tri-axial wireless accelerometer is compact in size (25mm x 25 mm x 5 mm), weighs only 47 grams, has 2 MB of on-board memory storage, and runs off a 9-volt battery (MicroStrain, 2010). | Linear acceleration (g) | Not reported |
| Liberi [93] | Controlled laboratory study - one off measurement 16 collegiate male varsity soccer players (Division I) Age: 19.5 years | Wet ball versus dry ball | Ball delivery -mechanical leg at a distance of 18.5 m, and a projection angle of 32 degrees Ball speed(s) and characteristics - 15.5 m/s; standard psi Header type(s) - headed back to the machine Number of headers - 6 Data collection - a triaxial quartz beam accelerometer was used to measure acceleration of the skull in X and Y directions. These axes defined the sagittal plane of the skull. The accelerometer was mounted on an adjustable nylon headband that was fitted to ensure that the accelerometer was placed tightly over the inion. | Linear head acceleration (g) | Not reported |
| Ludwig [66] | Cross-sectional 24 female collegiate soccer players Frequent headers group Age: 24.2 ± 3.4 years Infrequent headers group Age: 25.4 ± 4.2 years | Frequent headers versus infrequent headers | Ball delivery - ball catapult  Ball speed(s) and characteristics - 8 m/s, soccer ball inflated to 12 psi Header type(s) - standing header as fast and accurately back in the direction of the ball catapult low as if trying to score a goal Number of headers - 13 (10 measured trials) Data collection - Peak5 motion measurement system. The Direct Linear Transformation method was used to determine 3D coordinates. Each participant was videotaped by four Panasonic AG-450 camcorders which were genlocked for synchronization. Four Panasonic AG-1730 Super VHS videocassette recorders were used to record the signal from the four cameras. Analysis was performed using a Daly 486 computer with Peak5 software, a VCR controller board and a video frame grabber board. | Average linear acceleration | Not reported |
| Lukasek and Kalichova [94] | Controlled laboratory study - one off measurement 16 male youth footballers (U11) Age: 10 years | Ball drop height/ball velocity | Ball delivery - ball was hand dropped Ball speed(s) and characteristics - 0.5 m (3.13 m/s), 1 m (4.43 m/s) and 1.5 m (5.43 m/s); size 4 ball Header type(s) - not reported  Number of headers - 3 Data collection - triaxial accelerometer and gyroscope capable of collecting data at 1000 Hz and stored on mini SD card. The range is ± 16g and it enables to measure rotation 2000 degree/s per axis. | Head acceleration | Not reported |
| Miller et al. [76] | Cohort 7 U14 female soccer players Age: 13.4 ± 0.6 years | Ball delivery method | Ball delivery - kicks/throws/deflections etc during live game play Ball speed(s) and characteristics - not controlled Header type(s) - live headers confirmed using video analysis Number of headers - not controlled Data collection - custom-instrumented mouthpiece. Impressions of the upper dentition were collected for each athlete by a local dentist. Dental moulds were poured from each impression and used to custom-fit each mouthpiece. Each mouthpiece features a tri-axial accelerometer and gyroscope embedded within a rigid retainer. Five of the mouthpieces used the sensor and battery components of an xPatch sensor (X2 Biosystems, Seattle, WA, USA) and the remaining two used sensor components developed by Stanford. | Peak linear acceleration (g) Peak angular acceleration (rad/s^2^) Peak angular velocity (rad/s) | Yes - head centre of gravity. |
| Muller and Zentgraf [59] | Non-randomised study of interventions 37 players  Age: 15-18 years  22 players (7 F, 15 M)  28 participants involved in the intervention section of the study 14 intervention (8 F, 6 M) 14 usual training group (5 F, 9 M) | Neck strength Sex 14-week neck strengthening and neuromuscular training intervention | Ball delivery -ball machine (Freddie MAX, JofoSport, Czech Republic) from 10-15 m distance Ball speed(s) and characteristics - 9.4 m/s and 10.8 m/s; size 4 ball, 350 g, a barometric pressure of 80 kPa Header type(s) - stationary frontal headers Number of headers - 12 Data collection - two 3D accelerometers assessed head kinematics during ball impact by capturing data with a sampling frequency of 1.2 kHz (Noraxon, Scottsdale, AZ). We fixed 1 accelerometer to the proximal Os sternum using an adhesive tape, a second, above the Os occipitale using a headband. | Peak linear acceleration (g) | Not reported |
| Narimatsu et al. [77] | Controlled laboratory study - one off measurement 11 male high school soccer players Age: 16.8 years | Effect of clenching with and without a mouthguard | Ball delivery - JUGS machine from approx. 9 m distance Ball speed(s) and characteristics - initial velocities of machine set at 28.0 (right motor) and 38.0 (left motor) m/s  Header type(s) - standing, instructed not to contribute actively to the impact Number of headers - 15 Data collection - a triaxial accelerometer and a multi-channel telemetry system. The accelerometer was put into a headband to measure head acceleration. | Linear acceleration (g) | Not reported |
| Naunheim et al. [95] | Controlled laboratory study - one off measurement 4 male subjects  Age: 25-36 years | Ball speed 9 m/s versus 12 m/s | Ball delivery - mechanical soccer ball driver (Soccer Tutor, Burbank, CA) from a 6m distance and 1.2 m from the ground Ball speed(s) and characteristics - 9 m/s and 12 m/s; 450 g soccer balls, 8 psi Header type(s) - headed back to the machine Number of headers - 6 Data collection - Three triaxial accelerometers (PCB Model 356B11, PCB Piezotronics, Inc., Depew, NY) were mounted at known locations on the fairly rigid polyethylene headpiece. The headpiece was fitted to the head of each subject, so that the sagittal plane of the subject’s head and the X-Z plane of the accelerometer array were coincident. Two thick latex rubber bands were then stretched over the headpiece. One band was stretched under the chin and the second over the upper lip to firmly preload the headpiece against the skull. A fourth triaxial accelerometer was glued to a plastic mouthpiece inserted in the subject’s mouth. | Linear head acceleration (m/s^2^) Angular head acceleration (rad/s^2^) | Yes - heads centre of mass. |
| Nevins et al. [46] | Cohort 23 high school soccer players (15 F, 8 M) Females Age: 15.33 ± 1.01 years Males Age: 16.75 ± 1.09 years | Sex | Ball delivery - kicks/throws/deflections etc during live game play Ball speed(s) and characteristics - not controlled Header type(s) - live headers as identified in video recordings Number of headers - not controlled Data collection - xPatch head impact sensors (X2 Biosystems, Seattle, WA), where head impact exposure was quantified by the magnitude (peak resultant linear [PLA] and peak resultant angular acceleration [PAA]) and frequency of head impacts. The xPatch device used a combination of a triaxial accelerometer and a triaxial gyroscope to obtain linear and angular acceleration in a small form factor, which allowed the device to be worn during soccer games. Each sensor was mounted behind the right ear, just above the mastoid process, using a double-sided adhesive provided by the manufacturer. | Peak linear acceleration (g) Peak angular acceleration (rad/s^2^) | Not reported |
| Omdal [51] | RCT 8 high school female soccer players (4 intervention, 4 control) Age: 16.24 ± 1.07 years | The effect of a sports specific plyometric and functional training program | Ball delivery - balls thrown from 3m away from three different reference points (-60, 0, 60 degrees from straight ahead position) Ball speed(s) and characteristics - estimated 6 m/s; 'standard measurement' ball pressure kept constant Header type(s) - headed back to the thrower with maximal effort Number of headers - 15 (5 from each reference point) Data collection - head impact acceleration values were recorded with a 24G Accelerometer (3D DTS, Noraxon, USA) placed on the posterior aspect of the skull, held against the head using a headband, synced with the EMG system. | Peak linear acceleration (g) | Not reported |
| Peek et al. [78] | Controlled laboratory study - one off measurement 61 youth soccer players (26 F, 35 M) Age: 14.52 ± 1.37 years | Ball characteristics - inflation pressure, mass and characteristics 1. KickerBall, size 5 192 g and 5.00 psi 2. Adidas Starlancer, size 5, 432 g, 5.00 psi 3. Heading-pro, size 4, 255 g, 5.00 psi 4. Deploy Envision, size 5, 430 g, 10.50 psi | Ball delivery - overhead throw-in from 5 m Ball speed(s) and characteristics - average 6.25 m/s; different ball characteristics under investigation Header type(s) - standing header back to the feet of thrower Number of headers - 9-12 Data collection - a close fitting elastic ‘swim cap’ which housed a motion sensor (AX6™, Axivity™, Newcastle, UK), measuring linear acceleration (tri-axial accelerometer) and angular velocity (tri-axial gyroscope), was placed on the player’s head, ensuring that the accelerometer was positioned centrally at the base of the occiput. | Peak linear head acceleration (g) Peak angular velocity (dps) | No transformation, noted as a limitation |
| Peek et al. [17] | Pilot randomised control trial 52 players completed the study (31 intervention, 21 control) Intervention group (14 F, 17 M) Age: 14.35 ± 0.29 years Control group (7 F, 14 M) Age: 14.95 ± 0.26 years | The effect of adding neck exercises to the FIFA 11+ | Ball delivery - overhead throw-in from 5m  Ball speed(s) and characteristics - average 6.25 m/s; size 5 ball inflated to 5 psi Header type(s) - headed back to the throwers feet Number of headers - 2-5 Data collection - players wore a close-fitting latex ‘swim cap’ that housed an IMU (AX6™, Axivity™, Newcastle, UK) to measure head impact magnitude: linear acceleration (tri-axial accelerometer) and angular velocity (tri-axial gyroscope). The swim cap was placed on the player’s head with the IMU positioned centrally at the base of the occiput. | Peak linear acceleration (g) Peak angular velocity (dps) | Not reported |
| Pereira et al. [79] | Randomised repeated measures 17 recreational adult football players (3 F, 14 M) Age: 22 ± 3.5 years | Low-pressure ball (58.6 kPa; 8.5 psi) versus headers with high-pressure ball (103.4 kPa; 15.0 psi)  Peak isometric neck flexor and extensor strength  Sex | Ball delivery – ball launching device, each ball was delivered at head height on a downward arc to simulate a free play header Ball speed(s) and characteristics – 30 kph; size 5 match ball at either the low or high-pressure condition Header type(s) - rotational headers – ball redirected perpendicular to its initial trajectory aiming for a small goal 90° to their left Number of headers - 20 Data collection - An inertial measuring unit (IMU) (Blue Trident) was used to measure linear acceleration (tri-axial accelerometer) and angular velocity (tri-axial gyroscope) of the head during each header. This device was placed centrally at the base of the occiput and secured using a close-fitting elastic ‘swim cap’. | Peak linear acceleration (g) Peak angular velocity (rad/s) | No transformation, noted as a limitation. |
| Pritchard et al. [47] | Cohort 8 female youth soccer players Age: 13.5 ± 0.11 years | Type of header: standing versus jumping  Session type: game versus practice | Ball delivery - kicks/throws/deflections etc during live game play Ball speed(s) and characteristics - not controlled Header type(s) - live headers, video verified Number of headers – not controlled Data collection - custom-fit mouthpiece instrumented with a tri-axial accelerometer and gyroscope. Sensors were configured to record 60ms of data (15 ms pre-trigger and 45ms post-trigger) when the accelerometer measured values exceeding 5g (first season) or 3.5g (second season) along any single axes for 3ms or longer. The sensor threshold was lowered in the second season to increase the sensitivity of the device to collect more head impact events. | Linear acceleration (g) Rotational velocity (rad/s) Rotational acceleration (rad/s^2^) | Yes - head centre of gravity |
| Robinson [101] | Mixed lab based and live game play data collection 14 varsity women's soccer players | Type of header - passing versus clearing versus shooting and stationary versus running Age Heading proficiency | Ball delivery - underhand throw from 5, 10 and 20 yards Ball speed(s) and characteristics - not specifically controlled Header type(s) - passing, clearing, and shooting. Stationary and running. Number of headers - 35 Data collection - wearable sensor devices, developed by X2 Biosystems (xPatch), gathered 6-axis impact acceleration. The xPatch is a wearable sensor that contains a gyroscope to calculate rotational forces with three degrees of freedom, a high-G 3-axis accelerometer to calculate translational forces with three degrees of freedom, and a radio transmitter to relay the information in real time. In all sessions, the xPatch was worn behind the right ear of the participant, affixed with an adhesive bandage. | Peak linear acceleration (g) Peak rotational acceleration (deg/s^2^) Peak rotational velocity (deg/s) | Not reported |
| Sandmo et al. [96] | Controlled laboratory study - one off measurement 6 male youth soccer players  Age: 15.3 ± 0.3 years | Type of header | Ball delivery - heading drills performed in training Ball speed(s) and characteristics - not controlled Header type(s) - finishing headers, redirectional headers, long direct headers, short direct headers, and headers from in-air duels Number of headers - not controlled Data collection - MV1 (MVTrak) is a sensor system designed for custom moulded placement in the left external ear canal to optimize coupling to the head. The sensor samples linear acceleration and rotational velocity data at 1000 Hz, filtering the data with a phaseless 300-Hz 8-pole low-pass Butterworth filter to remove noise; rotational acceleration is calculated by differentiating these filtered rotational velocity data. The sensor then provides a time-stamped output of peak linear acceleration (PLA), peak rotational velocity (PRV), and peak rotational acceleration (PRA) for all accelerative events exceeding 3g (ie, nominal head impacts), followed by a 250-millisecond latency period before another impact can be registered. | Peak linear acceleration (g) Peak rotational acceleration (rad/s^2^) Peak rotational velocity (rad/s) | Not reported |
| Saunders et al. [48] | Cohort  28 NCAA Division III soccer players (16 F, 12 M)  Females Age: 19.94 ± 1.06 years Males Age: 20.25 ± 1.14 years | Sex  Game versus practice | Ball delivery - kicks/throws/deflections etc during live game play Ball speed(s) and characteristics - not controlled Header type(s) - live headers confirmed using video analysis Number of headers - not controlled Data collection - the X2 Biosystem (Seattle, WA) xPatch sensor measured the magnitude and frequency of head impacts. The xPatch contains a triaxial accelerometer and gyroscope that calculates the linear acceleration (g) and rotational acceleration (deg/s2) every time the head is impacted. We placed the xPatch over the participants' right mastoid process using an adhesive patch from X2 Biosystems and Cavilon (3M, St. Paul, MN) barrier film to help reduce the potential for skin irritation. The xPatch sensors measured the time of the impact, head impact location, and linear and rotational accelerations above a 10g threshold at 1 kHz and 800 Hz. | Linear acceleration (g) Rotational acceleration (deg/s^2^) | Not reported |
| Segars et al. [49] | Cohort 16 NCAA Div I female soccer players Age: 19.8 ± 1.24 years | Playing scenario: technical training versus set pieces | Ball delivery - kicks/throws/deflections etc during live game play Ball speed(s) and characteristics – not controlled Header type(s) - live headers confirmed using video analysis Number of headers - not controlled Data collection - each participant wore a previously validated custom-fit mouthpiece sensor during practices over the course of the season. The mouthpieces collected 60 milliseconds of triaxial linear acceleration and triaxial angular velocity whenever a 5g linear acceleration was detected along any axis for at least 3 milliseconds. | Peak linear acceleration (g) Peak rotational acceleration (rad/s^2^) Peak rotational velocity (rad/s) | Yes - head centre of gravity |
| Self et al. [97] | Controlled laboratory study - one off measurement 10 participants from the U.S. Air Force Academy Men's Varsity Soccer Team | Ball speed 16.5 m/s versus 12.2 m/s Header type  Straight versus redirecting/corner | Ball delivery - ball machine (JUGSTM Soccer Machine) Ball speed(s) and characteristics - 16.5 m/s and 12.2 m/s; Nike GEO Altair III balls  Header type(s) - headed back to the machine and redirecting header (90 degrees from its inflight path) Number of headers - 12 Data collection - the participants inserted two small earplugs into their ears. Each earplug contains a ±500 g's Endevco 7269 tri-axial accelerometer, which has a mass of 0.4 grams and measures approximately 11 x 8 x 5 mm. The right earplug also contained an Analog Devices ADXRS300 ±300o/s Single Chip Yaw Rate Gyro oriented along the pitch axis. All data were collected at 2000 Hz with a 500 Hz low pass filter. After inserting the earplugs, experimenters placed a single layer of pre-wrap around the head to keep the sensors in place. | Linear head acceleration (g) | Not reported |
| Shewchenko et al. [80] | Controlled laboratory study - one off measurement 7 non-professional soccer players aged 20-23 years | Ball speed 6 m/s versus 8 m/s | Ball delivery - not reported Ball speed(s) and characteristics - 6 m/s and 8 m/s Header type(s) - 10 different heading scenarios tested  Number of headers - not reported Data collection - two different types of intraoral device, capable of measuring linear and angular accelerations, to measure the head kinetics. The units were custom fitted to individuals with thermoformed bite plates to ensure good coupling with the skull. Subjects clenched the bite plate firmly in both cases. We measured linear accelerations with Endevco Model 7264–2000 piezo-resistive accelerometers and angular acceleration with an Endevco Model 7302BM4 angular accelerometer (Endevco Corp., San Juan Capistrano, CA). | Peak linear acceleration (m/s^2^) Peak angular acceleration (krad/s^2^) | Not reported |
| Shewchenko et al. [98] | Controlled laboratory study - one off measurement 3 non-professional soccer players aged 20-23 years | Ball characteristics - inflation pressure, mass and characteristics Balls tested: 1. Baseline, 444 g, 0.8 bar, Fevernova Tri-Lance  2. Low pressure 0.6 bar, Fevernova Tri-Lance  3. High pressure 1.1 bar, Fevernova Tri-Lance  4. Low mass 299 g, Fevernova Junior 290  5. Low mass 351 g, Fevernova Junior 350 | Ball delivery - not reported Ball speed(s) and characteristics - 6 m/s and 8 m/s; different ball masses and pressures as per previous column Header type(s) - 10 different heading scenarios tested Number of headers - not reported Data collection - the subjects were instrumented to measure linear and angular acceleration at the mouth with an intraoral device. | Peak linear acceleration (m/s^2^) Peak angular acceleration (krad/s^2^) | Not reported |
| Sokol-Randell et al. [81] | Cohort 10 male university soccer players Age: 20 ± 1 years | Header type  Ball delivery | Ball delivery - kicks/throws/deflections etc during live game play Ball speed(s) and characteristics – not controlled Header type(s) - live headers confirmed using video analysis Number of headers - not controlled Data collection - custom-fit iMGs (Prevent Biometrics, Minneapolis, MN). Every iMG possessed an infrared proximity sensor to assess coupling to the upper dentition, as well as an accelerometer and gyroscope, both sampling at 3200 Hz, with measurement ranges of ±200 g and ±35 rad/s, respectively. The iMGs were configured to capture 10 ms of pre-trigger data and 40 ms of post-trigger data when the accelerometer detected over 5 g linear acceleration on a single axis of the iMG. | Peak linear acceleration (g) Peak angular acceleration (krad/s^2^) | Yes – head centre of gravity |
| Stelzer-Hiller et al. [102] | Cohort 15 male university soccer players Age: 20 ± 1 years | Pitch location - inside versus outside the penalty areas | Ball delivery - kicks/throws/deflections etc during live game play Ball speed(s) and characteristics - not controlled Header type(s) - live headers confirmed using video analysis Number of headers - not controlled Data collection - Prevent Biometrics’ custom-fit iMG. The iMG incorporated a tri-axial linear accelerometer and a tri-axial gyroscope, recording linear and angular head kinematics during HAEs at a sampling rate of 3.2 kHz and over a range of ±200 g and ±35 rad/s, respectively. Data recording was triggered by a minimum HAE single-axis trigger value of 5 g and measured 10 ms of pre-trigger data and 40 ms of post-trigger data. | Peak linear acceleration (g) Peak angular acceleration (krad/s^2^) Peak change in angular velocity (rad/s) | Not reported |
| Stucker [82] | Cohort 25 Div I female collegiate soccer players Age: 19.58 ± 1.15 years | Game versus practice | Ball delivery - kicks/throws/deflections etc during live game play Ball speed(s) and characteristics - not controlled Header type(s) - live headers confirmed using video analysis Data collection - the xPatch system (X2 Biosystems, Seattle, WA). The xPatch is a small and lightweight device affixed to the participants’ head with an adhesive patch attached to the right mastoid process. It can measure linear and rotational acceleration with 6 degrees of freedom along with the location of each impact on the head. | Linear acceleration (g) Rotational acceleration (rad/s^2^) | Not reported |
| Tierney et al. [83] | Controlled laboratory study - one off measurement 44 soccer players (29 F, 15 M) Females Age: 19.5 ± 1.8 years Males Age: 20.3 ± 2.9 years | Sex Head-neck segment anthropometrics Isometric neck strength Headgear | Ball delivery -JUGS soccer machine (JUGS, Tualatin, OR) from 11m distance, 40 degrees angle of projection Ball speed(s) and characteristics - 9.83 m/s; 450 g ball inflated to 55,158 Pa Header type(s) - participants headed aiming for a target in front of them Number of headers - 12 successful headers Data collection - a custom-fit mouthpiece was fabricated for each participant by taking an impression of the teeth and making a 3-dimensional cast of the teeth and upper palate. A triaxial accelerometer (model 35A; Endevco Corp, San Juan Capistrano, CA) was secured with dental wax to the upper palate portion of the mouthpiece. The triaxial accelerometer was used to assess head acceleration data along the x-axis (anterior-posterior), y-axis (medial-lateral), and z-axis (superior-inferior). | Linear acceleration (g) | Not reported |
| Tomblin et al. [84] | Cohort 14 youth female soccer athletes Age: 12-15 years | Type of header - standing versus jumping Player position  Session type | Ball delivery - kicks/throws/deflections etc during live game play Ball speed(s) and characteristics - not controlled Header type(s) - jumping or standing live headers confirmed by time-synchronised video review Number of headers - not controlled Data collection - custom-fit mouthpiece instrumented to measure linear and rotational head kinematics during on-field impacts. Dental impressions were obtained from each athlete by a trained dental professional, and a dental model was poured from the dental impression. An accelerometer, gyroscope, inductive charging coil, processor, and battery were covered in an insulating resin epoxy and embedded in each mouthpiece. Each device was set to trigger for any event during which linear acceleration exceeded 5g for at least 3 milliseconds on any axis. Once triggered, kinematic data were saved to the mouthpiece from 15 milliseconds pre-trigger to 45 milliseconds post-trigger. | Peak linear acceleration (g) Peak rotational velocity (rad/s) Peak rotational acceleration (rad/s^2^) | Yes - head centre of gravity. |
| Wahlquist and Kaminski [60] | Non-randomised study of interventions 12 female soccer players Age: 10.5 ± 0.5 years | The Get aHEAD safely in soccer programme | Ball delivery - JUGS soccer machine (JUGS, Tualatin, OR, USA) from approximately 12.2 m away Ball speed(s) and characteristics - 11.2 m/s at a 45-degree angle; lightweight size 5 “Header Trainer” soccer balls, roughly 225 g Header type(s) - headed the ball back towards the machine Number of headers - 12 Data collection - Triax SIM-G (Triax Technologies, Norwalk, CT, USA) head impact sensor. Each head impact sensor has a triaxial accelerometer and triaxial gyroscope that measures peak linear acceleration (PLA) and peak rotational velocity (PRV) respectively. Peak rotational acceleration (PRA) is calculated from the rotational velocity. The sensor was inserted into a headband which secured the sensor to the back of the participant’s head. Each participant was fitted with the correct headband size for an optimal, snug fit. | Peak linear acceleration (g) Peak rotational velocity (rad/s) Peak rotational acceleration (krad/s^2^) | Not reported |
| Wahlquist et al. [54] | Non-randomised study of interventions 27 female youth soccer players (13 control, 14 intervention) Age: 10.8 ± 0.5 years | The Get aHEAD safely in soccer programme | Ball delivery -live headers during trainings and games Ball speed(s) and characteristics - not controlled Header type(s) - live headers Number of headers - not controlled Data collection - Triax SIM-G (Triax Technologies, Norwalk, CT) head impact sensor, secured to the back of the head using a custom, tight-fitting headband. Each sensor has a triaxial accelerometer and gyroscope that measure peak linear acceleration (PLA) and peak rotational velocity (PRV), respectively. Peak rotational acceleration (PRA) was calculated using PRV. | Peak linear acceleration (g) Peak rotational acceleration (krad/s^2^) Peak rotational velocity (rad/s) | Not reported |
| Wang [99] | Controlled laboratory study - one off measurement 8 participants (2 F, 6 M) Age: 18-30 years | Ball speed Two impact levels (low-level and high-level) | Ball delivery - pendulum impactor adjusted to participants height and weight Ball speed(s) and characteristics - soccer ball inflated to 10 psi Header type(s) - frontal impact from pendulum Number of headers - 6 Data collection - an IMU (MPU6050 from TDK InvenSense) is attached onto a bite bar affixed to a mouthguard that is custom-formed to each participant’s upper dentition mould and worn by the participant during the trials. The IMU has a sampling rate of 1 kHz and has a range of up to ±16 g of linear acceleration and up to 2000 degrees/s (or 35 rad/s) of rotational velocity. | Linear acceleration (g) Angular acceleration (rad/s^2^) Angular velocity (rad/s) | Not reported |
| Waring et al. [52] | Randomised control trial 20 varsity collegiate soccer players (12 F, 8 M) Age: 20.15 ± 1.35 years | Ball speed 11.18 m/s versus 17.88 m/s 6-week cervical strengthening program | Ball delivery - JUGS soccer machine (JUGS Inc., Tualatin, OR) from 35 m distance Ball speed(s) and characteristics - 11.18 and 17.88 m/s; size 5 ball pumped to 5,624.55 kgf/m²  Header type(s) - not reported Number of headers - 10 Data collection - inertial measurement unit (xPatch sensor, X2 Biosystems, Seattle, WA). The sensor was applied over the right mastoid process with an adhesive patch. | Peak linear acceleration (g) Peak rotational acceleration (deg/s^2^) | Not reported |
| Withnall et al. [100] | Controlled laboratory study - one off measurement 1 participant (M) Age: 30 years | Ball speed 6.4 m/s versus 8.2 m/s Headgear | Ball delivery - A powered carriage was used to launch the ball towards the subject from 5 m away Ball speed(s) and characteristics - 6.4 m/s and 8.2 m/s; size 5 ball, 430 g, ball pressure of 0.8 bar Header type(s) - head the ball to strike a target which was approximately 50x50 cm, 1 m above the floor, and 4 m away Number of headers - 40 Data collection - the subject was instrumented with a bite plate on which were mounted two orthogonal accelerometers (Endevco model 7264a-2000) to measure motion in the plane of impact. A tape switch was affixed to the surface of his forehead to serve as an electronic indicator of ball contact. For tests involving headgear, we taped this switch to the outer surface of the headgear. For bare head tests, a thin Spandex head cover was used to hold the switch in place. | Linear acceleration (g) | Data not transformed |
| Worsey et al. [67] | Cross-sectional 8 participants for the field heading tests ranged from novice to semi-professional soccer players Age: 27 ± 7.2 years Neck Muscle Activity (EMG) Intervention Test One healthy male volunteered to participate in the study. The participant had no prior experience of playing competitive soccer. | Playing experience Activation of neck muscles | Ball delivery - hand thrown from a distance of 5 m Ball speed(s) and characteristics - average velocity of the ball at point of impact was found to be 1.95 m/s; size 5 ball Header type(s) - forward header Number of headers - 10 Data collection - the inertial sensor technology used was manufactured in-house and contains a three-axis accelerometer (±16 g), three-axis gyroscope (±2000 degree/s), and magnetometer (±7 Gauss) (SABELSense, Griffith University, Nathan, Australia). The sampling rate of the sensor was set to 250 Hz. The sensor has a sync function that pulses a red LED, allowing for the user to synchronise sensor data with video footage. A 12 camera OptiTrack (Freedspace Laboratories, Melbourne, VIC, Australia) optical motion capture system sampling at 100 Hz was used as a validation tool for measurements that were recorded by the IMUs. | Head acceleration (g) | Not reported |
| *Abbreviations: BMI: body mass index, cm: centimetre, degree/s: degree per second, dps: degrees per second, DTS: direct transmission system, EMG: electromyography, ft: feet, g: gravitational acceleration, HA: head acceleration, HAE: head acceleration events, Hz: hertz, iMG: instrumented mouthguard, IMM: impact monitor mouthguard, IMU: inertial measurement unit, kgf/m²: kilogram force per square meter, kHz: kilohertz, kPa: kilopascal, kph: kilometre per hour, krad/s: kiloradian per second, krad/s^2^: kiloradian per second squared, LED: light emitting diode, m: metre, m/s: metres per second, mph: miles per hour, MPU: motion processing unit, ms: millisecond, Pa: pascal, psi: pounds per square inch, rad/s or rad/s^1^: radian per second, rad/s^2^: radians per second squared, SIM: smart impact monitor, VHS: video home system, 3D: three dimensional* | | | | | |

**Appendix VI** Header type definitions

| **Header Type** | **Definition** |
| --- | --- |
| Running header | Becker et al. [58, 68] – Moving towards a mark 3 metres in the distance, jumping with one or both legs with the ball one ball diameter above the players head |
| Standing header | Becker et al. [58, 68] – Standing shoulder width apart, no jump with the ball at the height of the forehead  Tomblin et al. [84] – Feet on the ground during ball contact  Filben et al. [45, 88] – feet are planted on the ground |
| Jumping header | Becker et al. [58, 68] – Standing shoulder width apart, jumping with both legs with the ball one ball diameter above the players head  Kenny et al. [74] and Tomblin et al. [84] – both feet off the ground during ball-to-head impact  Filben et al. [45, 88] – both feet leave the ground at the same time, contact made with the ball at the highest point |
| Frontal header variations | |
| Direct header | Sandmo et al. [96] – not defined, expected to be a header back in the same direction as ball delivery |
| Driven header | Larson [92] – participants headed the ball using the front of their upper forehead directed back to the launching machine |
| Straight header | Self et al. [97] – heading the ball back in the direction which it came from |
| Frontal header | Huber et al. [50] – headed the ball directly towards the launch direction |
| Finishing header | Sandmo et al. [96] – not defined, expected to be a header in a downwards direction towards a goal |
| Redirectional header | Sandmo et al. [96] – not defined, expected to be a header where the player changes the direction of the ball with the impact |
| Flick-on header | Larson [92] – participants headed the ball using the front and side of their upper forehead and directed it either to the left or the right of where they were standing |
| Oblique header | Huber et al. [50] – directed the ball at a target 90 degrees to the right of the launch location |
